# Redox-active, photoluminescent porous polymers based on spirofluorene-bridged *N*-heterotriangulenes and their feasibility as organic cathode materials†

**DOI:** 10.1039/d4sc04276j

**Published:** 2024-10-23

**Authors:** Angelina Jocic, Tom Wickenhäuser, Sebastian Lindenthal, Wen-Shan Zhang, Jana Zaumseil, Rasmus Schröder, Rüdiger Klingeler, Milan Kivala

**Affiliations:** a Institute of Organic Chemistry, Heidelberg University Im Neuenheimer Feld 270 69120 Heidelberg Germany milan.kivala@oci.uni-heidelberg.de; b Kirchhoff-Institute for Physics, Heidelberg University Im Neuenheimer Feld 227 69120 Heidelberg Germany; c Institute for Physical Chemistry, Heidelberg University Im Neuenheimer Feld 253 69120 Heidelberg Germany; d BioQuant Im Neuenheimer Feld 267 69120 Heidelberg Germany

## Abstract

Novel microporous polymers were synthesized through Yamamoto polymerization of selectively brominated spirofluorene-bridged *N*-heterotriangulenes. Extensive characterization, including combustion analysis, ToF-SIMS, IR, and Raman spectroscopy, confirmed the elemental composition and integrity of the polymers. The amorphous polymers, observed by scanning electron microscopy as globular particles aggregating into larger structures, exhibited remarkable thermal stability (decomposition temperatures > 400 °C) and BET surface areas up to 690 m^2^ g^−1^. Dispersions of the *tert*-butyl-substituted polymer in different solvents displayed bathochromically shifted emission with remarkable solvatochromism. The polymer is reversibly oxidized at +3.81 V (*vs.* Li/Li^+^) in composite electrodes with carbon black and reaches specific capacities up to 26 mA h g^−1^ and excellent cycling stability when implemented as cathode material in lithium-ion batteries. Our results highlight the potential of spirofluorene-bridged *N*-heterotriangulenes as versatile building blocks for the development of functional redox-active porous polymers.

## Abstract 2

Redox-active microporous polymers were fabricated from functionalized spirofluorene-bridged *N*-heterotriangulenes. The polymers exhibited a capacity of 26 mA h g^−1^ with 80% capacity retention over 400 cycles when applied as cathode material in Li-ion batteries.

## Graphical abstract

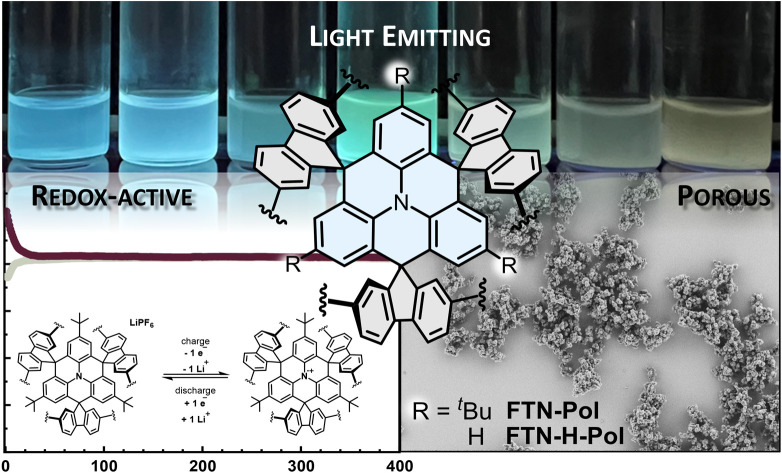

## Introduction

The development of novel energy storage materials is of utmost importance given the seriously increasing environmental and societal constraints of a growing population.^1^ Lithium-ion batteries, which emerged back in the early 1980s, are nowadays receiving enormous attention.^2^ In particular, the development of high-capacity cathode materials with low production costs and reduced environmental impact has been in the focus of research efforts.^3^ Next to conventional cathode materials based on inorganic compounds,^4^ redox-active organic compounds are continuously gaining importance due to their ready tunability through molecular design.^5^ In this context, organic compounds decorated with redox-active functional moieties such as amines,^6^ nitroxide radicals,^7^ disulfides,^8^ and carbonyls^9^ have become the most common. Polymerization of these molecular entities towards porous networks is a powerful strategy to achieve an efficient fixation and morphological stabilization of the redox-active moieties.^10^

Porous organic polymers are in contrast to the individual redox-active monomers insoluble in common organic solvents, which prevents the dissolving process into the electrolyte solution during the charge/discharge cycles and consequently enhances the charge storage capacity. A well-established strategy to synthesize redox-active polymers is the nickel-mediated Yamamoto homocoupling using halogenated aryl precursors.^11,12^ However, the inherent irreversibility of this polymerization method often results in the formation of amorphous, powdery materials with ill-defined morphologies.

**Fig. 1:**
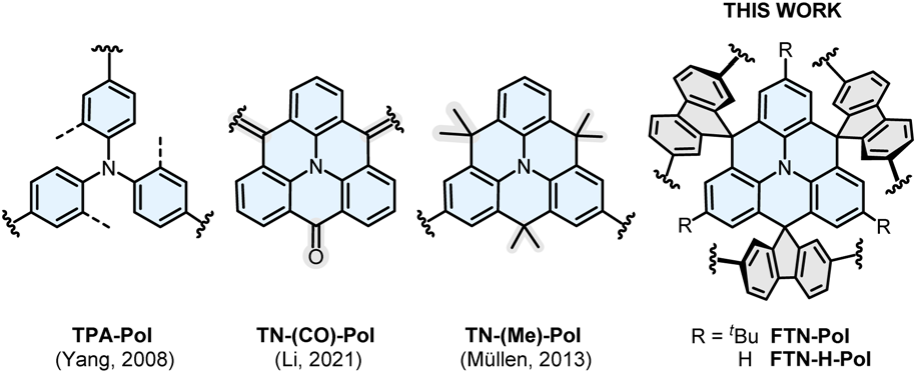
Overview of known redox-active TPA- and *N*-HTA-based polymers reported to date and the molecular cut-out of the spiro-bridged *N*-HTA-based polymers FTN-Pol and FTN-H-Pol reported herein.

Triphenylamine (TPA)-based materials have been intensively studied as versatile p-type semiconductors in organic photovoltaics (OPVs),^13^ organic light emitting diodes (OLEDs),^14^ or organic field-effect transistors (OFETs)^15^ due to their excellent redox and hole transport properties. Moreover, due to their electron-rich character and electrochemical reversibility with well-defined oxidation potentials, TPA-based materials have gradually gained attention in the field of charge storage devices.^16^ The first polytriphenylamine (TPA-Pol) used as cathode material was synthesized directly from unsubstituted TPA by Yang and coworkers in 2008 using a simple oxidative polymerization strategy with FeCl_3_ (Fig. 1). The resulting polymer displayed high specific capacities up to 103 mA h g^−1^ and an impressive capacity retention of 90% after 1000 cycles.^17^ Nevertheless, while TPA-Pol showed good charge/discharge performance, rather broad redox half-waves were observed. Given the *ortho*- and *para*-directing properties of the central nitrogen atom in TPA, the polymer formed under oxidative conditions consists of a mixture of differently linked TPA units. The resulting structural inhomogeneity has a negative impact on the electrochemical properties of the polymer.^17^

Upon introduction of different *ortho*-bridging units into TPA, such as carbonyl,^18^ dimethylmethylene,^19^ or ether,^20^ its originally propeller-shaped conformation is rendered planar in the resulting compounds, so-called *N*-heterotriangulenes (*N*-HTAs). The planarization leads to enhanced stabilization of the nitrogen-centered radical cations due to efficient spin and charge delocalization over the entire π-system.^21,22^ In 2021, Li and coworkers reported the first polymer based on carbonyl-bridged TPAs (TN-(CO)-Pol) which was used as a cathode material.^23^ The polymer was obtained *via* carbonyl-based condensation polymerization mediated by Lawesson's reagent. The rigid carbonyl moieties lead to a two-dimensional (2D) polymer with a nanosheet layered morphology. A specific capacity of 340 mA h g^−1^ and a capacity retention of 95% after 200 cycles were reported for TN-(CO)-Pol. Müllen and coworkers successfully polymerized dimethylmethylene-bridged *N*-HTA under Yamamoto conditions.^24^ The polymer TN-(Me)-Pol displayed amorphous morphology and high air stability leading to a robust performance as a p-type semiconductor in OFETs. Nevertheless, TN-(Me)-Pol was not suitable as cathode material owing to its solubility in common organic solvents and poorly resolved, partially irreversible redox events. Nevertheless, to the best of our knowledge, no further studies of *N*-HTA-based polymers as organic cathode materials in lithium-ion batteries have been reported to date.

Our group has recently established a new family of *N*-HTAs comprising sterically demanding C(sp^3^)-based spirofluorene bridging units (denoted as FTNs). In these compounds, the sterically shielded environment together with the structural constraint around the nitrogen center significantly contribute to the stabilization of the corresponding radical cation which was isolated and characterized by X-ray crystallography.^25^ Moreover, as a result of the pronounced conformational rigidity of these scaffolds, interesting aggregation behavior leading to different morphologies with strongly altered photophysical properties was observed.^26^ Motivated by these findings, we aimed herein to exploit the potential of FTNs as monomers for the fabrication of redox-active porous polymers for application as organic cathode materials in lithium-ion batteries. Specifically, we have engineered brominated FTN monomers and subjected them to nickel-mediated Yamamoto polymerization to achieve well-defined polymers with distinct porosity and morphology defined by the substitution pattern of the monomer. The resulting porous polymers are thermally robust with decomposition temperatures above 400 °C and Brunauer–Emmett–Teller (BET) surface areas up to 690 m^2^ g^−1^. The reversible redox activity of the FTN moiety is clearly retained in the polymer, which is reversibly oxidized at +3.81 V (*vs.* Li/Li^+^) in composite electrodes with carbon black. In long-time galvanostatic charge/discharge measurements specific capacities up to 26 mA h g^−1^ were achieved, whereby excellent cycling stabilities with 80% retention of the initial specific capacity after 400 cycles was observed. The polymer is fluorescent and exhibits remarkable solvatochromism when dispersed in solvents of different polarities.

## Results and discussion

### Synthesis of the monomers

**Scheme 1:**
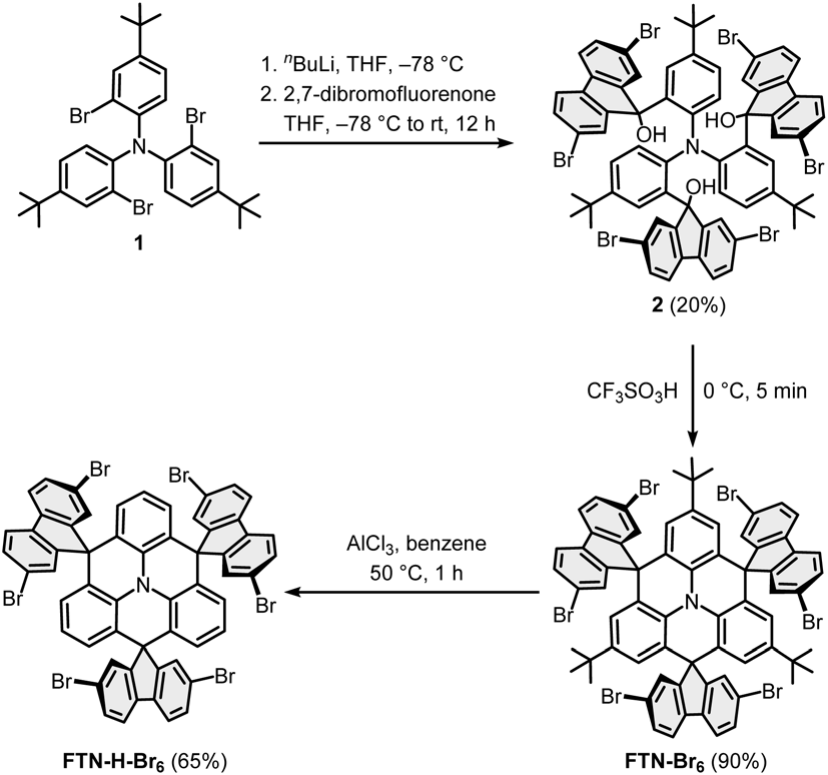
Synthetic route towards the monomers FTN-Br_6_ and FTN-H-Br_6_.

The synthesis of the monomers FTN-Br_6_ and FTN-H-Br_6_ is shown in Scheme 1a.^25,27^ The brominated building blocks were synthesized from compound 1, which is readily accessible on a multi-gram scale.^28^ Lithium/bromine exchange with *n*-BuLi at −78 °C and subsequent addition of 2,7-dibromofluorenone afforded the triol 2 in a yield of 20%. The rather low yield was already observed in the synthesis of parent FTN and originates most likely from the sterically overcrowded nature of the substituted triphenylamine.^25^ Threefold cyclization with trifluoromethanesulfonic acid at 0 °C delivered FTN-Br_6_ in a yield of 90%. The *tert*-butyl groups were selectively removed *via* a retro-Friedel–Crafts alkylation with AlCl_3_ in benzene at 50 °C to achieve FTN-H-Br_6_ in 65% yield.^27^

### Synthesis of the polymers

**Scheme 2:**
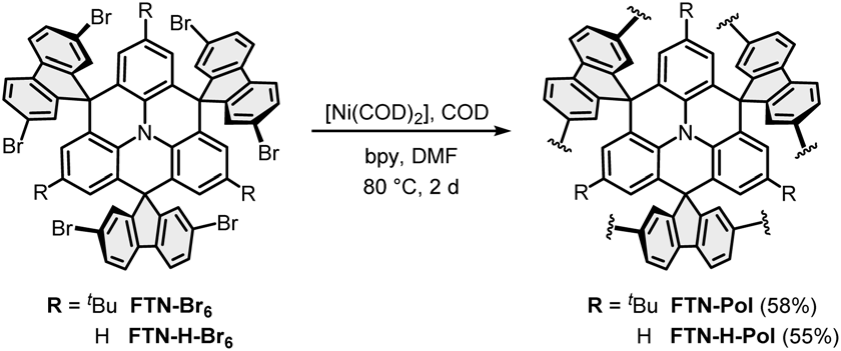
Synthesis of the polymers FTN-Pol and FTN-H-Pol under Yamamoto-reaction conditions.

FTN-Pol and FTN-H-Pol were synthesized *via* the Yamamoto polymerization using the standard protocol.^29^ The monomer was added in one portion to the activated catalyst mixture of [Ni(COD)_2_], 1,5-cyclooctadiene (COD), and 2,2′-bipyridine (bpy) in *N*,*N*-dimethylformamide (DMF; Scheme 2). The polymers FTN-Pol and FTN-H-Pol were obtained as pale yellow to dark orange insoluble powders and were further purified by Soxhlet extraction with THF. Drying at 100 °C for 12 h delivered FTN-Pol and FTN-H-Pol in comparable yields of 58% and 55%, respectively. Both polymers are insoluble in common organic solvents like methanol, CH_2_Cl_2_, chloroform, ethyl acetate, THF, or toluene.

### Photoluminescence properties of FTN-Pol

**Fig. 2:**
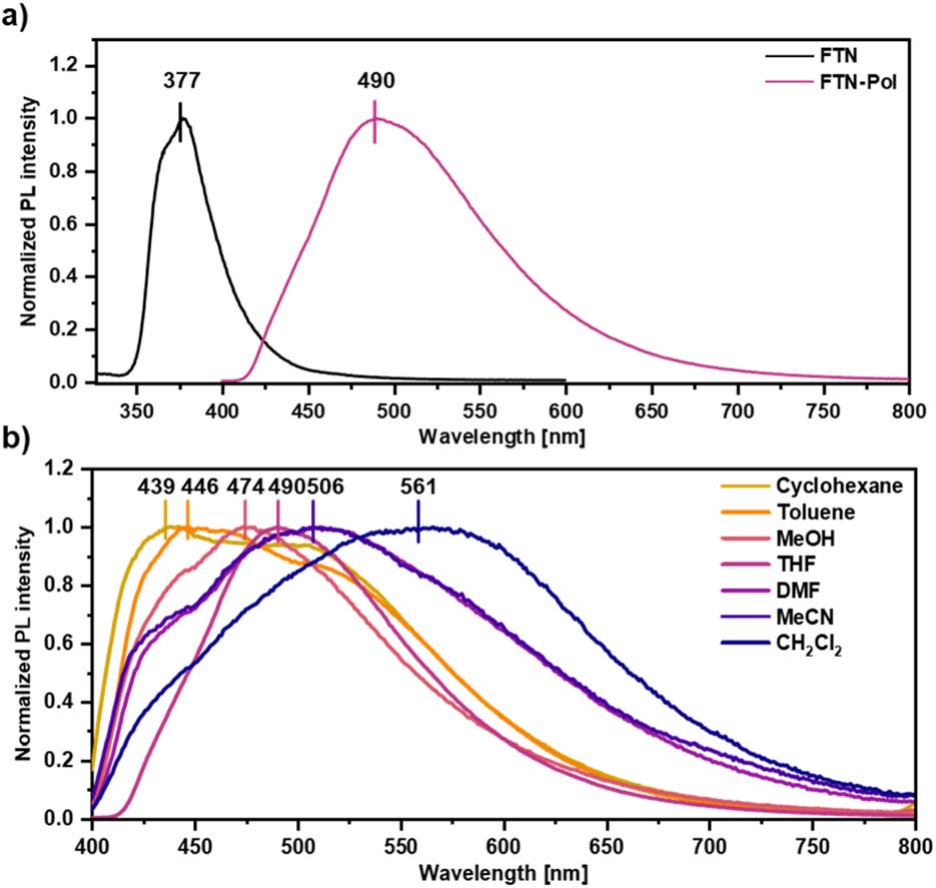
(a) Normalized PL spectra of FTN and FTN-Pol in THF at room temperature (*λ*_exc_ = 310 nm). (b) Normalized PL spectra of FTN-Pol dispersed in different organic solvents at room temperature (*λ*_exc_ = 370 nm).

The effect of polymerization on the photoluminescence properties was studied by emission spectroscopy and the obtained spectra were compared to those of the molecularly dissolved model compound FTN (for synthetic details, see ESI†). While the emission spectrum of FTN displays a single maximum at 377 nm (3.29 eV) and a shoulder at 368 nm (3.37 eV) accompanied by a tail reaching to 500 nm (Fig. 2a), FTN-Pol dispersed in THF (upon ultrasonication at room temperature for 30 min) shows a broad bathochromically shifted emission maximum at 490 nm (2.53 eV) reaching to 750 nm. The influence of solvent polarity on the emission properties was investigated in stable dispersions of FTN-Pol in cyclohexane, toluene, methanol (MeOH), tetrahydrofuran (THF), *N*,*N*-dimethylformamide (DMF), acetonitrile (MeCN), and CH_2_Cl_2_ (Fig. 2b; for the characterization of the dispersed aggregates by Raman spectroscopy and scanning electron microscopy (SEM), see ESI†). In all studied solvents, a broad emission extending from *ca.* 400 nm to at least 700 nm with two emission features at ≈430 nm and ≈500 nm was observed. The relative intensity of these features changes with the solvent polarity, with more polar solvents leading to red-shifted emission. The PL quantum yields (PLQY) were determined by using an integrating sphere and were in the range between 0.01 and 0.03, which is in accordance with the PLQY of the model compound FTN (*Φ* = 0.04, Table S3 in ESI†).^26^ Dispersions in less polar solvents like cyclohexane (*Φ* = 0.02), toluene, or THF (*Φ* = 0.03) display somewhat higher PLQY compared to those observed in dispersions in more polar solvents like MeOH, MeCN, DMF, or CH_2_Cl_2_ (*Φ* = 0.01). Further discussion of the observed solvent effects can be found in ESI.†

### Polymer characterization

**Fig. 3:**
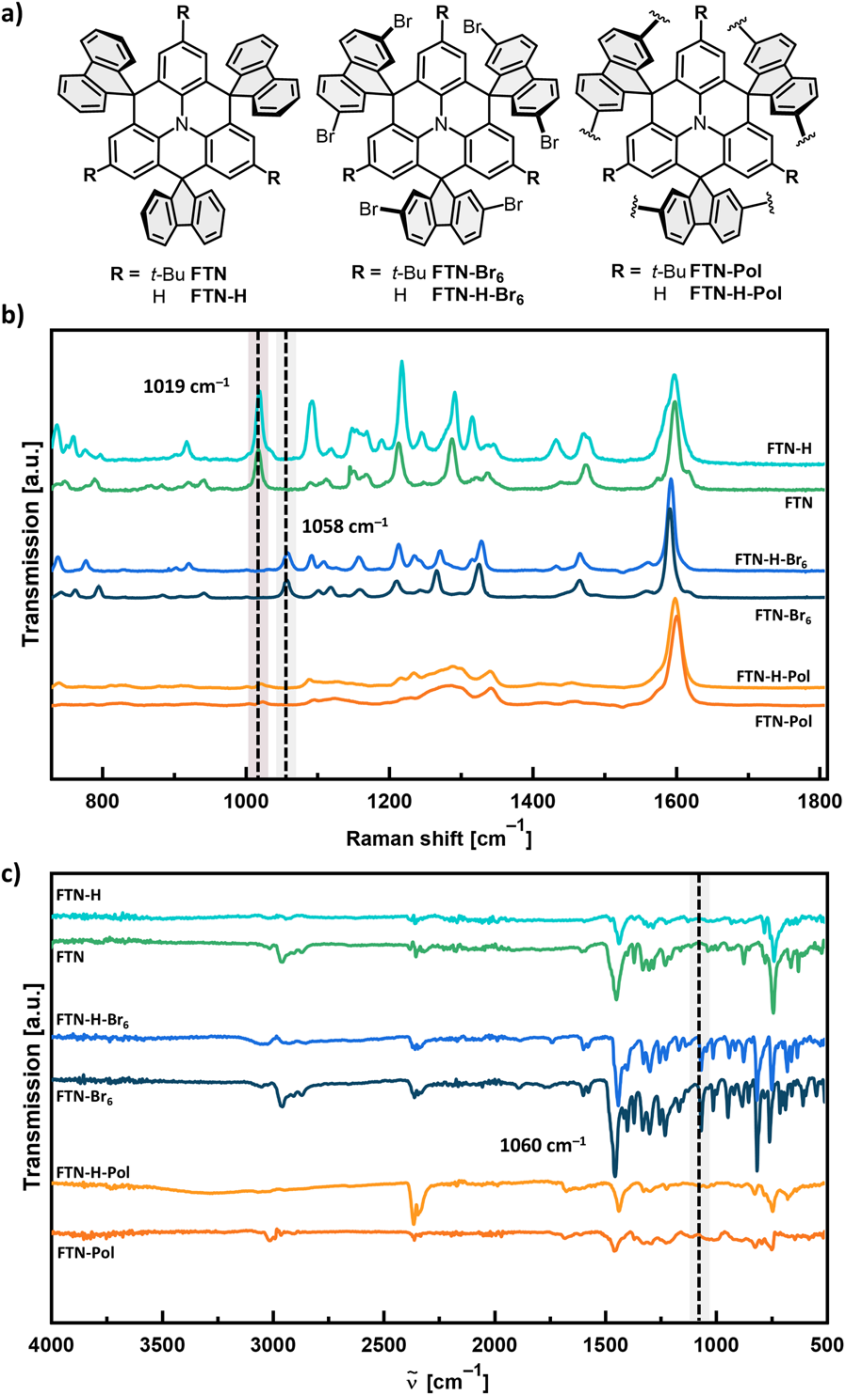
(a) Chemical structures of the model compounds FTN and FTN-H, the brominated monomers FTN-Br_6_ and FTN-H-Br_6_, and the polymers FTN-Pol and FTN-H-Pol. (b) Raman spectra of the compounds measured on glass substrates. (c) FT-IR spectra of the compounds measured as powders. The dashed line with red background corresponds to the C–H fluorenyl vibration bands, while the dashed lines with gray background correspond to the C–Br vibration modes.

Elemental combustion analysis confirmed the general chemical identity and elemental composition of purified FTN-Pol and FTN-H-Pol, while time-of-flight secondary mass spectrometry (ToF-SIMS) indicated no remaining nickel species (see Fig. S42 and S43 in ESI†). Powder X-ray diffractometry (PXRD) indicated the amorphous character of both polymers (Fig. S22 in ESI†). The polymers were further characterized by Raman and FT-IR spectroscopy to investigate the presence of the remnant bromo end groups. By comparing the Raman spectra of FTN-Pol and FTN-H-Pol with the corresponding monomers FTN-Br_6_ and FTN-H-Br_6_, the absence of the C–Br vibration modes at ≈1058 cm^−1^ indicates the complete conversion of the bromo moieties into new bonds (Fig. 3a and b). In addition, the C–H fluorenyl-vibration bands at ≈1019 cm^−1^ were identified in low intensities in both polymers, indicating the complete conversion of the bromo moieties to C–H bonds at the end sites of the polymer network. The identity of these bands was confirmed by comparison with the spectral signatures of the dehalogenated model compounds FTN and FTN-H (for synthetic details, see ESI†). FT-IR spectroscopy further corroborated the successful polymerization by the absence of C–Br stretching peaks at ≈1060 cm^−1^ for both polymers (Fig. 3c).

Thermogravimetric analysis (TGA) indicated an impressively high thermal stability up to 500 °C for FTN-Pol and even up to 700 °C for FTN-H-Pol (see Fig. S19–S21 in ESI†). The initial low weight loss is most likely related to adsorbed moisture and gases. The further weight loss of ≈20% observed in the range of 400–600 °C for FTN-Pol presumably corresponds to the removal of the *tert*-butyl side groups. First above 700 °C both polymers exhibit a dramatic weight loss which is clearly attributed to degradation of the polymer backbone.

### Morphological properties

**Fig. 4:**
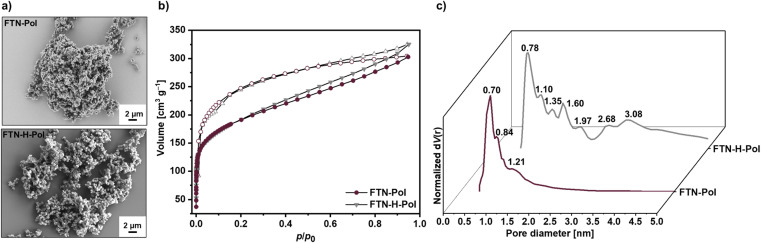
Morphological and porosity characterization of FTN-Pol and FTN-H-Pol. (a) SEM images measured as powders. (b) Adsorption and desorption isotherm (N_2_, 77 K). (c) Pore size distribution estimated by NL-DFT calculations.

The morphology of FTN-Pol and FTN-H-Pol was further studied by SEM. Both polymers appear mostly amorphous and consist of globular-like particles with coralloid surfaces which agglomerate to larger structures (Fig. 4a, S38 and S39 in ESI†). Nitrogen sorption analysis at 77 K after activation at 100 °C for 3 h was used to characterize the porosity of FTN-Pol and FTN-H-Pol (Fig. 4b). In both cases, the nitrogen adsorption resulted in type I isotherms, which indicates networks with microporous character.^30^ Furthermore, hysteresis between the adsorption and desorption isotherms was observed, suggesting that the adsorption of nitrogen molecules causes an elastic deformation or swelling of the covalent network.^31^ The specific BET^32^ surface areas are comparable for both polymers and amount to 690 m^2^ g^−1^ and 682 m^2^ g^−1^ for FTN-Pol and FTN-H-Pol, respectively (Fig. S45–S50 in ESI†). The pore size distribution curves were determined using the non-local density functional theory (NL-DFT)^33^ calculations and revealed for FTN-Pol micropores with a pore diameter of 0.70 nm (Fig. 4c). Larger pores with a diameter of 0.84 and 1.21 nm were also observed. In contrast, the pore size distribution curve of FTN-H-Pol shows a slightly increased pore diameter of 0.78 nm and a broad pore size distribution between 1.10 and 3.08 nm. The broader pore size distribution of FTN-H-Pol was attributed to the structural difference between the spirofluorene-bridged monomers FTN and FTN-H. In the case of FTN, the solubilizing *tert*-butyl groups provide for a better control during the irreversible Yamamoto polymerization. Thereby, the sterically demanding *tert*-butyl groups not only protect the reactive *para*-positions of the *N*-HTA core, but also exert a considerable directing effect during the polymerization.^25^ Hence, exclusively the electrochemical properties of the structurally better defined FTN-Pol were investigated.

### Electrochemical properties and battery studies

**Scheme 3:**
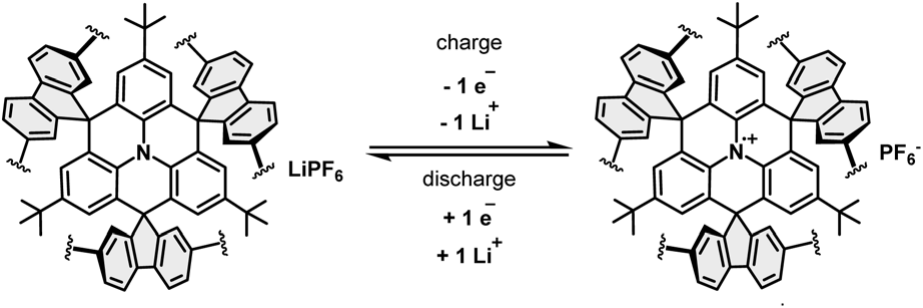
Redox reaction of FTN-Pol associated with the theoretical specific capacity of *ca.* 30 mA h g^−1^.

Scheme 3 depicts the electrochemical process which is expected in FTN-Pol upon cycling as cathode material in lithium-ion batteries. FTN-Pol has one nitrogen-active redox center per monomer. The positively charged polymer is stabilized by PF_6_^−^ counter anions of the electrolyte solution and is able to accept one electron per monomer after reduction. In the reduction process, lithium cations migrate towards the PF_6_^−^ ions to reach again a neutral state. The theoretical specific charge capacity of our redox-active polymer FTN-Pol associated with this single process is 29.97 mA h g^−1^, whereby every *N*-HTA unit is able to donate one electron (*n* = 1) in the oxidation process.

**Fig. 5:**
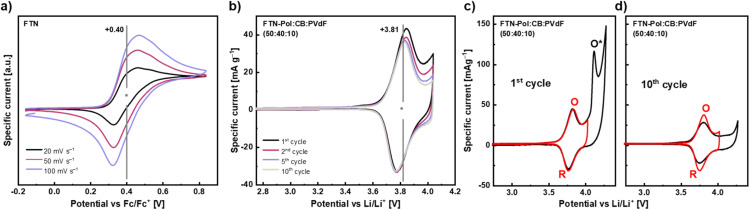
(a) CV of FTN in CH_2_Cl_2_ with 0.1 M *n*-Bu_4_NPF_6_ at different scan rates. (b)–(d) CVs of FTN-Pol based composite electrodes (50 wt%) with 1 M LiPF_6_ and a scan rate of 0.1 mV s^−1^ for different voltage ranges. Specific currents are normalized to the mass of FTN-Pol. Note, the production of electrodes with 30 wt% CB and 60 wt% FTN-Pol yielded similar results as the here presented electrodes (for details, see ESI†). O = oxidation peak at +3.85 V, O* = oxidation peak at +4.15 V, R = reduction peak at +3.78 V.

To investigate the electrochemical properties of the model compound FTN, cyclic voltammetry (CV) studies in CH_2_Cl_2_ at room temperature with 1 M *n*-Bu_4_NPF_6_ as an inert supporting electrolyte were conducted (Fig. 5a). FTN displays a fully reversible one-electron oxidation at +0.40 V (*vs.* ferrocene/ferrocenium (Fc/Fc^+^) redox couple), which is independent of the scan rate and corresponds to the formation of a nitrogen-centered radical cation.^25^ In contrast to parent TPA, which is oxidized at +0.53 V, the observed anodic shift of 130 mV points towards efficient stabilization of the positive charge through delocalization within the planarized *N*-HTA core.^21^ The electrochemical features of FTN, especially its narrow and well-defined oxidation towards the stabilized nitrogen-centered radical cation, prompted us to investigate FTN-Pol as a positive electrode material in lithium-ion batteries. Composite electrodes containing FTN-Pol (50 wt%), carbon black (CB) (40 wt%) as conductive additive, and polyvinylidene fluoride (PVdF) binder (10 wt%) were fabricated (for details, see ESI;† CB is electrochemically inactive in this voltage regime). For electrochemical studies, a coin-cell setup was used, where 1 M LiPF_6_ in ethylene carbonate/dimethyl carbonate (EC/DMC; 1/1 vol%) was used as electrolyte, while metallic lithium served as counter and reference electrode.^34^ CV measurements in a voltage range between +2.5 and +4.0 V *vs.* Li/Li^+^ and at a scan rate of 0.1 mV s^−1^ revealed an oxidation peak (O) at +3.85 V and a reduction peak (R) at +3.78 V (Fig. 5b and c), as well as a shoulder like feature at +4.0 V.

Similar to the CV data recorded in solution for the model compound FTN, precise and very sharp peaks were measured in battery CVs for FTN-Pol, indicating that the polymerization did not affect the unique electrochemical properties of FTN. The intensity of the redox peaks barely decreases after the 2nd cycle. This low intensity decrease could imply that the typical degradation effects of organic battery materials, such as for example dissolution of the active material into the battery electrolyte, is efficiently circumvented by our architecture. This is further supported by the given symmetry between the anodic and cathodic half-waves, which indicates that both redox processes are completely reversible. By extending the scanning regime up to +4.3 V and thereby further investigating the shoulder like feature, a clear additional oxidation peak (O*) at +4.15 V arises (Fig. 5c). However, the complementary reduction feature is not visible and also O* disappears in the 2nd cycle (Fig. S52–S57 in ESI†). The effect on the main redox couple O/R through increasing the voltage range and thereby including O* is mostly that O/R fades faster.

**Fig. 6:**
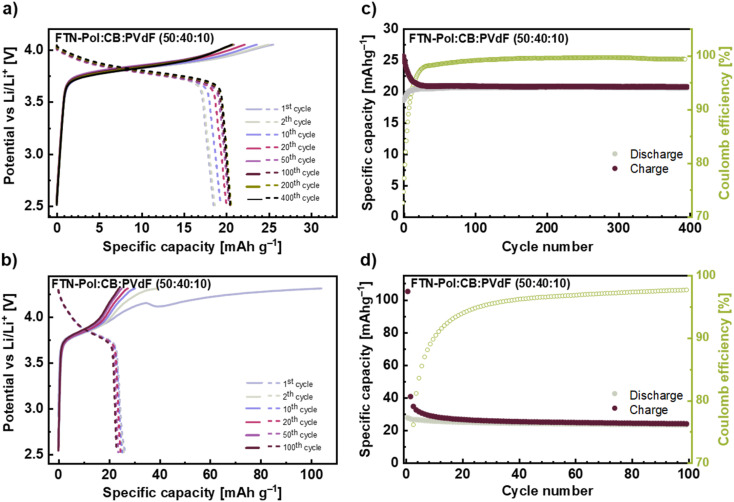
(a) and (b) Galvanostatic charge and discharge profiles of selected cycles with a current density of 100 mA h g^−1^ for different voltage ranges +2.5 to +4.0 V and +2.5 to +4.3 V. (c) and (d) Constant current cyclings at 100 mA h g^−1^ for 100 and 400 cycles. Normalisation is with respect to active material FTN and FTN-Pol. In (c) and (d), capacity of CB was subtracted.

Long-time (100 and 400 cycles) galvanostatic charge/discharge measurements explored at a current density of 100 mA g^−1^ (*i.e.*, roughly 3C) in the range between +2.5 and +4.0 V for the FTN-Pol composite electrode are shown in Fig. 6a and c. The initial charge capacity of 26 mA h g^−1^ (87% of the theoretical charge capacity) declined to a constant charge capacity of 21 mA h g^−1^ after the 20th cycle resulting in a capacity retention of 81%. As can be seen from Fig. 6a, the potential lines of different cycles nearly perfectly overlap until +3.95 V and after the 20th cycle, there is a negligible difference between them in the entire voltage range. The Coulomb efficiency (CE) of the FTN-Pol-electrode in the restricted voltage regime improves continually during cycling and reaches a stable value of above 97% after 25 cycles indicating a highly reversible redox reaction at the nitrogen atom (Fig. 6c). The electrochemical performance of FTN-Pol is not only influenced by the *N*-HTA redox unit, but also by the microporous nature of the polymer. Especially the high surface area and the well-distributed pore sizes play a critical role in enhancing lithium-ion storage and diffusion, contributing to the observed specific capacities and cycling stability.^35^ The microporous structure of FTN-Pol provides a high surface area, facilitating the interaction between the electrode and the electrolyte. This interaction increases the number of active sites available for the redox reaction, leading to higher specific capacities.^36^ Additionally, the porosity of FTN-Pol promotes efficient ion diffusion throughout the polymer matrix, which improves the rate performance and lowers the resistance during charge/discharge cycles.^37^ The structural stability of FTN-Pol originating from the rigid spiro junctions, further contributes to the durability and long-term performance of the battery.^38^

In order to assess the effect of the process O*, we also performed the measurements in the extended voltage regime +2.5 to +4.3 V, which includes the process O* in the first half-cycle (Fig. 6b and d). The measurements in the extended voltage regime show a much greater initial capacity of 105 mA h g^−1^ and a rapid charge capacity drop to 40 mA h g^−1^ already in the 2nd cycle. Thereafter, a much slower decrease for the next 20 cycles is visible until an almost constant value of 25 mA h g^−1^ is reached and maintained to cycle 100. The rapid drop in capacity between 1st and 2nd cycle further underscores the irreversibility of the process O*, which has been discussed above. The measurements in the extended voltage regime revealed that CE reaches a value of 97% after 75 cycles which indicates the above mentioned strong irreversible character of the reactions O* between +4.0 and +4.3 V (Fig. 6d). From the observed behavior we conclude that the long-term reversible capacity is dominated by the process O/R. The slightly higher capacity value as compared to the one obtained when cycling to +4.0 V corresponds to the open CV at +4.0 to +4.3 V.

**Fig. 7:**
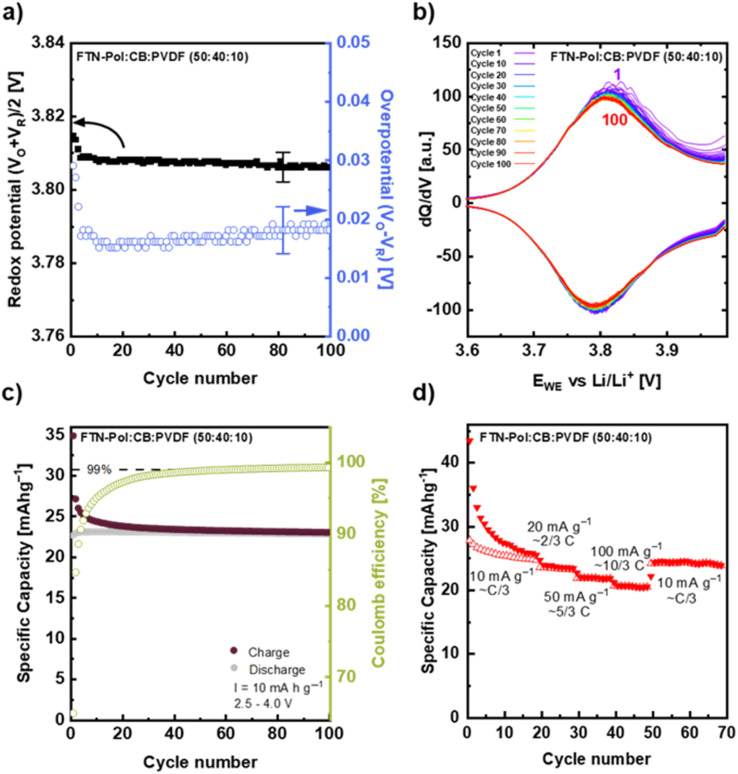
Cycling studies conducted at a reduced current density of 10 mA h g^−1^. (a) Redox potential (V_O_ + V_R_)/2 and overpotential (V_O_ − V_R_) and (b) selected derivatives of galvanostatic charge/discharge measurements with a current density of 10 mA h g^−1^ in the voltage range of +2.5 to +4.0 V for 100 cycles. (c) Specific capacity obtained at 10 mA h g^−1^ for 100 cycles. Normalisation is with respect to active material FTN-Pol. (d) Rate performance measurement at currents rates of about C/3, 2/3 C, 5/3 C, and 10/3 C.

Fig. 7 shows the cycling stability of FTN-Pol upon cycling at the reduced current density of 10 mA g⁻^1^. The redox potential, calculated as the mean of the oxidation peak potential (V_O_), and the reduction peak potential (V_R_), along with the overpotential (V_O_ − V_R_), are depicted in Fig. 7a. V_O_ and V_R_ were obtained from the derivatives of the charge/discharge profiles measured in the voltage range of +2.5 to +4.0 V at the current density of 10 mA g⁻^1^ (approximately C/3; Fig. 7b). The redox potential remains constant within error bars at +3.807 ± 0.005 V. After a sharp drop during the initial 10 cycles, the overpotential does not change either and amounts to 0.017 ± 0.005 V. The fact that both the redox potential and the overpotential are constant indicates a stable reaction mechanism at C/3 which in particular shows better stability when compared to cycling at about 3 C (see Fig. S58a in ESI†).

The corresponding derivatives of the galvanostatic charge/discharge profiles at the same current density of 10 mA g⁻^1^ over 100 cycles shown in Fig. 7b confirm the stability of the electrochemical process. The oxidation/reduction peak potentials and the intensity of the redox peaks exhibit minimal decrease after the 3rd cycle but do not change upon further cycling. The only slight initial reduction may indicate that the side reaction at +4.0 V is less significant compared to the cycling studies at ∼3 C (Fig. S58b in ESI†). The data also demonstrate that degradation mechanisms typically associated with organic battery materials, such as active material dissolution into the electrolyte, are effectively mitigated by our polymer design.^39^ This interpretation is further substantiated by the observed symmetry between the anodic and cathodic half-waves, indicating entirely reversible redox processes after the 20th cycle. Long-term galvanostatic charge/discharge measurements of the FTN-Pol composite electrode at 10 mA g⁻^1^ (∼C/3) within the voltage range of +2.5 to +4.0 V show an initial charge capacity of 35 mA h g⁻^1^ which decreases to a stable value of 24 mA h g⁻^1^ (80% of the theoretical charge capacity) after 20 cycles (Fig. 7c). Similar to the measurements at 3 C, CE of the FTN-Pol electrode improves continuously during cycling at C/3 and reaches the same value of 99.1% after 100 cycles. This value is however reached faster at ∼C/3 than at ∼3 C which again points to a side reaction causing initially reduced CE (Fig. S58c in ESI†).

In Fig. 7d the rate capability performance is shown. During the first 20 cycles, a current density of 10 mA g⁻^1^ was applied, which approximately corresponds to C/3. The initial charge capacity of 43 mA h g⁻^1^ decreases to a steady-state capacity of 25 mA h g⁻^1^, corresponding to 83% of the theoretical capacity. After the first 20 cycles it yields a reversible specific capacity of 23, 22, and 21 mA h g^−1^, at current rates of ∼2/3 C, ∼5/3 C, and ∼10/3 C, respectively. When the current density is reduced back to ∼C/3 in cycle 50, the specific capacity returns to 24 mA h g^−1^, demonstrating the good reversibility of the FTN-Pol-based electrodes. While the current density does not have a great impact on the capacity, the main effect are significant changes in the initial 20 cycles (depending on the actual current density) which we attribute to the aforementioned side reaction.

Notably, FTN-Pol is the first cathode material based on *N*-HTA, where the stable redox process at the nitrogen center has been thoroughly investigated. By implementation of a well-defined porosity provided by the rigid spirocyclic architecture, we have developed a unique redox-active polymer with high cycling stability and CE. The role of microporosity in battery performance was previously examined for the triphenylamine-based polymer TPA-Pol, demonstrating that increased porosity leads to larger surface areas and, consequently, improved battery performance (for more details see Table S4 in ESI†).^40^ Compared to TPA-Pol, our FTN-Pol exhibits a redox process in a similar voltage range (∼3.8 V *vs.* Li/Li^+^). However, FTN-Pol shows a narrower and more defined CV, suggesting that the π-system of the *N*-HTA stabilizes the nitrogen-centered oxidation. Despite the lower specific capacity of FTN-Pol compared to TPA-Pol, due to the inherently high molecular weight of the spiro-bridged *N*-HTA redox units, FTN-Pol achieves 87% of its theoretical capacity, surpassing the performance of TPA-Pol, which reaches only 81%. Moreover, the cycling stability of FTN-Pol is comparable to TPA-Pol, with FTN-Pol achieving a CE of 99.6% after 400 cycles, while TPA-Pol reaches a CE of 99.3% after 1000 cycles. The rate performance is also comparable between the two polymers. For FTN-Pol, the steady-state capacity decreases from 21.68 mA h g⁻^1^ to 20.35 mA h g⁻^1^ as the current density increases from 50 mA g⁻^1^ to 100 mA g⁻^1^, while TPA-Pol shows a capacity change from 77.11 mA h g⁻^1^ to 69.96 mA h g⁻^1^ under the same conditions (for more details, see Table S4 in ESI†).

## Conclusion

We have fabricated novel microporous polymers upon Yamamoto polymerization of selectively brominated spirofluorene-bridged *N*-heterotriangulenes, which were readily accessible through modular synthetic routes. Combined characterization by combustion analysis, ToF-SIMS, IR, and Raman spectroscopy unambiguously confirmed the elemental composition and the absence of the catalyst and the reactive terminal groups in the purified polymer. As revealed by powder X-ray diffractometry and scanning electron microscopy, the polymers are amorphous and occur as globular particles which aggregate into larger structures. The polymers show remarkable thermal stability with decomposition temperatures above 400 °C and BET surface areas up to 690 m^2^ g^−1^. However, the polymer decorated with the *tert*-butyl groups (FTN-Pol) exhibited a considerably narrower pore size distribution and was used for further studies. Compared to monomeric FTN, the polymer exhibits bathochromically shifted emission when dispersed in different solvents, which is highly sensitive to the solvent polarity. The reversible redox activity of the FTN moiety is retained in the polymer, which is reversibly oxidized at +3.81 V (*vs.* Li/Li^+^) in composite electrodes with carbon black. Specific capacities up to 26 mA h g^−1^ with excellent cycling stability with 80% retention of the initial specific capacity after 400 cycles were achieved in the lithium-ion batteries with FTN-Pol used as cathode material.

Our findings demonstrate the potential of the spirofluorene-bridged *N*-heterotriangulenes for the development of functional redox-active porous polymers. With the established modular synthesis of the required monomers in hand we believe that polymers with significantly increased porosity and specific capacity will be in reach upon implementation of designer linking units and additional redox-active moieties.

## Author contributions

A. Jocic synthesized and characterized all compounds and polymers, contributed to the data analysis wrote the original manuscript draft. T. Wickenhäuser performed the battery studies and together with R. Klingeler analyzed the data. S. Lindenthal did the photoluminescence measurements and together with J. Zaumseil analyzed the data. W.-S. Zhang characterized the polymers by electron microscopy and together with R. Schröder analyzed the data and prepared the images. M. Kivala conceived and supervised the project, acquired the funding, edited, and finalized the manuscript. All authors discussed the results and commented on the manuscript.

## Conflicts of interest

There are no conflicts to declare.

## Supplementary Material

SC-015-D4SC04276J-s001

## Data Availability

Experimental details and characterization data as well as additional spectroscopic and electrochemical data and electron microscopy images can be found in the ESI.†
