# Supplementary material for: Redox-active, photoluminescent porous polymers based on spirofluorene-bridged N-heterotriangulenes and their feasibility as organic cathode materials
Source: Chem Sci. 2024 Oct 23;15(45):19094–103. doi: 10.1039/d4sc04276j (PMC11523812; doi:10.1039/d4sc04276j)
Supplement: SC-015-D4SC04276J-s001 [file SC-015-D4SC04276J-s001.pdf]

Supplementary Information (SI) for Chemical Science.
This journal is © The Royal Society of Chemistry 2024

Electronic Supplementary Information

Redox-active, photoluminescent porous polymers based on spirofluorene-bridged *N*-heterotriangulenes and their feasibility as organic cathode materials

Angelina Jocic, Tom Wickenhäuser, Sebastian Lindenthal, Wen-Shan Zhang, Jana Zaumseil, Rasmus Schröder, Rüdiger Klingeler, Milan Kivala*

Table of Contents

1. Methods and Instrumentation 3

2. Synthetic Protocols 7

3. Nuclear Magnetic Resonance Spectra 14

4. Infrared Spectra 18

5. Raman Spectra 19

6. TGA and DSC Measurements 20

7. Powder X-Ray Diffraction 25

8. UV/Vis Absorption and Fluorescence Spectroscopy 25

9. Fluorescence Measurements of FTN and FTN-Pol Dispersions 28

10. SEM Images 33

11. Time-of-Flight Secondary Mass Spectrometric Analysis 37

12. Gas Sorption Analysis 38

13. Electrochemistry Measurements 41

14. Electrode Data 42

15. References 45

1. Methods and Instrumentation

1.1 General Remarks.

Solvents and reagents were purchased at reagent grade from commercial suppliers like Merck/Sigma-Aldrich, TCI, Thermo Fisher Scientific, Acros Organics, and Honeywell and were used without further purification. All reactions requiring exclusion of oxygen and moisture were carried out in dry glassware under a dry and oxygen-free nitrogen atmosphere using standard Schlenk techniques. Dry solvents were obtained from the solvent purification system MBraun MB-SPS-800 or purchased from Fisher Scientific. Dry THF was purified via distillation over sodium metal. All solvents were stored over molecular sieves (3 Å or 4 Å). Flash column chromatography was carried out on silica gel (MN Silica Gel 60A, 230–400 mesh). Analytical TLC was performed on aluminum plates coated with 0.20 mm silica gel or 0.20 mm Al_2O_3 and a fluorescent indicator (Macherey-Nagel, ALUGRAM®, SIL G/U30V_{254}). Components were visualized by observation under UV irradiation (λ = 254 nm and 366 nm). Compounds **1**, **S1,** and **FTN** were prepared according to literature procedures.[1] Compounds **FTN-H**, **FTN-Br$_6$**, and **FTN-H-Br$_6$** were synthesized according to our published protocol.[2]

1.2 Instrumentation

Melting points (M.p.) were determined in open glass capillaries with a Melting Point Apparatus Büchi M-560.

^{1}H NMR and ^{13}C NMR spectra were recorded in $CDCl_3$, CD_2Cl_2, or THF-d_8 at room temperature on a Bruker Avance III 600 (600 MHz for ^{1}H and 151 MHz for ^{13}C). Chemical shifts (δ) are reported in parts per million (ppm) and were referenced to the residual solvent signal as an internal reference ($CDCl_3$: 7.26 ppm for ^{1}H, 77.16 ppm for ^{13}C; CD_2Cl_2: 5.32 ppm for ^{1}H, 53.84 ppm for ^{13}C, THF-d_8: 1.72 ppm for ^{1}H, 67.21 ppm for ^{13}C).[3] Coupling constants (*J*) are given in Hz and the apparent resonance multiplicity is reported as s (singlet), d (doublet), t (triplet), or m (multiplet).

Mass spectrometry analysis was measured on a BRUKER Autoflex Speed MALDI-TOF (Bruker, MALDI).

Infrared spectra were recorded on a Jasco FT/IR-4600 (ATR mode) spectrometer. Characteristic IR bands were categorized as strong (s), medium (m), and weak (w) are reported in cm^{-1}.

UV/Vis absorption spectra were acquired on a Varian Cary 60 UV/Vis spectrophotometer with quartz cuvettes of 10 mm path length (QS Quartz Suprasil cells; Hellma Analytics). All compounds were measured in CH_2Cl_2 in a wavelength region of 230–800 nm and under ambient conditions.

Fluorescence spectra were recorded on a Jasco spectrofluorometer FP-8500 equipped with an ILF-835|100 mm integrating sphere. All measurements were performed in solution under ambient conditions. Quartz cuvettes of 10 mm pathlength

were used (Spectrosil Quartz cells; JASCO). The photoluminescence quantum yields (PLQYs) of the solutions and dispersions were determined using an integrating sphere and was calculated with Spectra Manager from JASCO.

Preparation of FTN-H-Pol dispersions was carried out by ultrasonication of the polymer in the respective solvent at room temperature for 30 min. The dispersed aggregates in each solvent were characterized by Raman spectroscopy (drop-casted on glass substrates) and SEM (dried dispersion). The comparison of the obtained data with the bulk material revealed that the characteristic features and the morphology remained intact after the dispersion process.

Fluorescence spectra of polymer dispersions (1 mg mL^{-1}) in different solvents were recorded with a Fluorolog 3 spectrofluorometer (Horiba Jobin-Yvon GmbH). For excitation, the output of a xenon lamp (450 W) was spectrally filtered by a double monochromator system (grating blaze 330 nm, 1200 lines mm^{-1}). Samples were placed in a SUPRASIL cuvette with a light path of 10 mm along the excitation path and 5 mm along the detection path. The emission from the samples was filtered through a long pass filter (405 nm, ThorLabs) and collected by a PPD-900 photomultiplier tube detector. In the case of PLE maps, spectra were corrected for the wavelength dependent lamp intensity.

Cyclic, differential, and square wave voltammetry (CV, DPV, and SWV) measurements were performed on a BASi Cell Stand with a three-electrode assembly of a glassy carbon disk working electrode, Ag/AgCl (3 M NaCl) quasi-reference electrode, and platinum wire auxiliary electrode. All measurements were carried out under nitrogen in an electrolyte solution of 0.1 M *n*-Bu_4NPF_6 in anhydrous CH_2Cl_2. The solution was purged with nitrogen for 15 min before every run. In addition, calibration with an internal standard (ferrocene/ferrocenium (Fc/Fc^+)) for the analysis was performed.

X-ray crystallographic data were acquired on a Stoe Stadivari diffractometer or Bruker APEX II Quazar diffractometer in the X-ray crystallography department of the Institute of Organic Chemistry, Heidelberg University. The structures were solved and refined with SHELXT-2014 and refined against F_2 with a full-matrix least-squares algorithm using the SHELXL-2018/3 software.[4] After full-matrix least-square refinement of the non-hydrogen atoms with anisotropic thermal parameters, the hydrogen atoms were placed in calculated positions using a riding model.

Elemental analysis was performed in the Microanalytic department (Institute of Organic Chemistry, Heidelberg University) on a vario MICRO cube device (Elementar).

IUPAC names of the compounds described in the experimental section were determined by ACD/Labs2021.

Thermogravimetric analysis (TGA) and differential scanning calorimetry (DSC) Were recorded on a METTELER Toledo TGA/DSC device. The measurements were carried out under a constant flow of nitrogen in a range between 30–800 °C with a heating rate of 10 K min^{-1}.

Raman spectra of drop-cast monomer and polymer films were acquired with a Renishaw inVia confocal Raman microscope in backscattering configuration equipped with a 50× long working distance objective (N.A. 0.5, Olympus) and an excitation wavelength of 785 nm. To minimize the influence of spot-to-spot variations, maps with >200 spectra were recorded and averaged.

Electrochemical battery measurements were performed at 25 °C on a VMP3 potentiostat from BioLogic. Working electrodes were produced by mixing active material (**FTN-Pol**), carbon black Working electrodes were produced by mixing active material (**FTN-Pol**), carbon black (CB), and polyvinylidene fluoride (PVDF) in N-methyl-2-pyrrolidinone (NMP). The slurry was stirred for 24 h before being spread onto a copper mesh current collector resulting in mass loadings of approximately 1-1.5 mg cm^{-2}. The electrodes were dried for 12 h in a vacuum oven at 80 °C and 10 mbar, pressed at 10 MPa, and dried again. In 2032 coin cells the working electrode, Glass fiber (Whatman GF/D) as separator, and pure lithium metal foil (Aldrich) as counter electrode were assembled. 1 M $LiPF_6$ in a mixture of ethylene carbonate (EC) and dimethyl carbonate (DMC) (1:1 vol.) was used as electrolyte. In the voltage range of 2.5 − 4.3 V vs. Li/Li^+ cyclic voltammetry measurements (CV) with a scan rate of 0.1 mV s^{-1} were performed as well as Galvanostatic cycle measurements with potential limitation (GCPL). For the normalization of the specific capacity and the current the mass of the active material (FTN-Pol) is taken into account. The theoretical capacity of **FTN-Pol** is calculated according to Faradays law, $Q_{theo} = n\cdot(F\cdot M_w^{-1})$ where n is the transferred electron number in each structural unit (n = 1), F the Faraday constant (F = 26801.5 mAh mol^{-1}) and M_w the molecular weight of the structural unit (M_w = 894.174 g mol^{-1})

Scanning electron microscopy and time-of-flight secondary ion mass spectrometry were conducted on a Crossbeam 540 (Zeiss Microscopy) FIB-SEM (Q150V ES Plus, Quantum Design), equipped with a fibTOF-Analyser (TOFWERK). SEM images were acquired with a 3 kV langing energy and a SESI-detector, while the SIMS measurements were carried out with a Ga-ion beam with a 30 keV landing energy. All samples were sputter coated with Pt/Pd (10nm).

Gas sorption measurements to analyze the surface area and pore diameters were recorded with a computer-controlled surface analyzer AUTOSORB-IQ3. The temperature was controlled by a digital thermometer and cooling was performed by a cooling bath with liquid nitrogen. The pressure was monitored by 10000, 10, and 0.1 torr sensors. For the sample preparation 10–20 mg of the polymers were loaded into 6 mm cryo cooler tubes and heated at 100 °C for 3 h in the vacuum degassing ports. The Brunauer-Emmet-Teller (BET) surface areas were calculated with the cross-sectional area value of 0.162 nm^2 for nitrogen molecules at 77 K. Pore size

distributions were determined via the nitrogen adsorption data using the non-local density functional theory (NL-DFT).[5]

Powder X-ray diffractometry (PXRD) was carried out on a Rigaku SmartLab diffractometer equipped with a capillary spin state (60 rpm) and a HyPix-3000 detector. The measurements were operated at 9 kW and an incident CuKα radiation at 1.54059 Å and a Debye-Scherrer geometry. All polymers were measured as powders in Mark-tubes containing special glass with an inner diameter of 0.6 mm (Hilgenberg). Baseline correction was performed by detecting the diffraction of an empty Mark-tube with the same conditions.

2. Synthetic Protocols

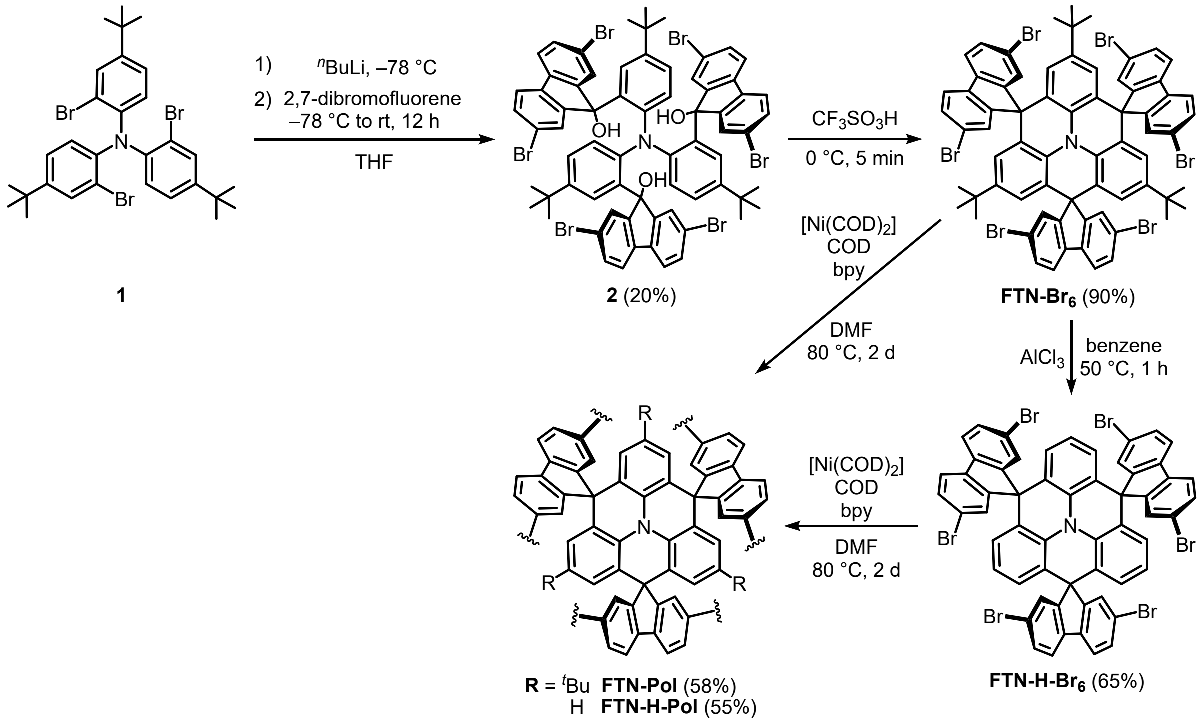

Scheme S1: Synthesis of the polymers **FTN-Pol** and **FTN-H-Pol**.[2]

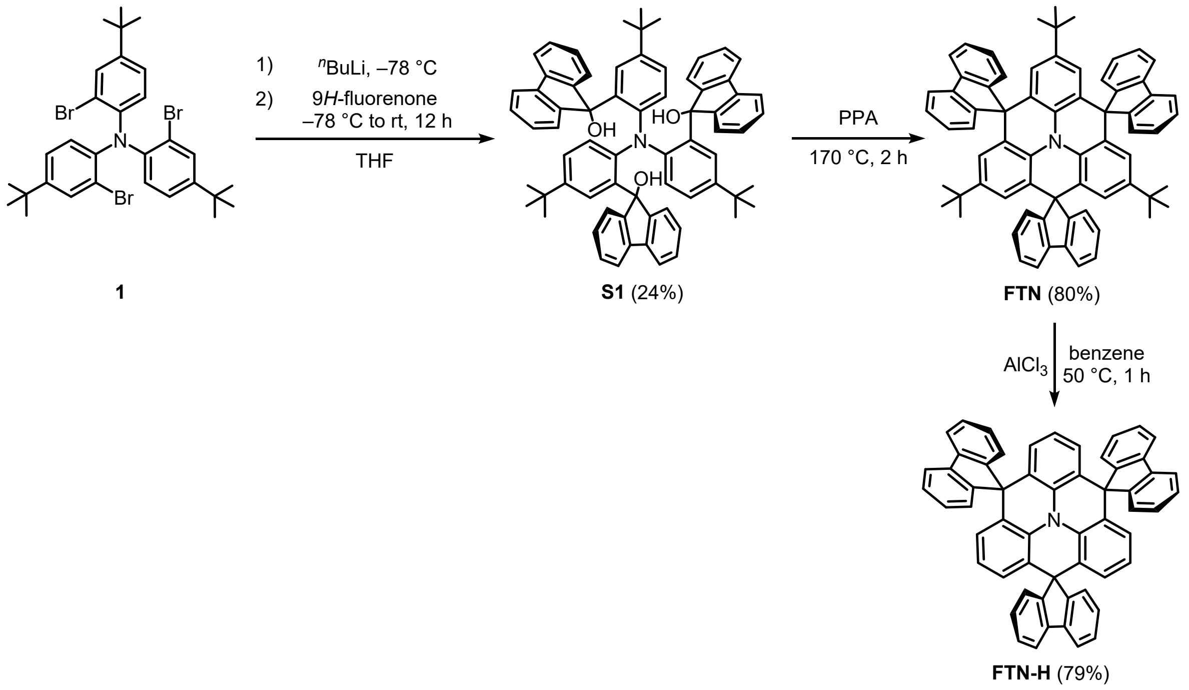

Scheme S2: Synthesis of the model compounds **FTN** and **FTN-H** starting from 2-bromo-*N,N*-bis(2-bromo-4-*tert*-butylphenyl)-4-*tert*-butylaniline (**1**).[1,2]

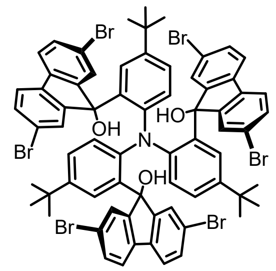

2

9,9',9''-Nitrilotris[5-(*tert*-2,1-phenylene)]tris(2,7-dibromo-9*H*-fluoren-9-ol) (2)

2-Bromo-*N,N*-bis(2-bromo-4-*tert*-butylphenyl)4-*tert*-butylaniline (**1**) (200 mg, 0.308 mmol) was dissolved in dry THF (2 mL) and cooled to –78 °C. *n*-BuLi in hexanes (1.6 M, 0.634 mL, 65.0 mg, 1.01 mmol) was added dropwise and the obtained yellow reaction mixture was stirred for 30 min. A solution of 2,7-dibromo-9*H*-fluoren-9-one (468 mg, 1.38 mmol) in dry THF (10 mL) was added dropwise and the reaction mixture was allowed to warm to room temperature overnight. After treating with sat. aq. NaCl (50 mL), the mixture was extracted with CH_2Cl_2 (3 × 400 mL). The organic phase was dried over Na_2SO_4, filtered, and the solvent was removed under reduced pressure. Purification by column chromatography (SiO_2, CH_2Cl_2 → EtOAc) gave a pale yellow solid which was recrystallized from hot CH_2Cl_2 overlayered with MeOH to obtain pure compound **2** (87.8 mg, 61.5 μmol, 20%) as a colorless solid.

M.p. (TGA): >400 °C (decomp.).

R_f = 0.43 (SiO_2, CH_2Cl_2).

^{1}H NMR (600 MHz, THF-d_8): δ 7.76 (d, *J* = 1.8 Hz, 3H), 7.68 (s, 3H),7.63 (d, *J* = 8.1 Hz 3H), 7.59 (d, *J* = 8.0 Hz, 3H), 7.44 (dd, *J* = 8.1 Hz,1.8 Hz, 3H), 7.39 (dd, *J* = 8.0 Hz, 1.8 Hz, 3H), 7.27 (dd, *J* = 8.6 Hz, 2.4 Hz, 3H), 6.84 (d, *J* = 8.6 Hz, 3H), 6.59 (d, *J* = 2.4 Hz, 3H), 6.45 (d, *J* = 1.7 Hz, 3H), 1.06 (s, 27H) ppm.

^{13}C NMR (151 MHz, THF-d_8): δ 158.4, 155.0, 149.5, 147.9, 138.4, 138.0, 135.8, 132.5, 132.0, 130.6, 130.4, 130.0, 129.0, 124.8, 123.0, 122.8, 122.2, 121.5, 87.6, 34.5, 31.3 ppm.

IR (ATR): $\tilde{\nu}$ 3054 (w), 2960 (w), 2902 (w), 2865 (w), 1600 (w) 1492 (m) 1450 (m) 1390 (m) 1249 (w), 1163 (w), 1060 (m), 1007 (m), 963 (w), 889 (w), 808 (s) 772 (m), 688 (m), 605 (w) cm^{-1}.

UV/Vis (CH_2Cl_2): λ_{max} (ε in L mol^{-1} cm^{-1}) 237 (75800), 293 (60500), 310 (33300), 321 (16300) nm.

MALDI HRMS (DCTB): *m/z* calcd. for $C_{69}H_{57}NO_3{}^{97}Br_3{}^{81}Br_3$ $[M]^+$: 1426.9372, found: 1426.9395.

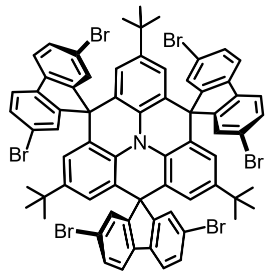

FTN-Br$_6$

2,2'',2''',7,7'',7'''-Hexabromotrispiro[fluorene-9,4'-benzol[1,9]quinolizino[3,4,5,6,7-*defg*]acridine-12',9''-fluorene-8',9'''-fluorene] (FTN-Br$_6$)

Compound **2** (178 mg, 0.124 mmol) was dissolved in CF_3SO_3H (4 mL) and stirred at 0 °C for 5 min. Subsequently, the blue reaction mixture was treated with ice and was neutralized with sat. aq. NaOH (10 mL). The obtained mixture was extracted with CH_2Cl_2 (3 × 100 mL) and the combined organic phases were dried over Na_2SO_4. After removing the solvent under reduced pressure, column chromatography (SiO_2, PE/CH_2Cl_2 2:1) yielded **FTN-Br$_6$** (153 mg, 0.111 mmol, 90%) as a colorless solid.

M.p. (TGA): >400 °C (decomp.).

R_f = 0.58 (SiO_2, PE/CH_2Cl_2 2:1).

^{1}H NMR (600 MHz, $CDCl_3$): δ 7.63 (d, J = 8.1 Hz, 6H), 7.51 (dd, J = 8.1 Hz, 1.8 Hz, 6H), 7.48 (d, J = 1.8 Hz, 6H), 6.33 (s, 6H), 0.78 (s, 27H) ppm.

^{13}C NMR (151 MHz, $CDCl_3$): δ 158.4, 146.0, 137.3, 131.3, 130.8, 129.1, 124.6, 123.6, 122.6, 121.5, 57.5, 33.9, 30.8 ppm.

IR (ATR): $\tilde{\nu}$ 2960 (w), 2902 (w), 1592 (w), 1570 (w), 1541 (s), 1394 (m), 1363 (w), 1325 (w), 1294 (w), 1248 (w), 1223 (m), 1060 (m), 1005 (w), 939 (m), 973 (w), 846 (w), 807 (s), 751 (s), 704 (w), 599 (w) cm^{-1}.

UV/Vis (CH_2Cl_2): λ_{max} (ε in L mol^{-1} cm^{-1}) 250 (83100), 278 (67300), 287 (74500), 300 (51700), 308 (43300), 321 (45700), 350 (7290) nm.

MALDI HRMS (DCTB): *m/z* calcd. for $C_{69}H_{51}N^{97}Br_3{}^{81}Br_3$ $[M]^+$: 1372.9055, found: 1372.9053.

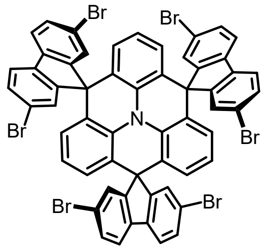

FTN-H-Br$_6$

2',6',10'-Trispiro[2,7-dibromo-9*H*-fluorene-9,4'-benzo[1,9]quinolizino[3,4,5,6,7-*defg*]acridine-12',9''-2,7-dibromo-9*H*-fluorene-8',9'''-2,7-dibromo-9*H*-fluorene] (FTN-H-Br$_6$)

To a solution of **FTN-Br$_6$** (15.0 mg, 10.9 μmol) in dry benzene (1.5 mL), $AlCl_3$ (4.37 mg, 32.8 μmol) was added under nitrogen atmosphere. The reaction mixture was stirred at 40 °C for 1 h in a microwave reaction vessel. Afterwards, the reaction mixture was treated with water (1 mL) and aq. HCl (2 M, 1 mL) and was extracted with CH_2Cl_2 (3 × 50 mL). The combined organic phases were dried over Na_2SO_4, filtered, and the solvent was removed under reduced pressure. The residue was dissolved in hot THF and the solution was treated with MeOH until precipitate was formed. The obtained precipitate was filtered off and washed with MeOH to give **FTN-H-Br$_6$** (8.55 mg, 7.09 μmol, 65%) as a colorless solid.

M.p. (TGA): >500 °C (decomp.).

R_f = 0.82 (SiO_2, PE/CH_2Cl_2 1:1).

^{1}H NMR (600 MHz, THF-*d$_8$*): δ 7.82 (d, *J* = 8.2 Hz, 6H), 7.58 (dd, *J* = 8.2 Hz, 1.8 Hz, 6H), 7.52 (d, *J* = 1.7 Hz, 6H), 6.56 (t, *J* = 7.7 Hz, 3H), 6.37 (d, *J* = 7.7 Hz, 6H) ppm.

^{13}C NMR (151 MHz, THF-*d$_8$*): δ 159.5, 138.2, 133.4, 132.2, 129.4, 128.6, 124.9, 124.7, 123.3, 122.8, 57.6 ppm.

IR (ATR): $\tilde{\nu}$ 3066 (w), 2955 (w), 2923 (w), 2857 (w), 1880 (w), 1746 (w), 1593 (m), 1436 (s), 1393 (s), 1321 (m), 1290 (s), 1248 (m), 1218 (m), 1159 (w), 1128 (w), 1060 (s), 1006 (s), 932 (m), 905 (w), 806 (s), 739 (s), 667 (s), 652 (m), 624 (m) cm^{-1}.

UV/Vis (CH_2Cl_2): λ_{max} (ε in L mol^{-1} cm^{-1}) = 252 (89900), 279 (73400), 287 (7910), 299 (56300), 308 (46400), 320 (46600), 340 (8100) nm.

MALDI-HRMS (DCTB): *m/z* calcd. for $C_{57}H_{27}N^{97}Br_3{}^{81}Br_3$ $[M]^+$: 1204.7177, found: 1204.7184.

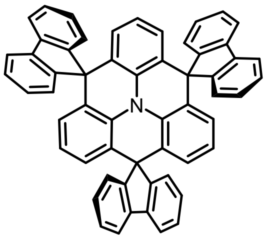

FTN-H

Trispiro[fluorene-9,4'-benzo[1,9]quinolizino[3,4,5,6,7-*defg*]acridine-12',9''-fluorene-8',9'''-fluorene] (FTN-H)

FTN (20 mg, 22.2 μmol) was dissolved in dry benzene (2 mL) and $AlCl_3$ (8.89 mg, 66.7 mmol) was added under nitrogen atmosphere. The reaction mixture was stirred at 40 °C for 1 h. After cooling to room temperature, the reaction mixture was treated with water (2 mL) and aq. HCl (2 M, 2 mL), and was extracted with CH_2Cl_2 (3 × 20 mL). The combined organic phases were dried over Na_2SO_4, filtered, and the solvent was removed under reduced pressure. Column chromatography (SiO_2, PE/CH_2Cl_2 3:1) gave **FTN-H** (12.9 mg, 17.6 μmol, 79%) as a colorless solid.

M.p. (DSC): 411 °C.

R_f = 0.20 (SiO_2, PE/CH_2Cl_2 3:1).

^1H NMR (600 MHz, CD_2Cl_2): δ 7.83 (d, J = 7.6 Hz, 6H), 7.43–7.39 (m, 12H), 7.32 (td, J = 7.6 Hz, 0.9 Hz, 6H), 6.47 (t, J = 7.7 Hz, 3H), 6.33 (d, J = 7.7 Hz, 6H) ppm.

^{13}C NMR (151 MHz, CD_2Cl_2): δ 157.3, 139.7, 133.6, 129.0, 128.2, 127.6, 125.9, 125.8, 123.5, 120.6, 57.1 ppm.

IR (FT-ATR): $\tilde{\nu}$ 3038 (w), 2924 (w), 2851 (w), 1733 (w), 1591 (m), 1429 (s), 1317 (m), 1276 (m), 1219 (m), 1164 (w), 1120 (w), 1030 (w), 923 (m), 773 (s), 731 (s), 626 (m), 547 (m) cm^{-1}.

UV/Vis (CH_2Cl_2): λ_{max} (ε in L mol^{-1} cm^{-1}) 251 (67400), 275 (36800), 300 (24000), 310 (32800), 336 (6350) nm.

MALDI HRMS (DCTB): *m/z* calcd. for $C_{57}H_{34}N$ $[M+H]^+$: 732.2686, found: 732.2689.

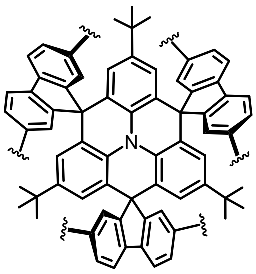

FTN-Pol

2',6',10'-Tri-*tert*-butyltrispiro[fluorene-9,4'-benzo[1,9]quinolizino[3,4,5,6,7-*defg*]-acridine-12',9''-fluorene-8',9'''-fluorene]-based polymer (FTN-Pol)

[Ni(COD)$_2$] (216 mg, 786 μmol), COD (85.1 mg, 96.7 μL, 786 μmol), and bpy (123 mg, 786 μmol) were dissolved in dry and degassed DMF (15 mL) in a Schlenk tube. The catalyst mixture was stirred at 80 °C for 1 h. After heating, **FTN-Br$_6$** (150 mg, 109 μmol) was added in one portion to the dark blue solution and the reaction mixture was stirred at 80 °C for 2 days. After cooling to room temperature, the mixture was treated with conc. HCl (15 mL). The obtained pale yellow to orange precipitate was washed with MeOH, EtOH, CH_2Cl_2, $CHCl_3$, and THF and was subsequently purified via *Soxhlet* extraction with THF to obtain the desired product **FTN-Pol** (56.6 mg, 63.3 μmol, 58%) after drying in vacuo at 100 °C for 12 h.

M.p. (TGA): >500 °C (decomp.).

IR (ART): $\tilde{\nu}$ 3033 (w), 2364 (w), 2923 (w), 1601 (m), 1447 (s), 1289 (m), 1193 (m), 1000 (m), 816 (w), 737 (s), 654 (w), 561 (s) cm^{-1}.

Elemental analysis: calcd. for $C_{69}H_{51}N$: C, 92.68%; H, 5.75%; N, 1.57%; found: C, 87.11%; H, 6.26%; N, 1.93%.

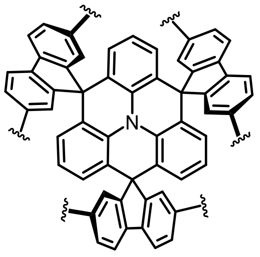

FTN-H-Pol

Trispiro[fluorene-9,4'-benzo[1,9]quinolizino[3,4,5,6,7-*defg*]acridine-12',9''-fluorene-8',9'''-fluorene]-based polymer (FTN-H-Pol)

[Ni(COD)$_2$] (104 mg, 376 μmol), COD (40.7 mg, 46.2 μL, 376 μmol), and bpy (58.8 mg, 376 μmol) were dissolved in dry and degassed DMF (7 mL) in a Schlenk tube. The catalyst mixture was stirred at 80 °C for 1 h. After heating, compound **FTN-H** (63.0 mg, 52.3 μmol) was added in one portion to the dark blue solution and the reaction mixture was stirred at 80 °C for 2 days. After cooling to room temperature, the reaction mixture was treated with conc. HCl (5 mL). The obtained pale yellow precipitate was washed with MeOH, EtOH, CH_2Cl_2, $CHCl_3$, and THF and was subsequently purified via *Soxhlet* extraction with THF. After drying in vacuo at 100 °C for 12 h the desired polymer **FTN-H-Pol** (18.2 mg, 29.8 μmol, 55%) was obtained.

M.p. (TGA): >700 °C (decomp.).

IR (ART) $\tilde{\nu}$ 2359 (s), 2342 (s), 1669 (m), 1434 (s), 1318 (m), 1286 (m), 1215 (m), 1064 (w), 816 (m), 735 (s), 669 (m) cm^{-1}.

Elemental analysis: calcd. for $C_{57}H_{27}N$: C, 94.32%; H, 3.75%; N, 1.93%; found: C, 84.20%; H, 4.54%; N, 2.32%.

3. Nuclear Magnetic Resonance Spectra

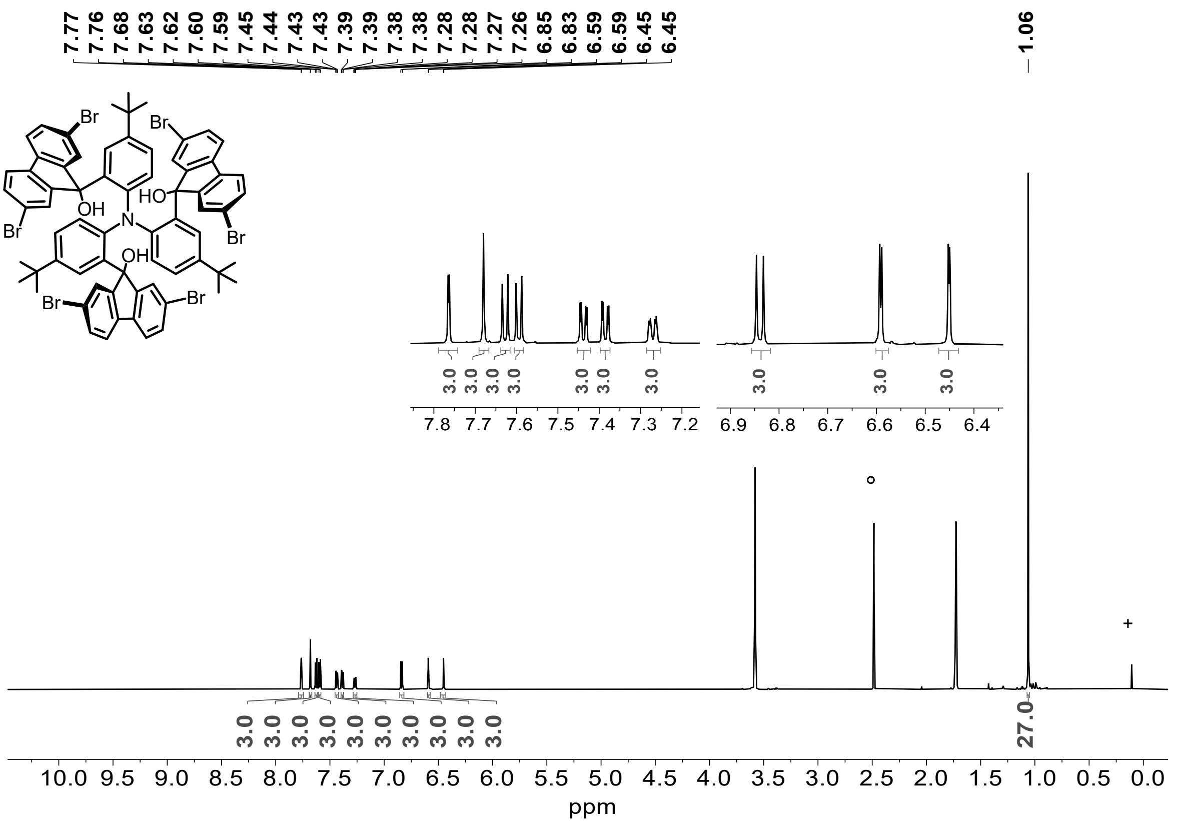

Figure S1: ^{1}H NMR spectrum of **2** (600 MHz, THF-d_8, rt); °H_2O, $^+$ silicon grease.

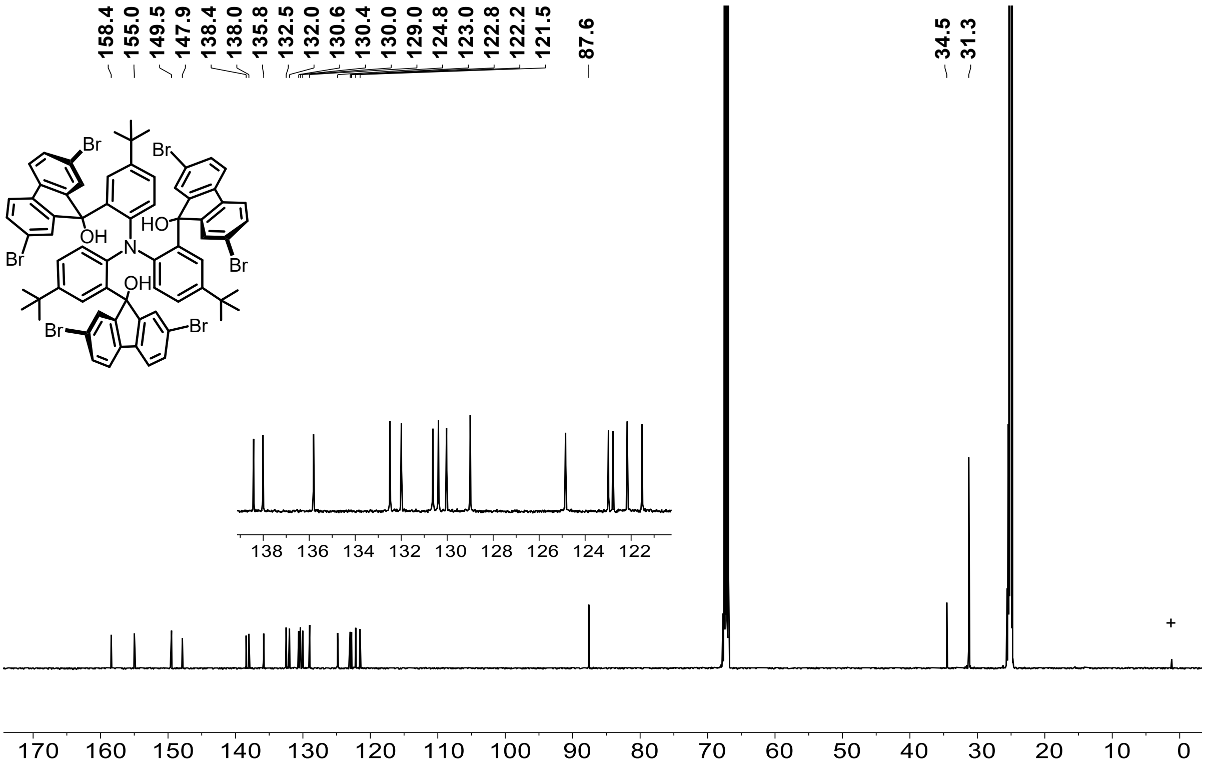

Figure S2: ^{13}C NMR spectrum of **2** (151 MHz, THF-d_8, rt).

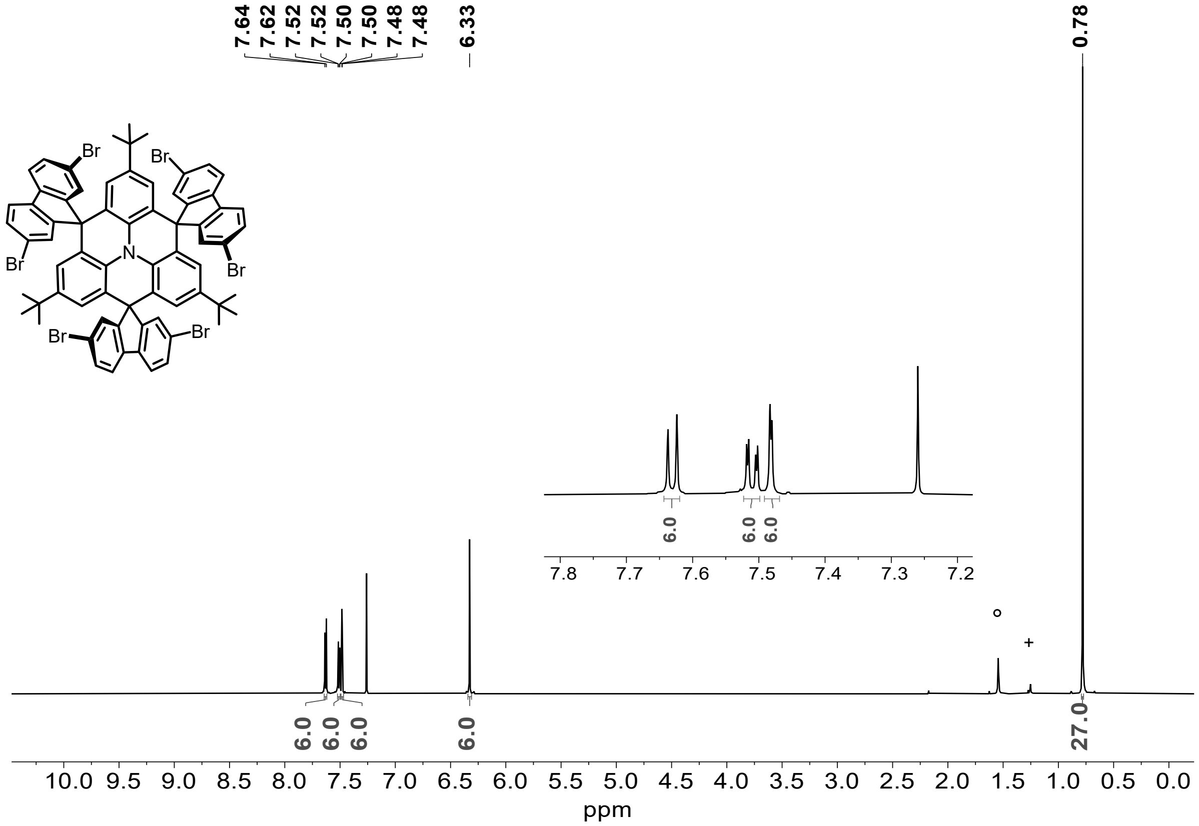

Figure S3: ^{1}H NMR spectrum of **FTN-Br$_6$** (600 MHz, $CDCl_3$, rt); °H_2O, ^{+}H grease.

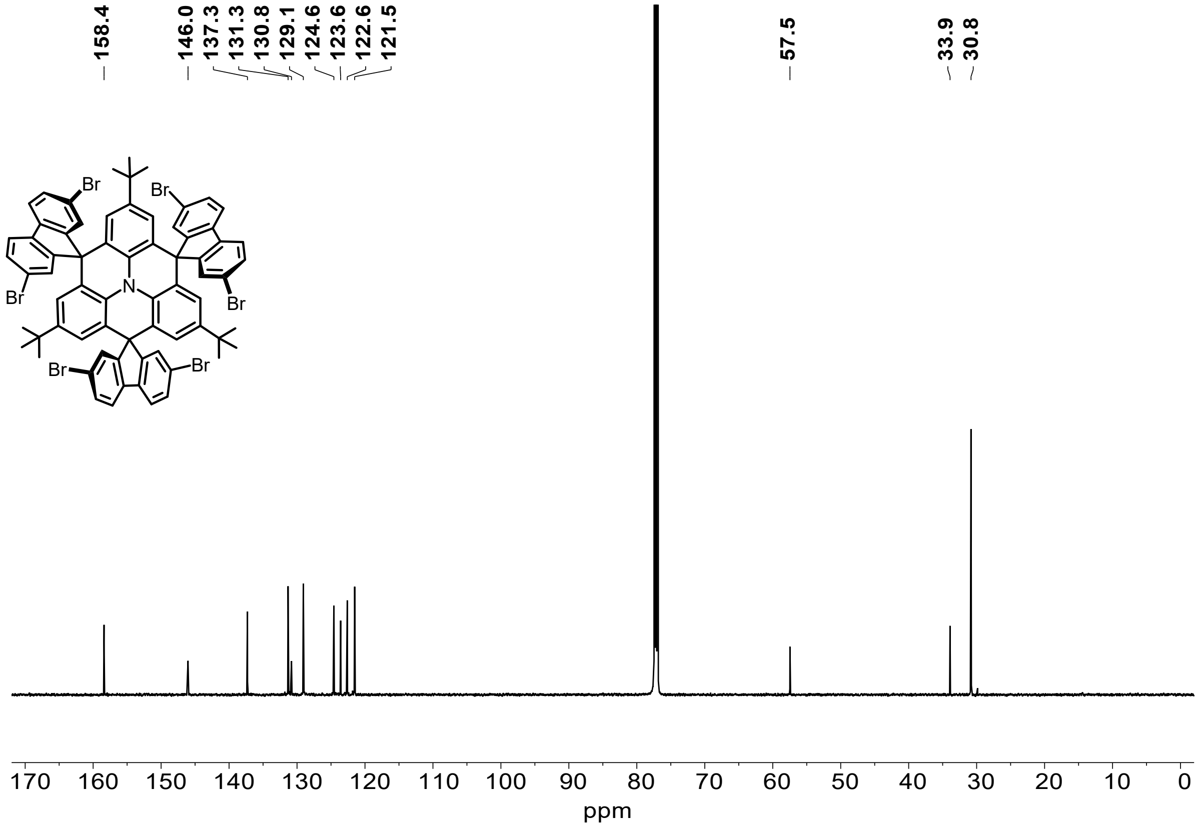

Figure S4: ^{13}C NMR spectrum of **FTN-Br$_6$** (151 MHz, $CDCl_3$, rt).

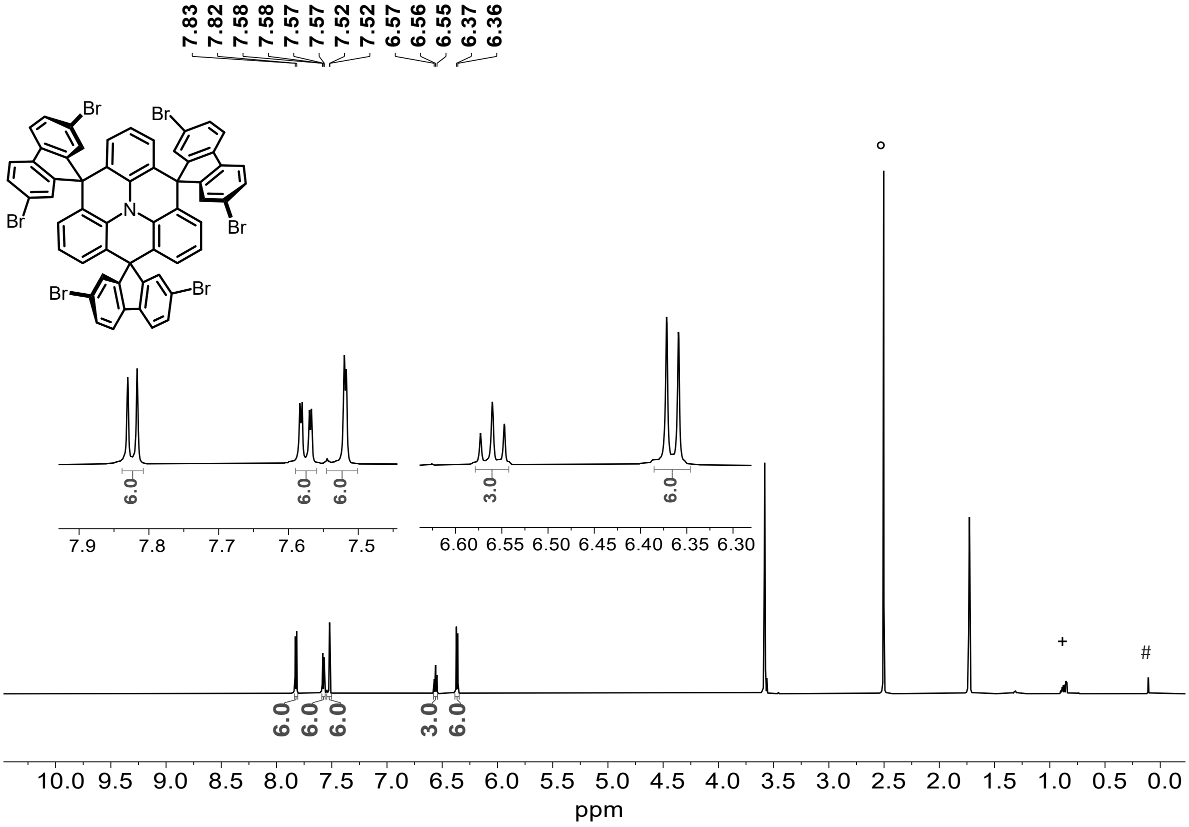

Figure S5: ^{1}H NMR spectrum of **FTN-H-Br$_6$** (600 MHz, THF-d_8, rt); °H_2O, $^{+}$H grease, $^{\#}$silicon grease.

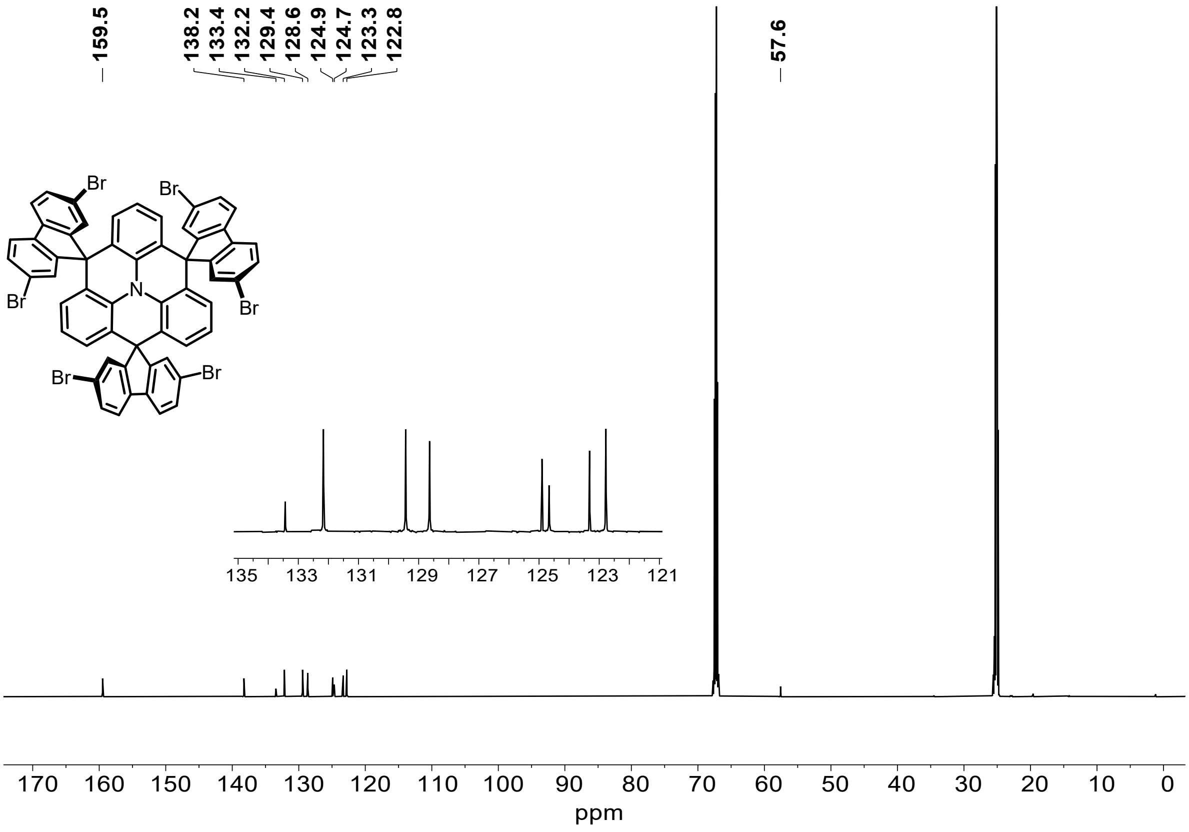

Figure S6: ^{13}C NMR spectrum of **FTN-H-Br$_6$** (151 MHz, THF-d_8, rt).

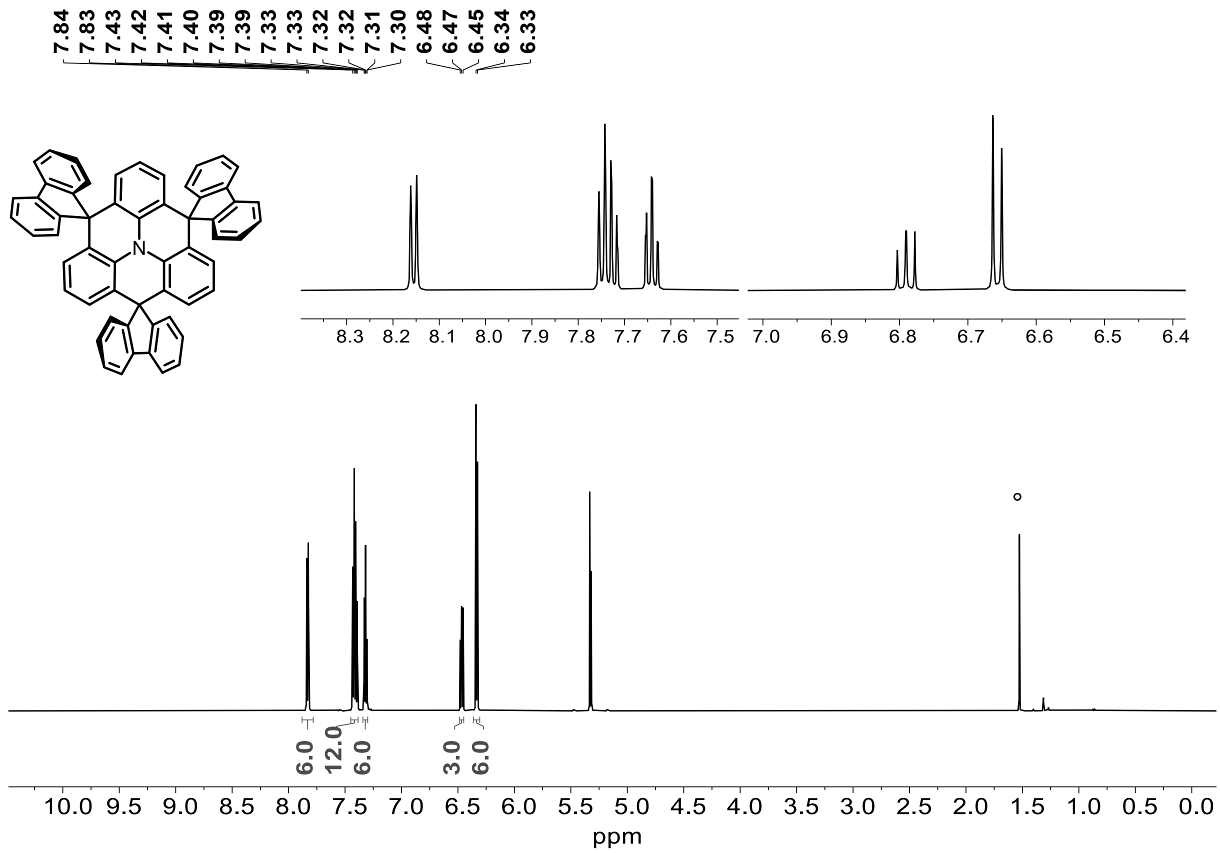

Figure S7: ^{1}H NMR spectrum of **FTN-H** (600 MHz, CD_2Cl_2, rt); °H_2O.

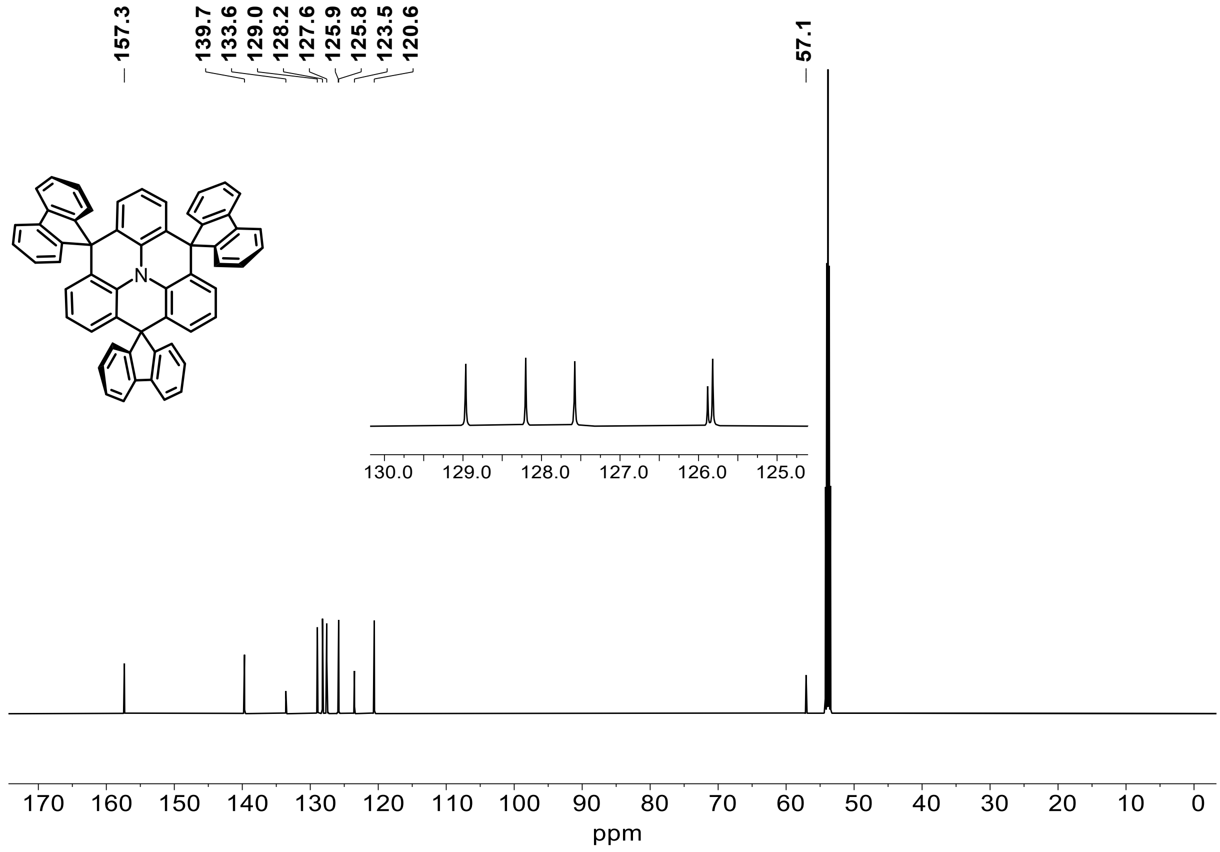

Figure S8: ^{13}C NMR spectrum of **FTN-H** (151 MHz, CD_2Cl_2, rt).

4. Infrared Spectra

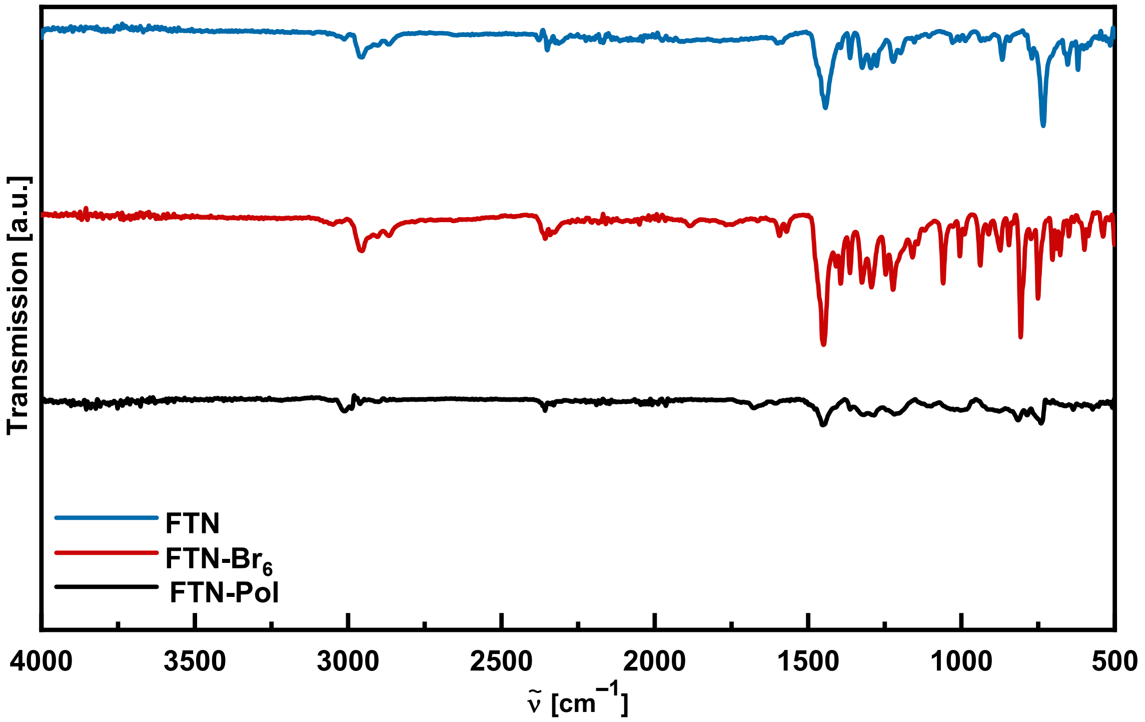

Figure S9: FT-IR (ATR) spectrum of the polymer **FTN-Pol**, the monomer **FTN-Br$_6$**, and the model compound **FTN**.

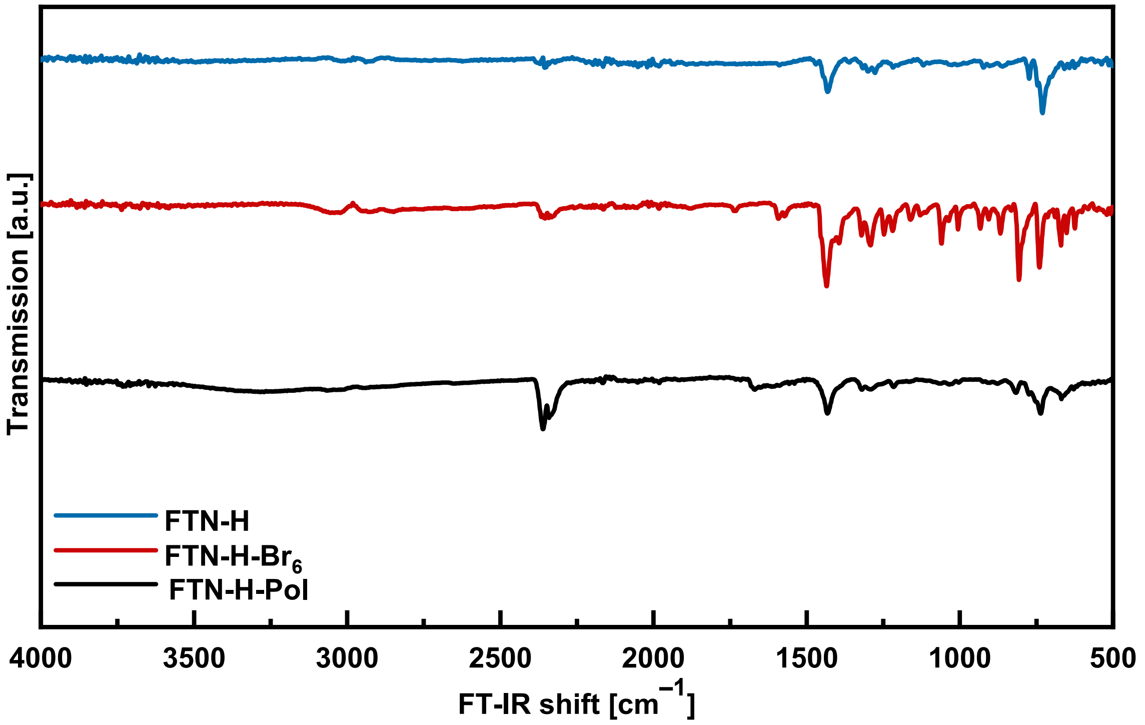

Figure S10: FT-IR (ATR) spectrum of the polymer **FTN-H-Pol**, the monomer **FTN-H-Br$_6$**, and the model compound **FTN-H**.

5. Raman Spectra

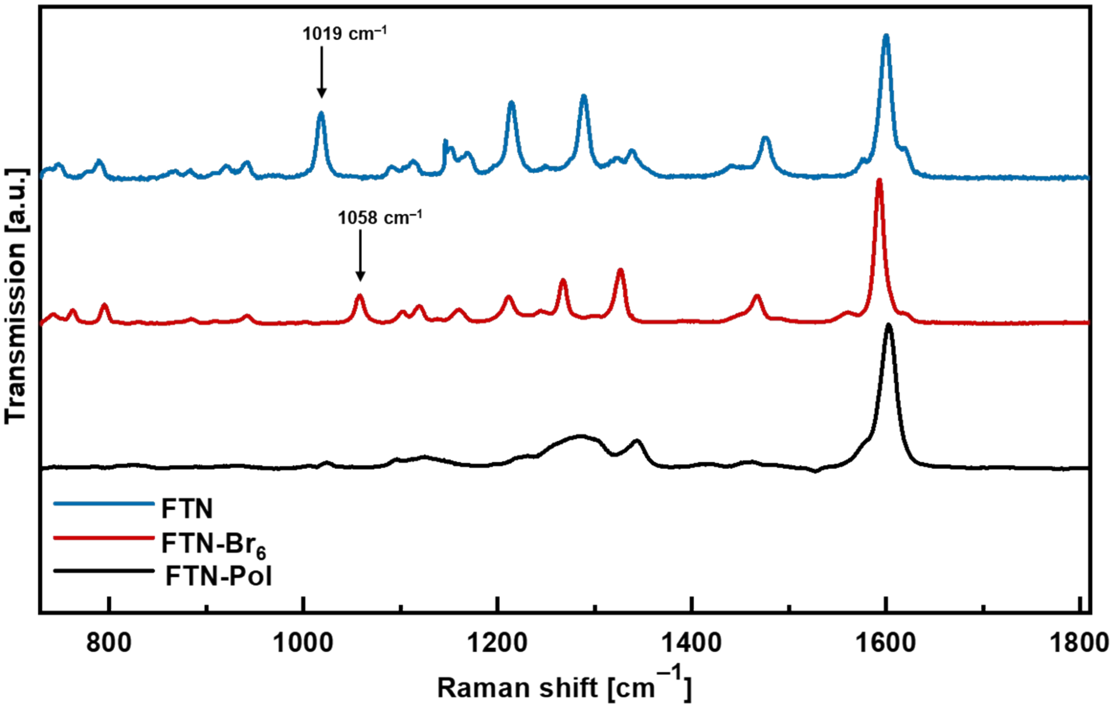

Figure S11: Raman spectrum of the polymer **FTN-Pol**, the monomer **FTN-Br$_6$**, and the model compound **FTN**.

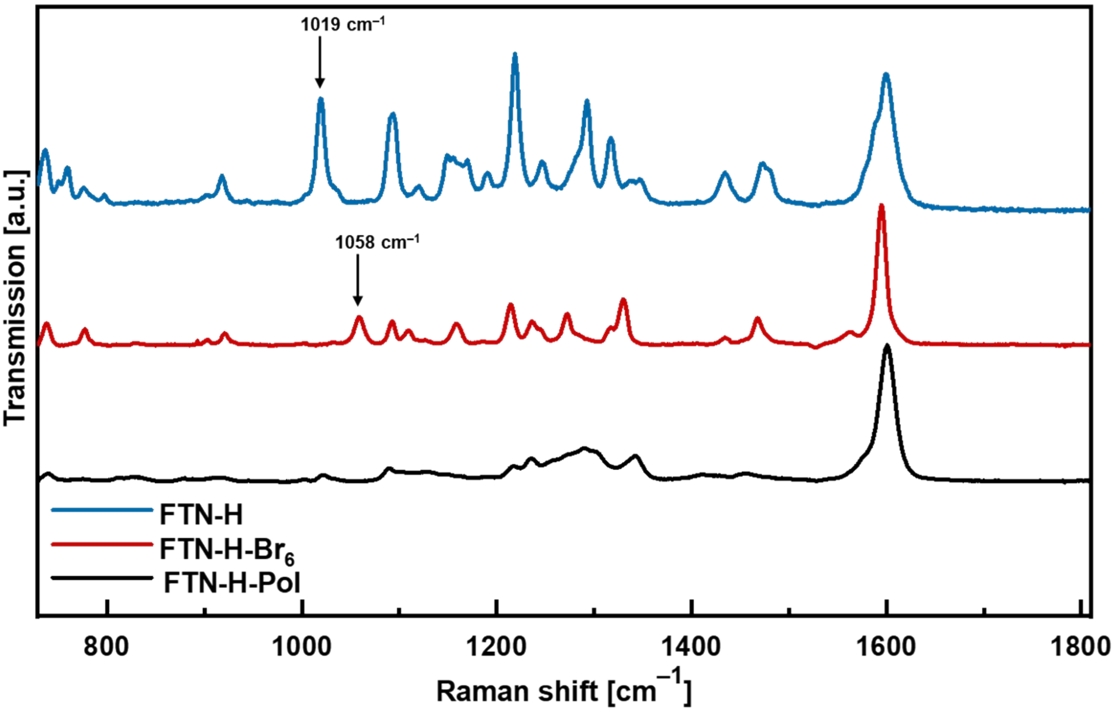

Figure S12: Raman spectrum of the polymer **FTN-H-Pol**, the monomer **FTN-H-Br$_6$**, and the model compound **FTN-H**.

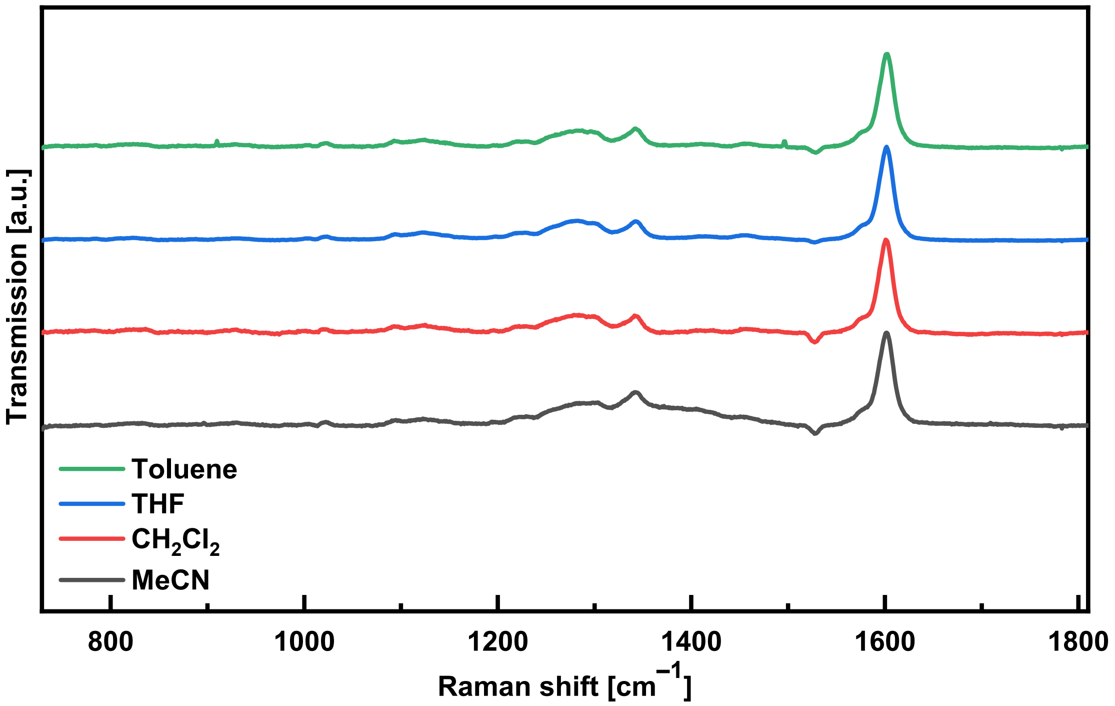

Figure S13: Raman spectrum of the polymer **FTN-Pol** dispersed different solvents.

6. TGA and DSC Measurements

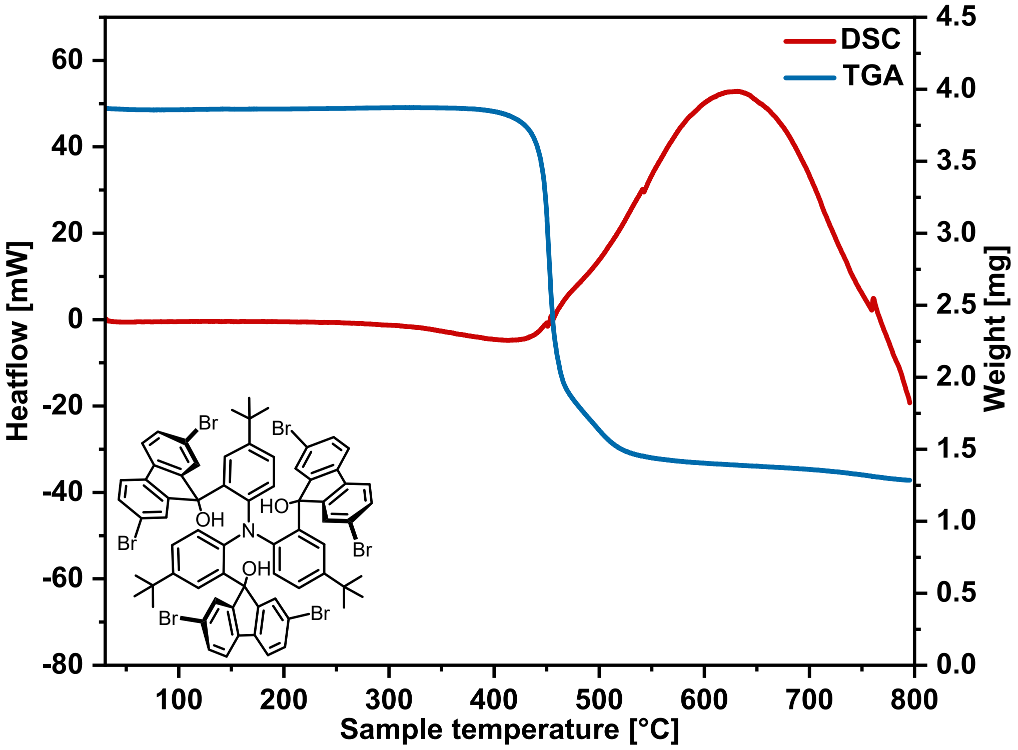

Figure S14: TGA (blue) and DSC (red) curves for compound **2** measured under nitrogen atmosphere.

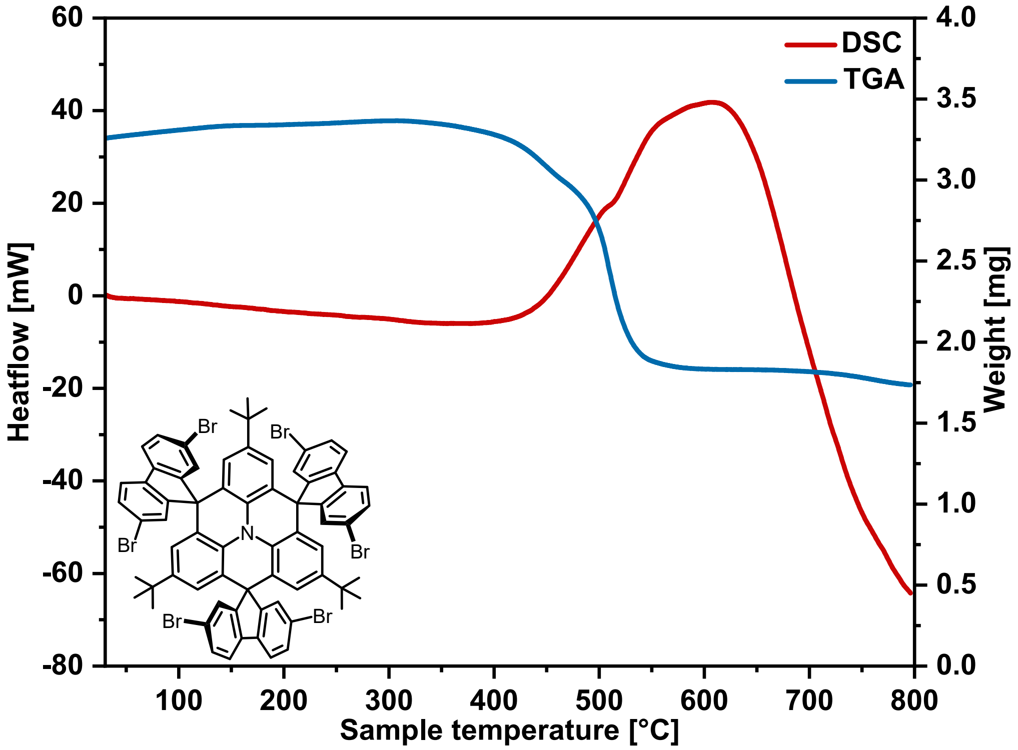

Figure S15: TGA (blue) and DSC (red) curves for **FTN-Br$_6$** measured under nitrogen atmosphere.

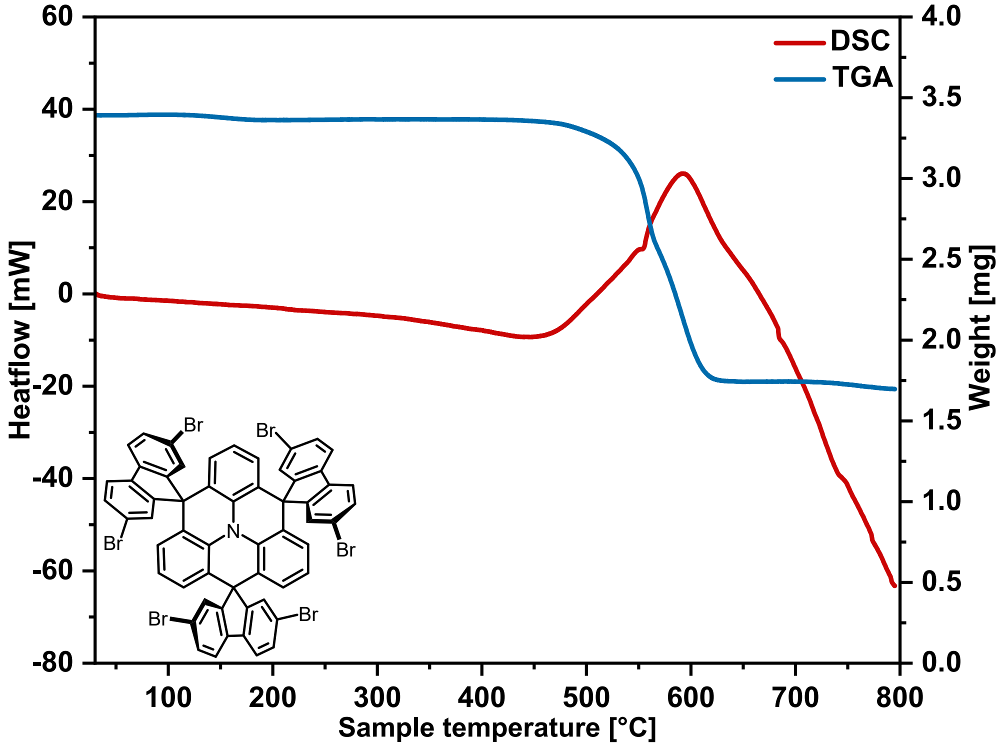

Figure S16: TGA (blue) and DSC (red) curves for **FTN-H-Br$_6$** measured under nitrogen atmosphere.

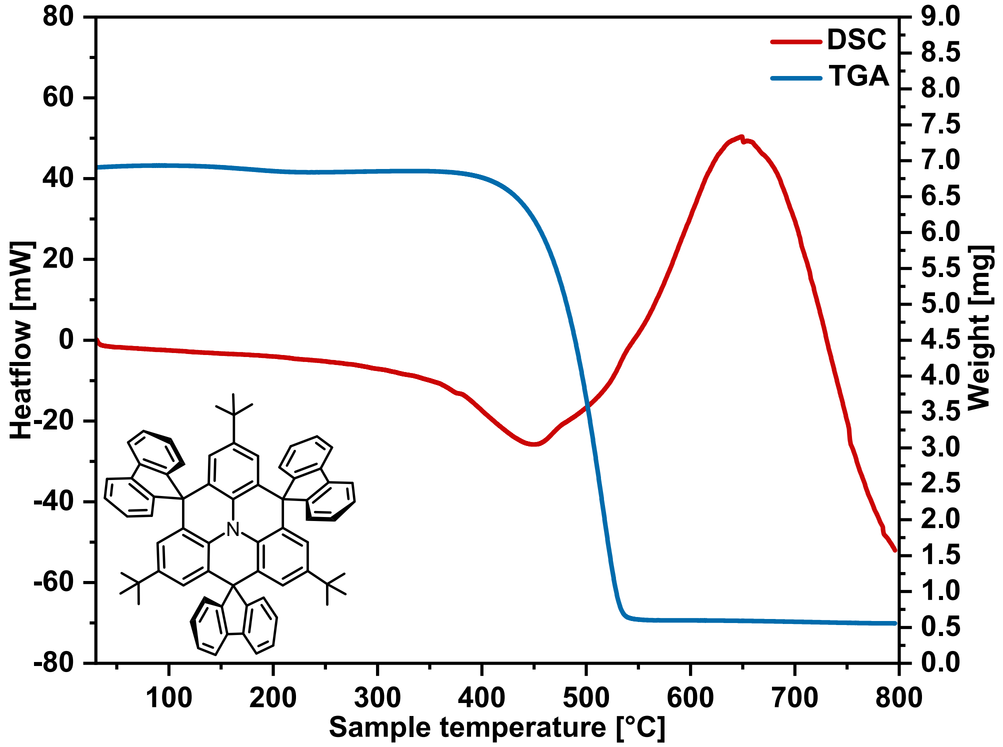

Figure S17: TGA (blue) and DSC (red) curves for **FTN** measured under nitrogen atmosphere.

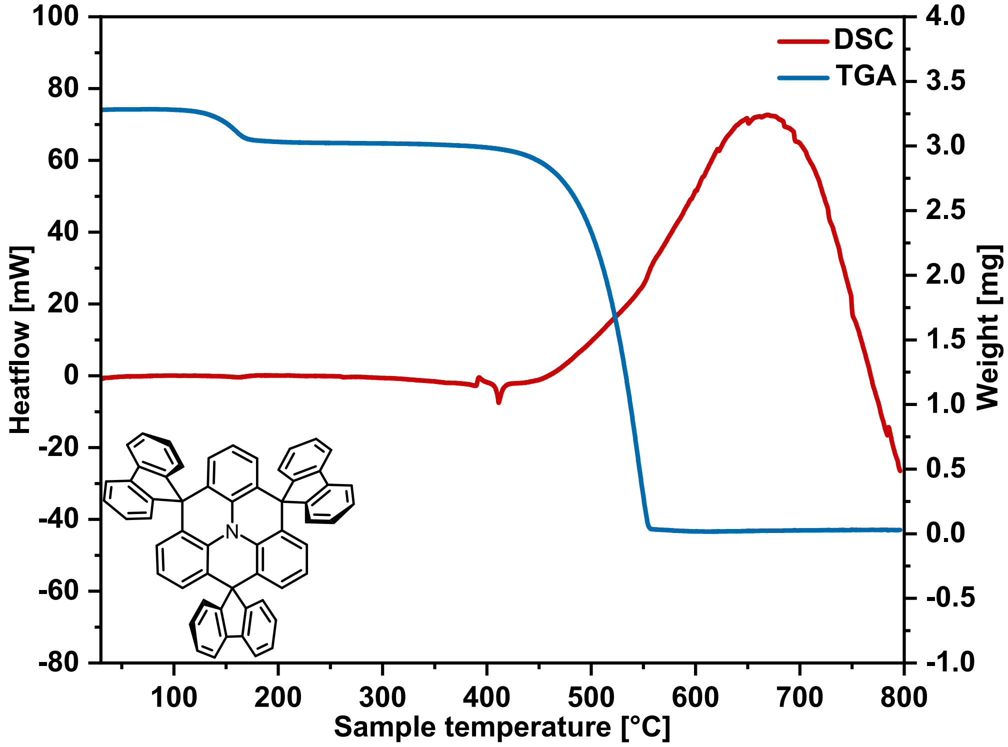

Figure S18: TGA (blue) and DSC (red) curves for **FTN-H** measured under nitrogen atmosphere.

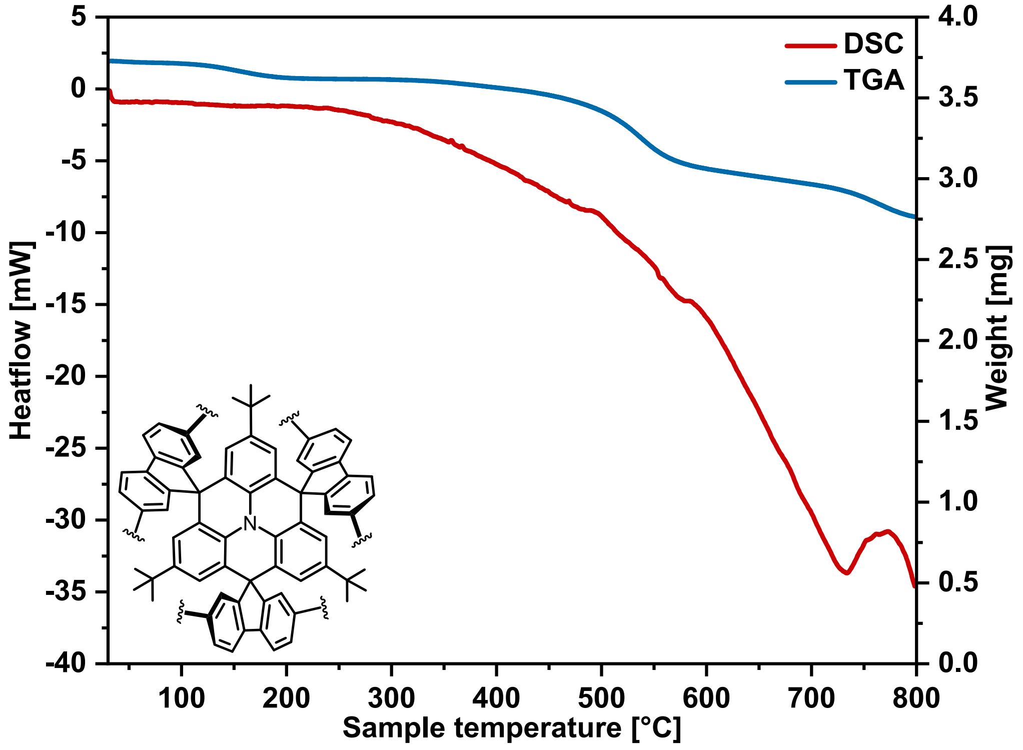

Figure S19: TGA (blue) and DSC (red) curves for the polymer **FTN-Pol** measured under nitrogen atmosphere.

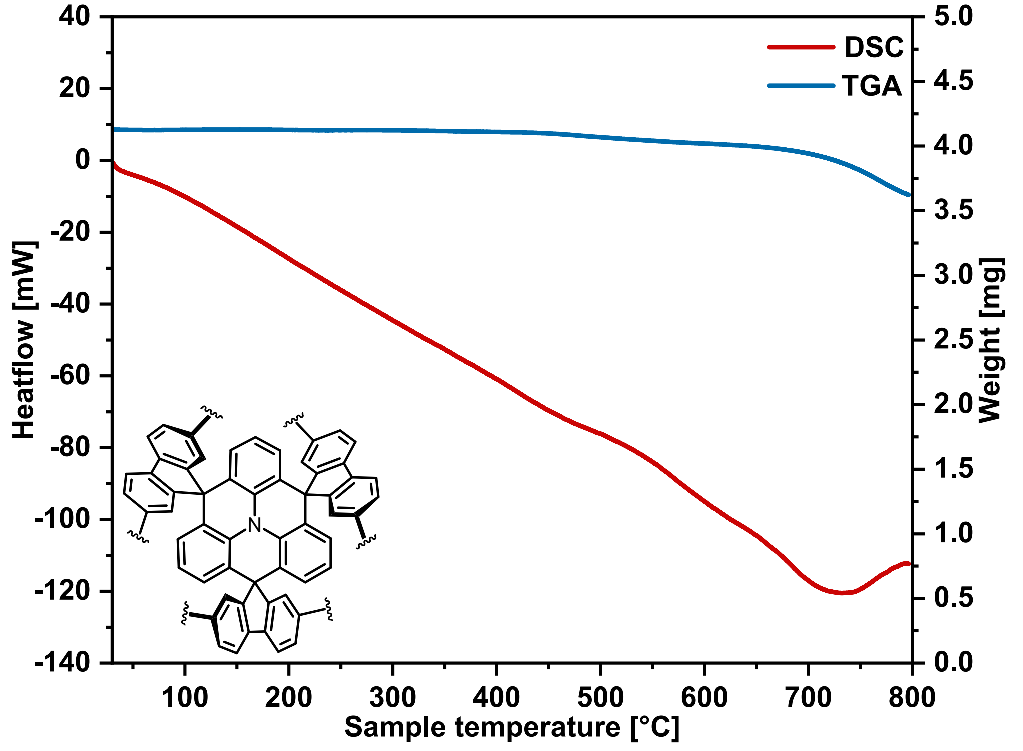

Figure S20: TGA (blue) and DSC (red) curves for the polymer **FTN-H-Pol** measured under nitrogen atmosphere.

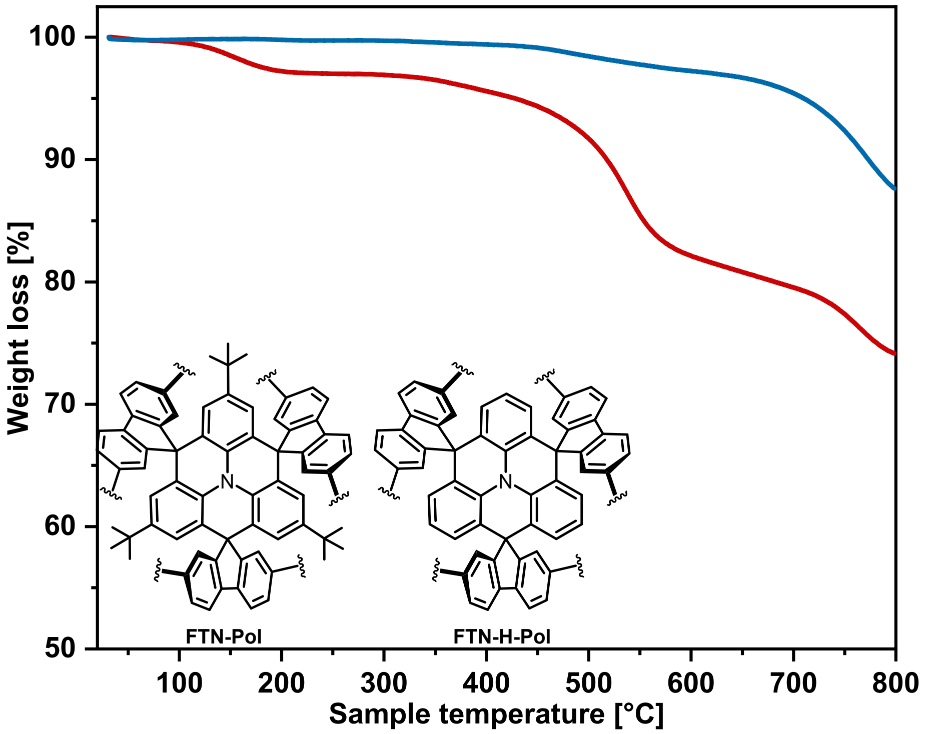

Figure S21: Comparison of the TGA curves for the polymers **FTN-Pol** (red) and **FTN-H-Pol** (blue) measured under nitrogen atmosphere.

Table S1: Melting points (m.p.) and decomposition temperatures (T_d) determined by TGA and DSC measurements of compound **2**, **FTN-Br$_6$**, **FTN-H-Br$_6$**, **FTN**, **FTN-H**, **FTN-Pol**, and **FTN-H-Pol**.

	**2**	**FTN-Br$_6$**	**FTN-H-Br$_6$**	**FTN**	**FTN-H**	**FTN-Pol**	**FTN-H-Pol**
T_d [a]	437	448	522	435	443	492	712
**M.p.**	–	–	–	450	411	–	–

[a] Estimated from the edge of the decomposition curve corresponding to the first weight loss of >5%.

7. Powder X-Ray Diffraction

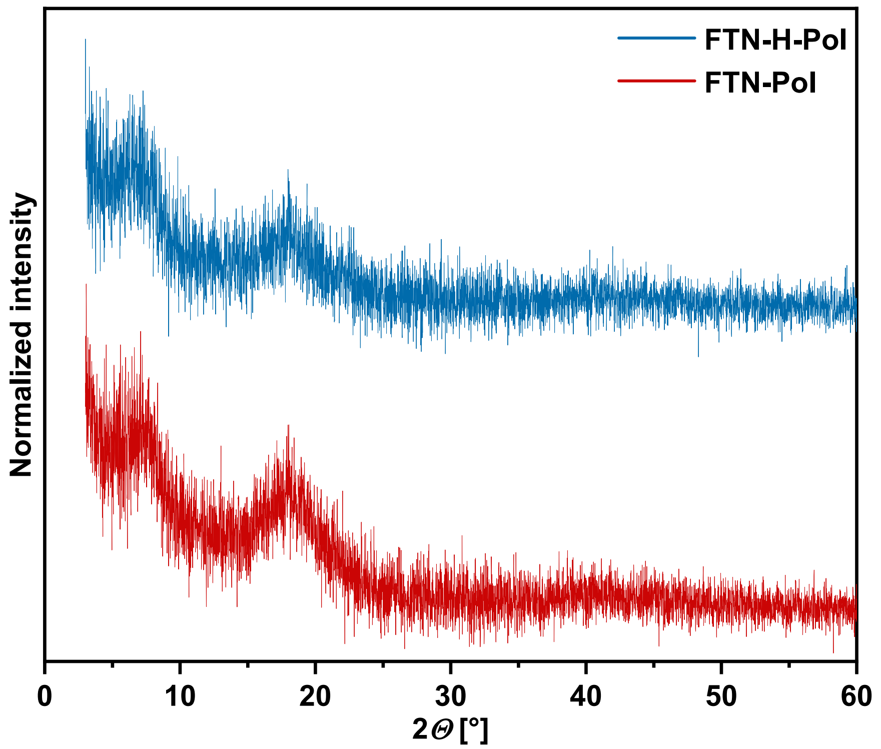

Figure S22: PXRD spectra of **FTN-H-Pol** and **FTN-Pol**.

8. UV/Vis Absorption and Fluorescence Spectroscopy

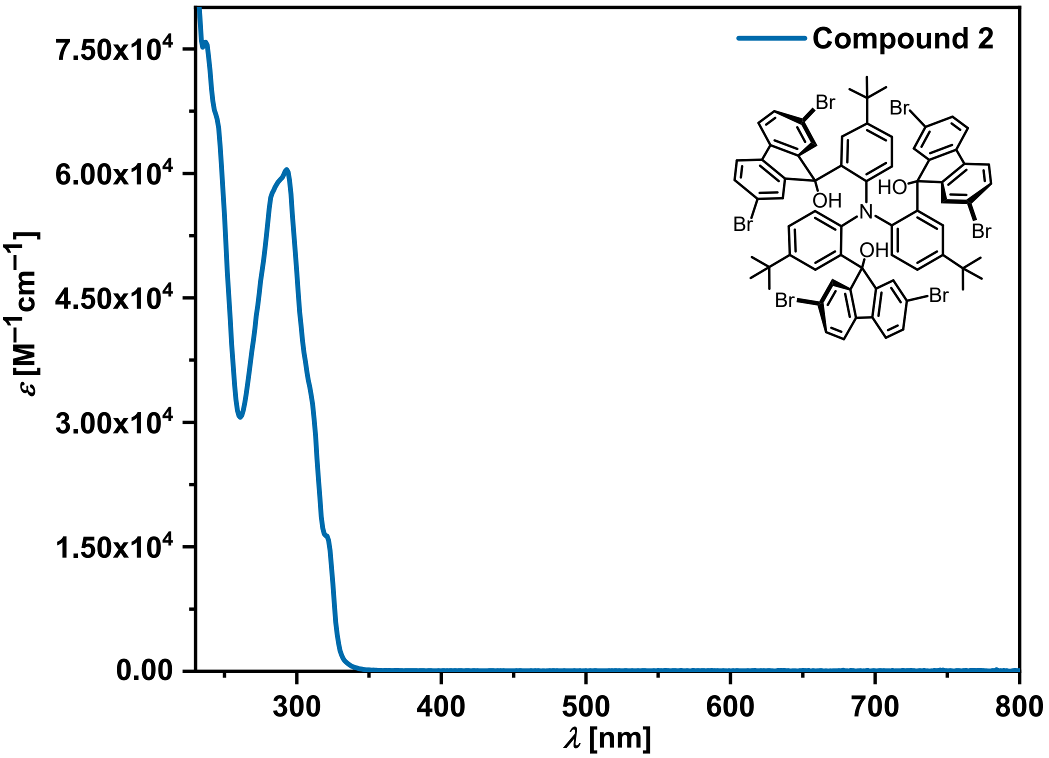

Figure S23: UV/Vis absorption spectrum of compound **2** measured in CH_2Cl_2 at room temperature.

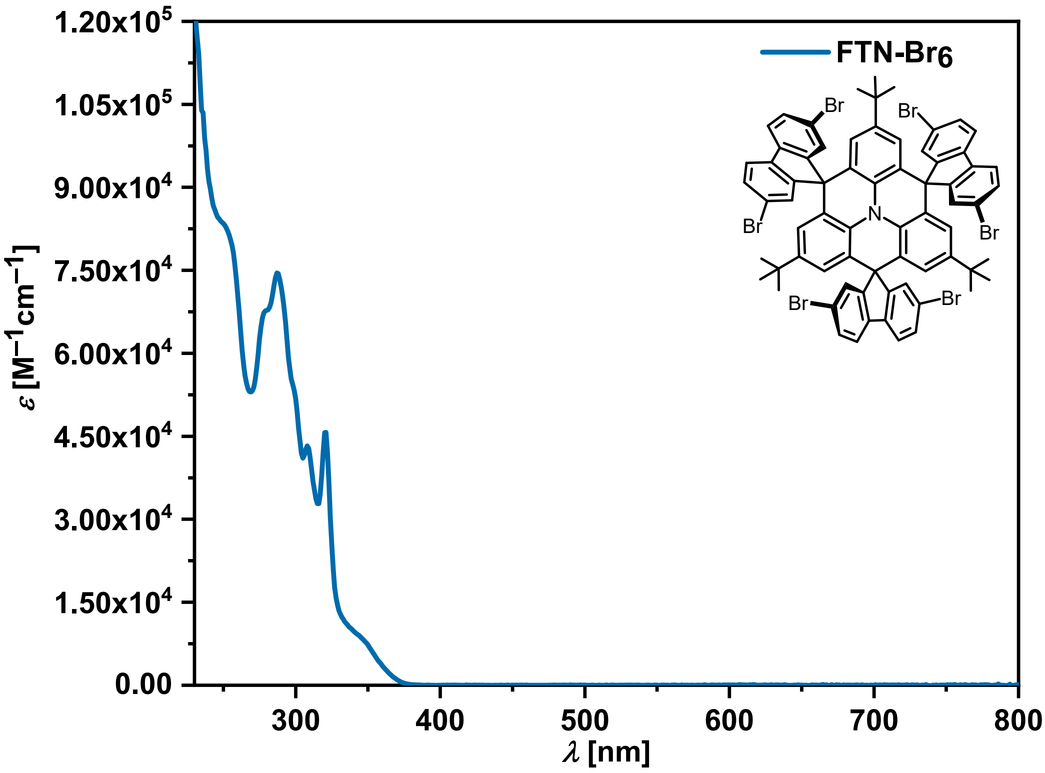

Figure S24: UV/Vis absorption spectrum of **FTN-Br$_6$** measured in CH_2Cl_2 at room temperature.

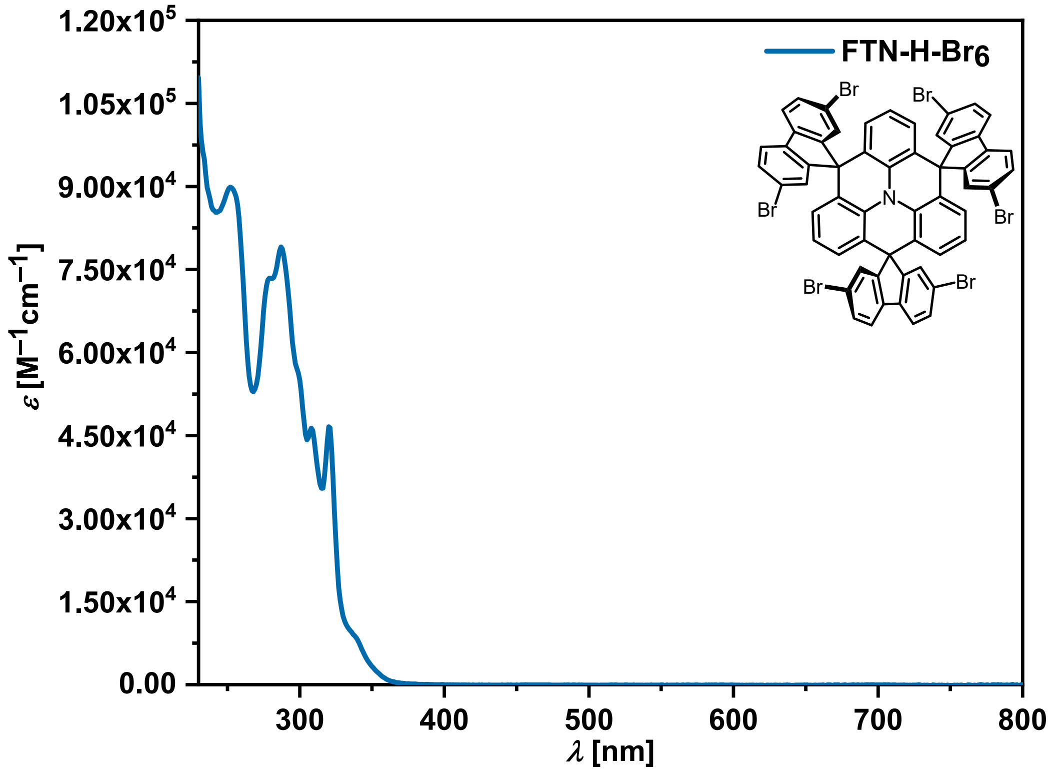

Figure S25: UV/Vis absorption spectrum of **FTN-H-Br$_6$** measured in CH_2Cl_2 at room temperature.

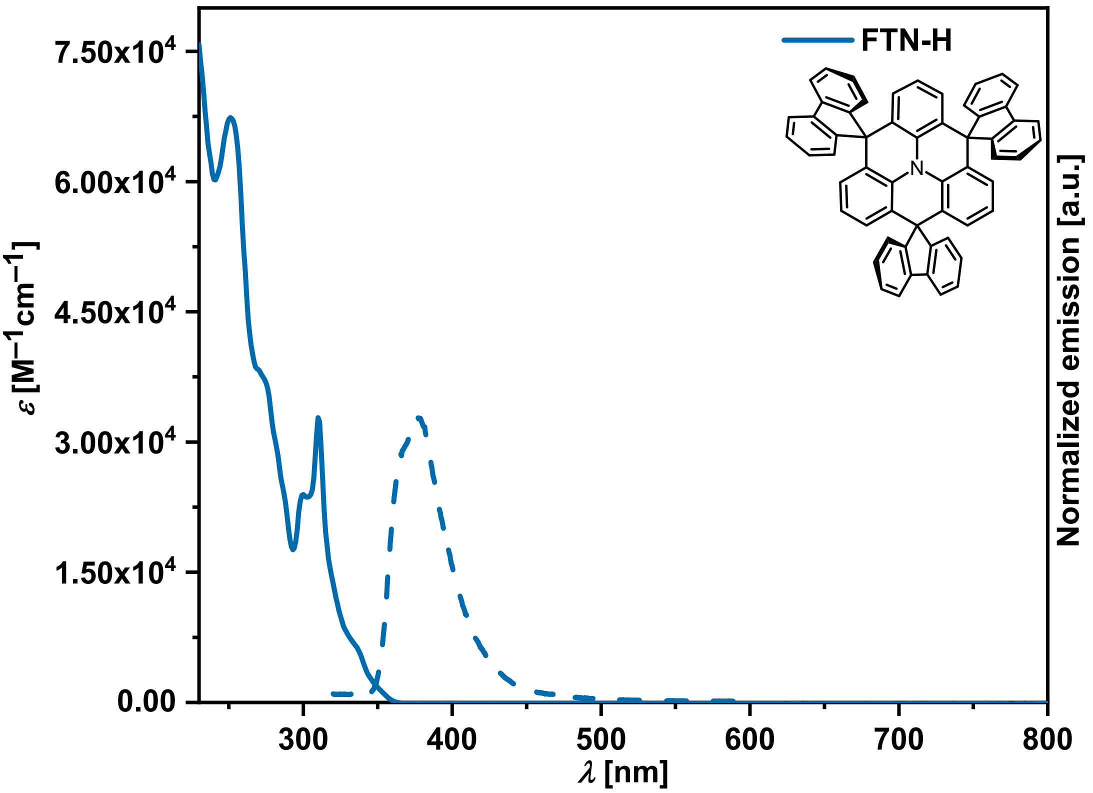

Figure S26: UV/Vis absorption and normalized emission spectra of **FTN-H** measured in CH_2Cl_2 at room temperature.

Table S2: Photophysical properties of compound **2**, **FTN-Br$_6$**, **FTN-H-Br$_6$**, and **FTN-H**.

Compound	λ_{max} [nm][a]	$\varepsilon_{\lambda max}$ [$M^{-1}cm^{-1}$]	$\lambda_{a.e.}$ [nm][b]	$E_{g,UV/Vis}$ [eV][c]	λ_{em} [nm][d]	Φ [%][e]	λ_s [cm^{-1}]
**2**	293	60500	335	3.70	–	–	–
**FTN-Br$_6$**	321	45700	374	3.32	–	–	–
**FTN-H-Br$_6$**	321	46600	361	3.43	–	–	–
**FTN-H**	310	32800	327	3.79	377	4	5733

[a] λ_{max}: Absorption maximum with the lowest energy. [b] Estimated from absorption edge wavelength via tangent method. [c] Calculated from the absorption edge wavelength, $E_{g,UV/Vis} = (h \cdot c)/\lambda_{a.e.}$. [d] Excitation wavelength (λ_{ex}) of 310 nm. [e] Determined using an integrating sphere.

9. Fluorescence Measurements of FTN and FTN-Pol Dispersions

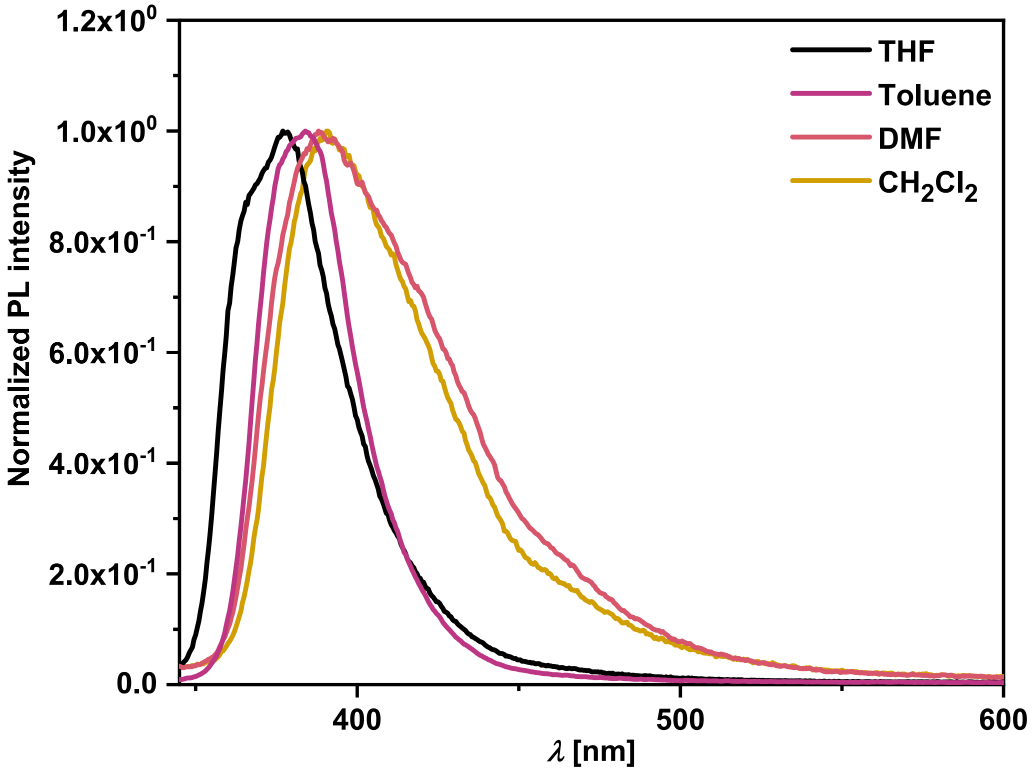

Figure S27: Photoluminescence spectra of **FTN** measured as solution in different organic solvents with an excitation wavelength (λ_{ex}) of 310 nm.

The impact of aggregation on the photoluminescence (PL) properties of **FTN** in different THF/water mixtures has been studied by us previously.[6] The sensitivity of **FTN** in terms of its photophysical properties during dynamic aggregation processes and changing the polarity of the solvent can be most likely related to the spatial separation between the highest occupied molecular orbital (HOMO) located on the *N*-HTA core and the lowest unoccupied molecular orbital (LUMO) residing on the spirofluorene moieties, which provides for intramolecular charge transfer from the electron-rich *N*-HTA core to the peripheral fluorenyls upon photoexcitation.

The observed red-shift of the emission when going from **FTN** to **FTN-Pol** can be most likely ascribed to the extension of the π-conjugated system after the polymerization (Figure S28). In addition, interactions of the individual **FTN** chromophores within the polymer backbone and further assembly of the polymer into larger aggregates are mainly responsible for the observed broadening and the bathochromic shift of the emission spectra.[6–8]

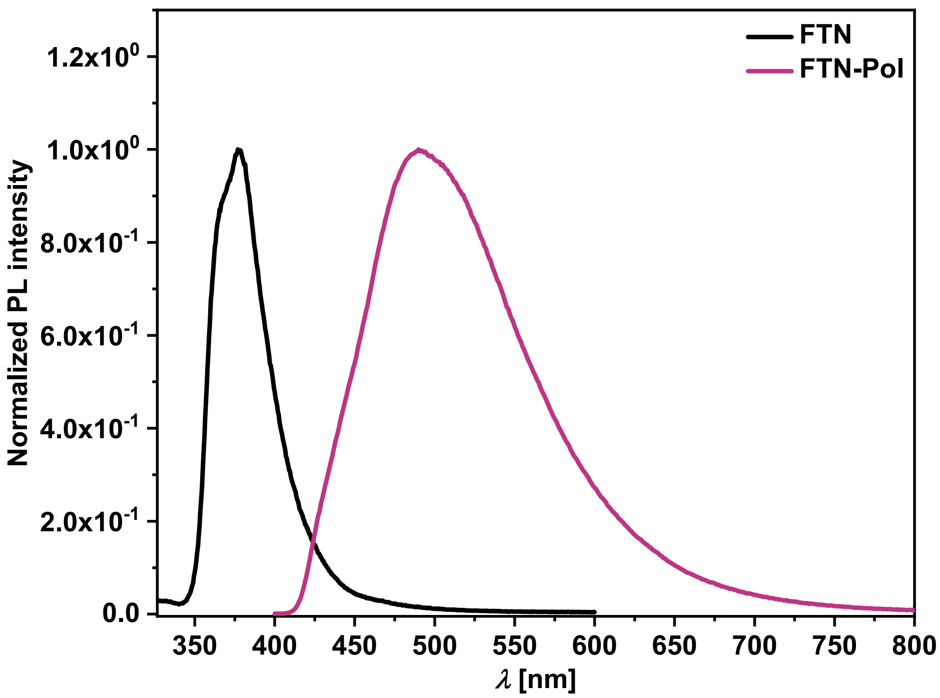

Figure S28: Photoluminescence spectra of **FTN** and **FTN-Pol** measured as solution and dispersion in THF.

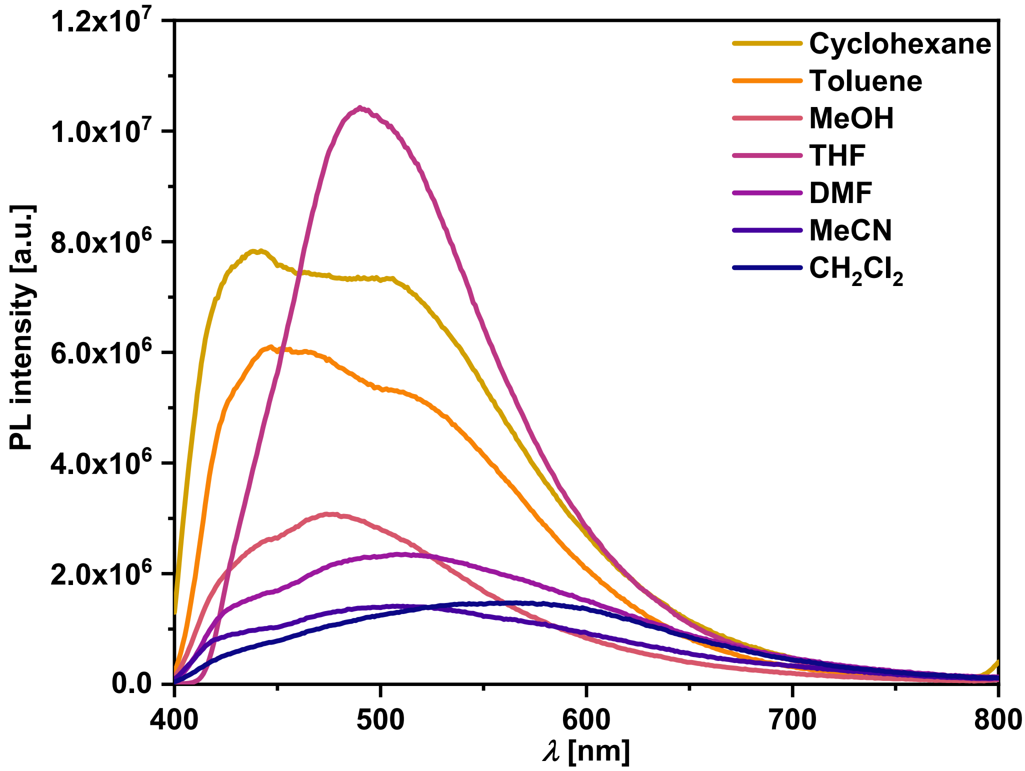

Figure S29: Photoluminescence spectra of **FTN-Pol** measured as dispersion in different organic solvents with an excitation wavelength (λ_{ex}) of 370 nm.

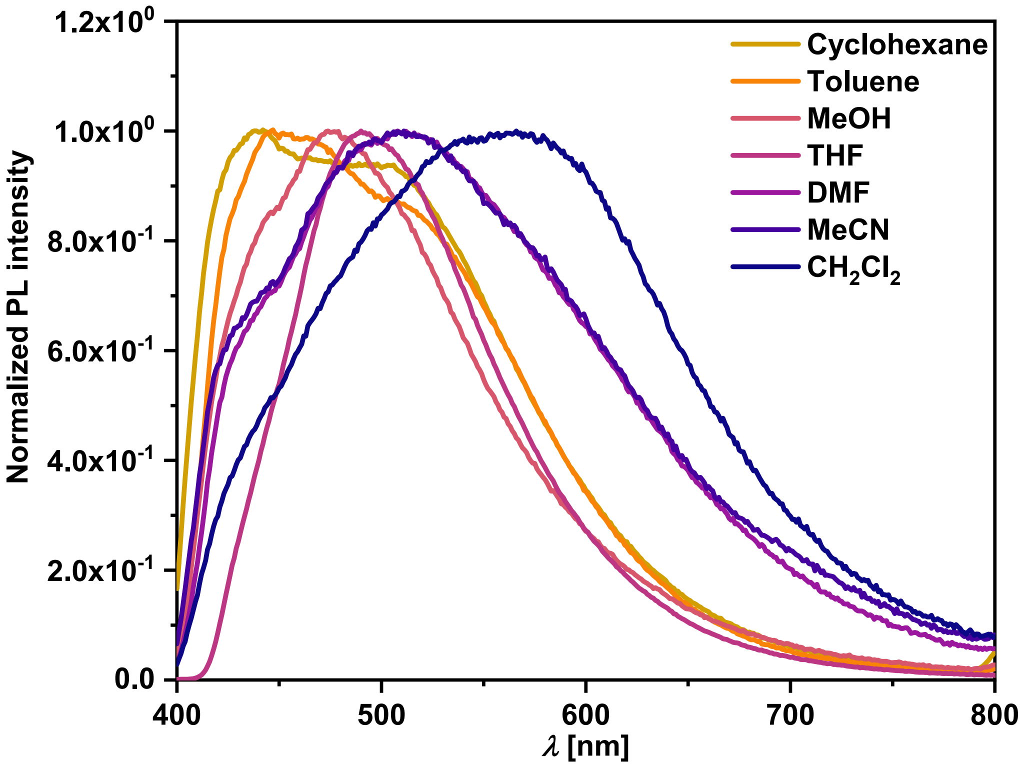

Figure S30: Normalized photoluminescence spectra of **FTN-Pol** measured as dispersion in different organic solvents with an excitation wavelength (λ_{ex}) of 370 nm.

Table S3: Photoluminescent properties of **FTN-Pol dispersions** and **FTN** in different solvents.

Solvent	Cyclohexane	Toluene	MeOH	THF	MeCN	DMF	CH_2Cl_2
Dielectric constant ε [9]	2.02	2.37	32.6	7.40	36.64	36.70	8.90
**FTN** λ_{em} **[nm]**[a]	–	384	–	377 (368)	–	388	391
**FTN** Φ **[%]**[b]	–	3	–	4	–	2	2
**FTN-Pol** λ_{em} **[nm]**[c]	438 (503)	447 (514)	(442) 474	490	508	508	561
**FTN-Pol** Φ **[%]**[b]	2	3	1	3	1	1	1

[a] λ_{em} of **FTN** was determined with an excitation wavelength (λ_{ex}) of 310 nm. The values in the parentheses represent shoulders in the spectra. [b] Determined using an integrating sphere. [c] λ_{em} of **FTN-Pol** was determined with an excitation wavelength (λ_{ex}) of 370 nm.

The emission behavior of **FTN-Pol** might be attributed to two effects i) a positive solvatochromism indicates a charge transfer in the excited states of the polymer and ii) the solvent has a distinct influence on the dispersion stability and aggregation behavior of the polymer.[7,8,10] Especially aggregation can change the emission properties of dispersed materials quite significantly (e.g., via an increased efficiency of vibronic coupling)[11] and may also in our case significantly contribute to the observed differences in the emission behavior.[12] This is most likely also the reason why the red-shift of the emission spectra does not strictly

follow the solvent polarity (i.e., in the case of MeOH and CH_2Cl_2). The decreased PLQYs in this case indicate the stabilization of the excited state exhibiting charge-transfer or high dipole character.[13]

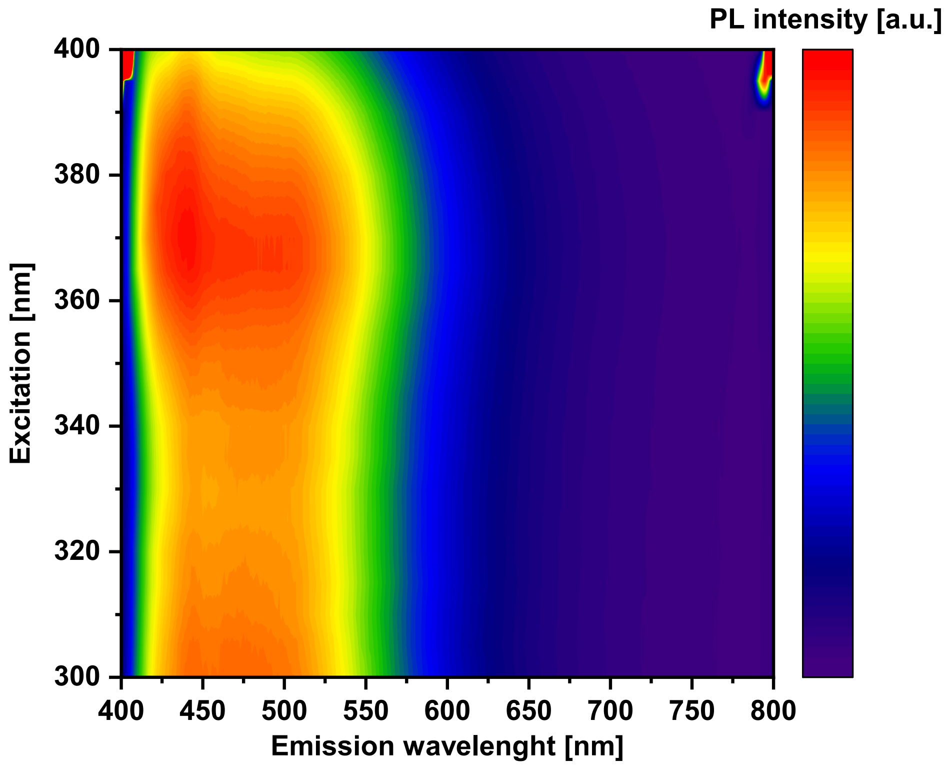

Figure S31: Excitation-emission map of **FTN-Pol** measured as dispersion in cyclohexane.

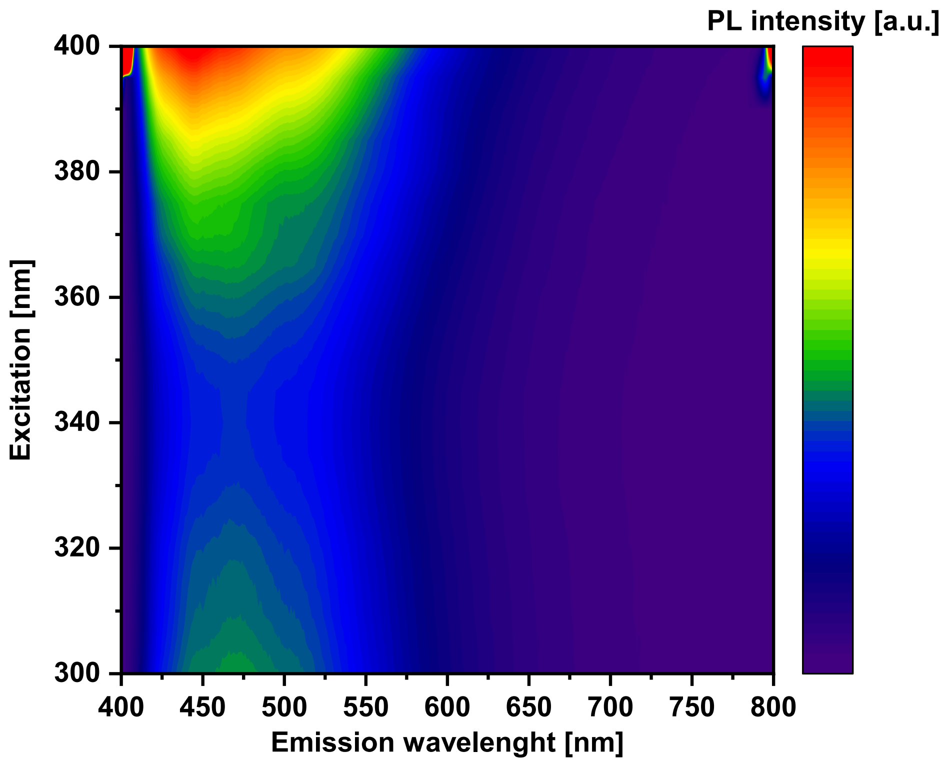

Figure S32: Excitation-emission map of **FTN-Pol** measured as dispersion in toluene.

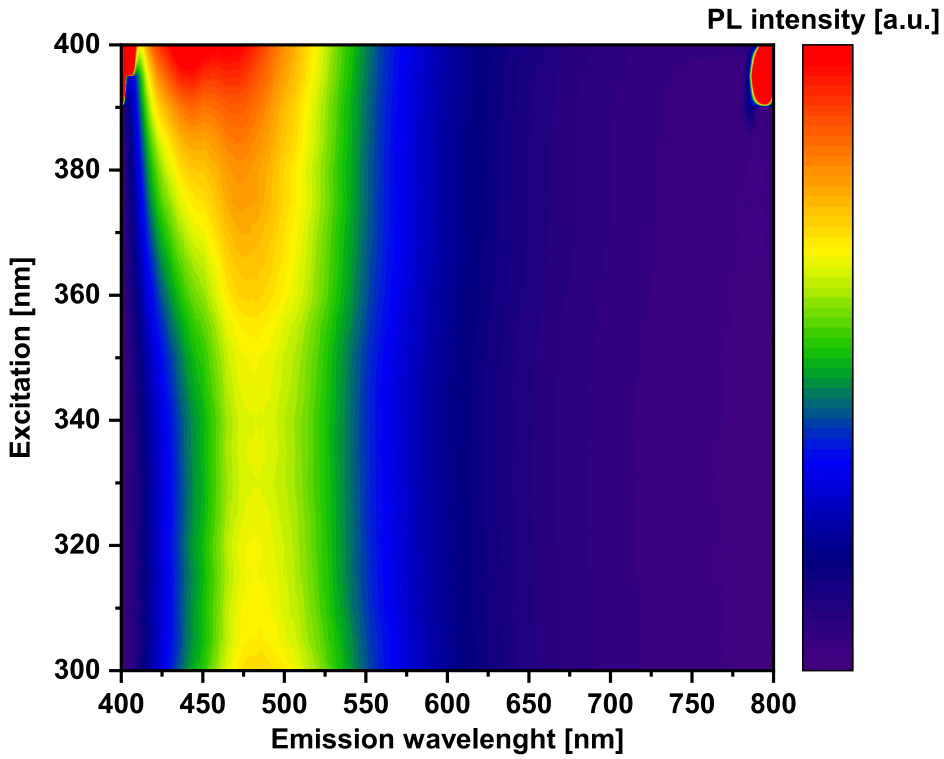

Figure S33: Excitation-emission map of **FTN-Pol** measured as dispersion in MeOH.

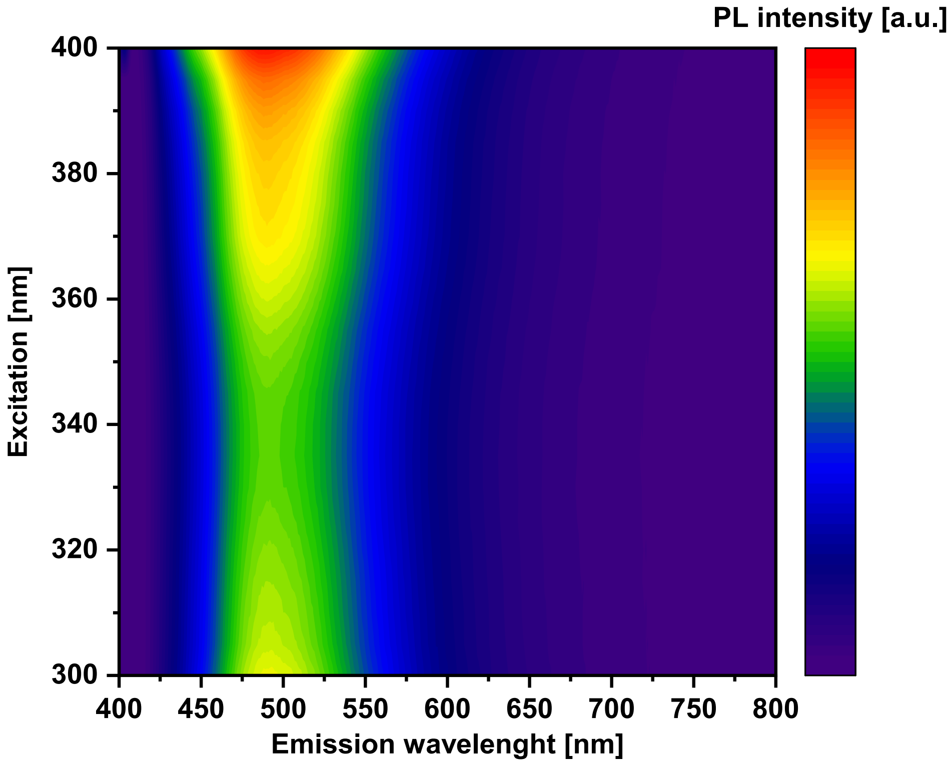

Figure S34: Excitation-emission map of **FTN-Pol** measured as dispersion in THF.

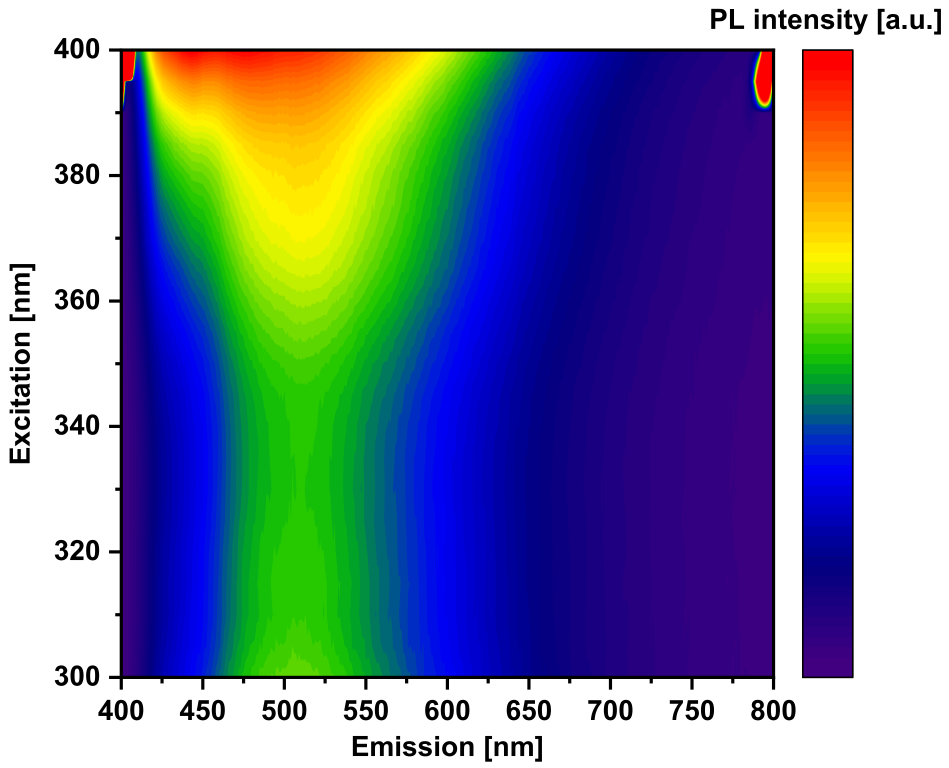

Figure S35: Excitation-emission map of **FTN-Pol** measured as dispersion in DMF.

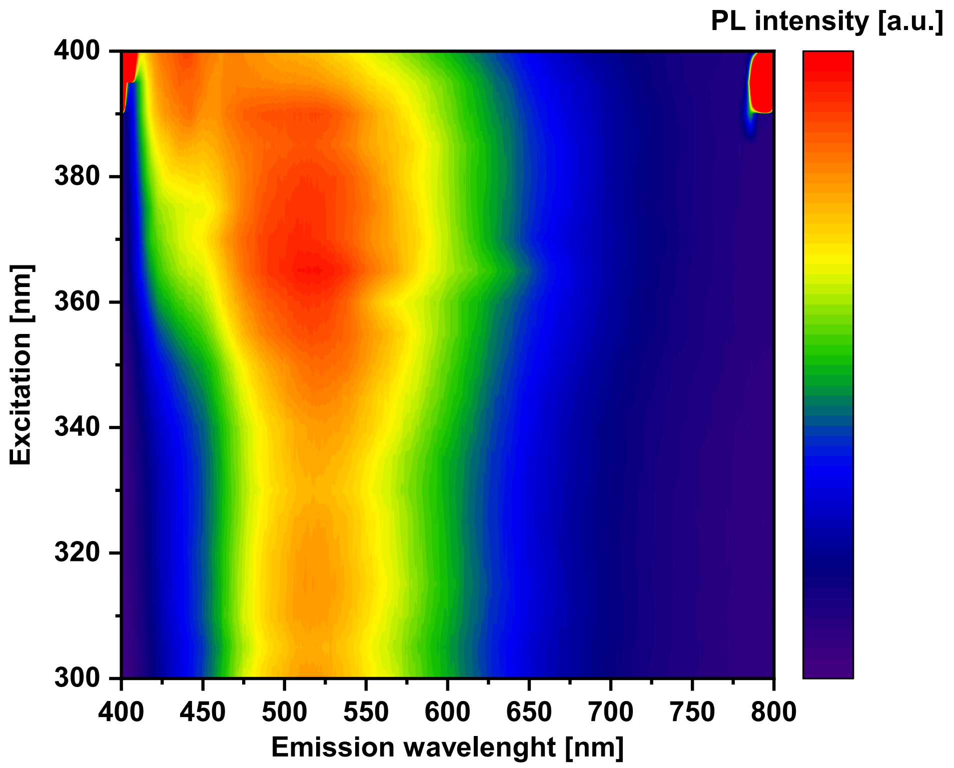

Figure S36: Excitation-emission map of **FTN-Pol** measured as dispersion in MeCN.

.

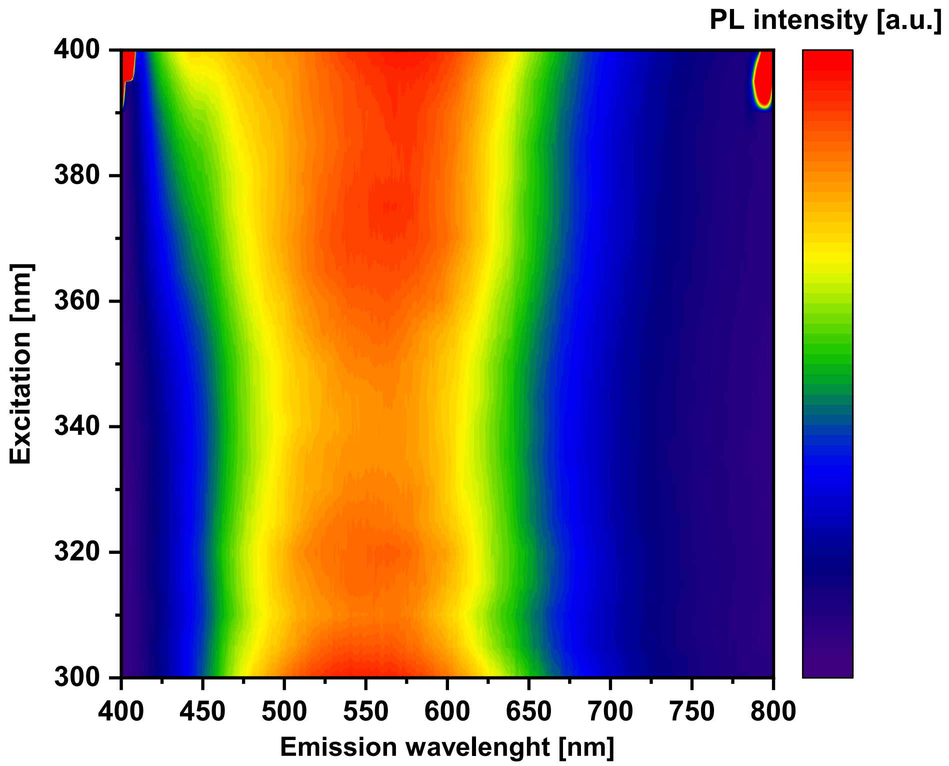

Figure S37: Excitation-emission map of **FTN-Pol** measured as dispersion in CH_2Cl_2.

10. SEM Images

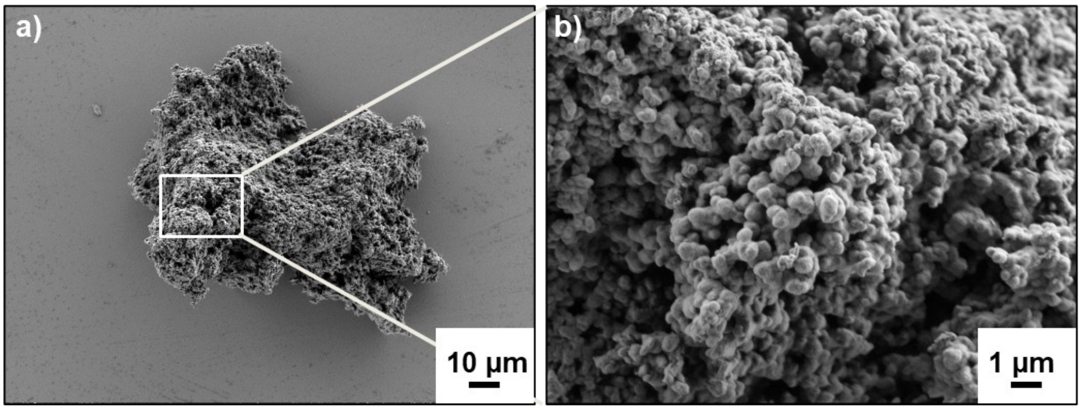

Figure S38: SEM micrographs of the polymer **FTN-Pol** measured as powder.

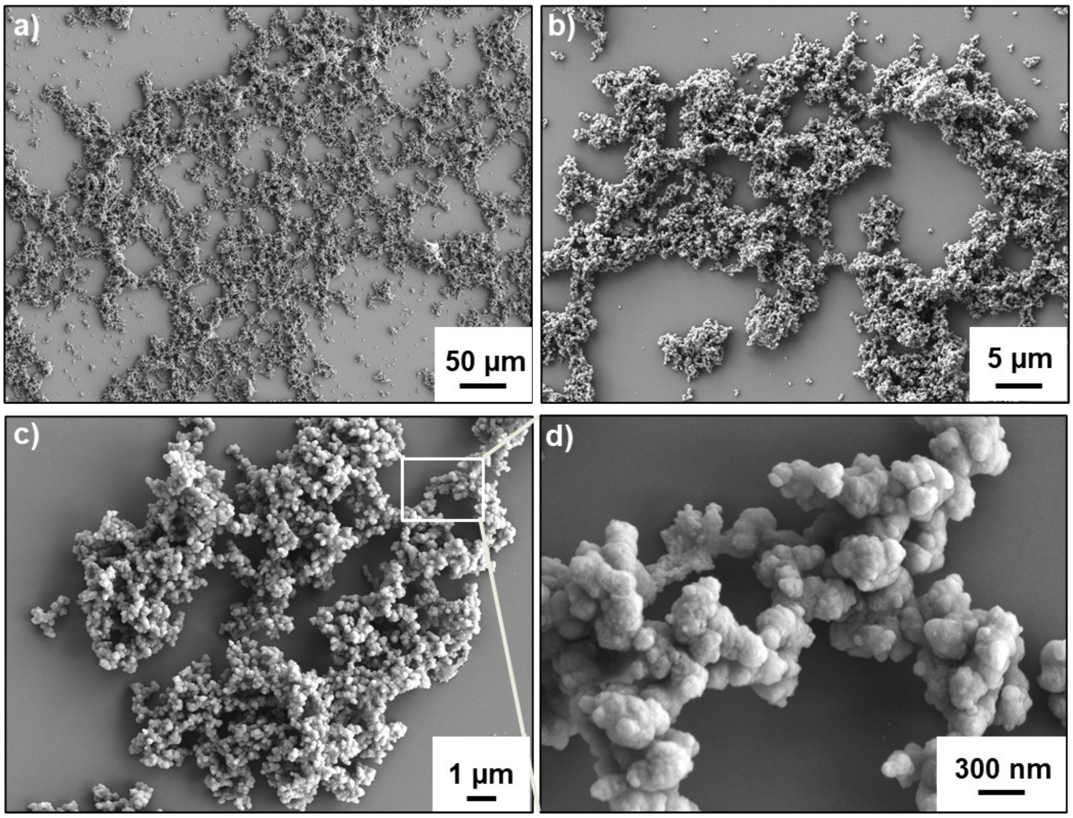

Figure S39: SEM micrographs of the polymer **FTN-H-Pol** measured as powder.

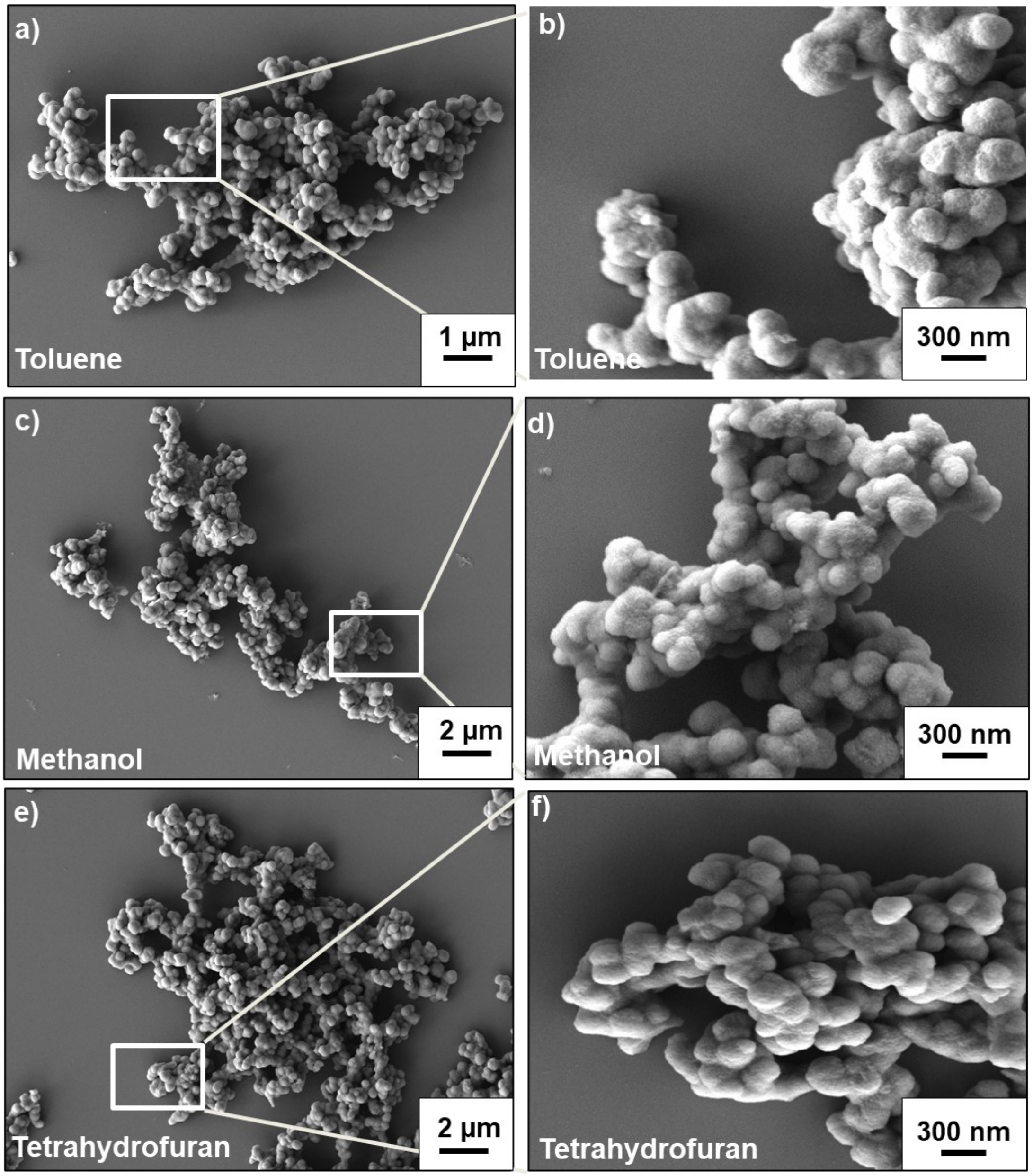

Figure S40: SEM images of **FTN-Pol** from dispersions in different solvents. (a,b) toluene; (c,d) methanol; (e,f) tetrahydrofuran.

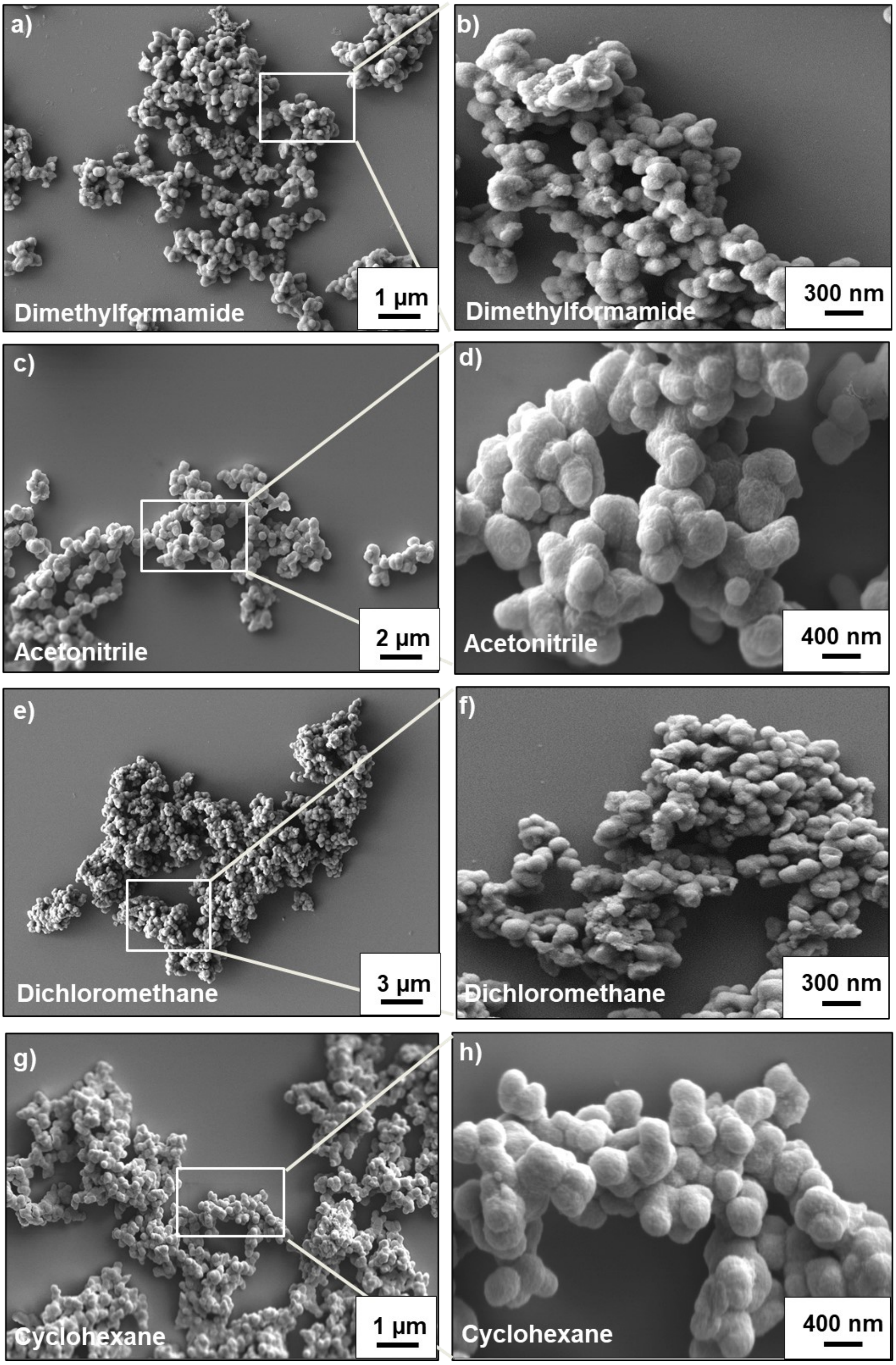

Figure S41: SEM images of **FTN-PoI** from dispersions with different solvents. (a,b) *N*,*N*-dimethylformamide; (c,d) acetonitrile; (e,f) dichloromethane, (g,h) cyclohexane.

11. Time-of-Flight Secondary Mass Spectrometric Analysis

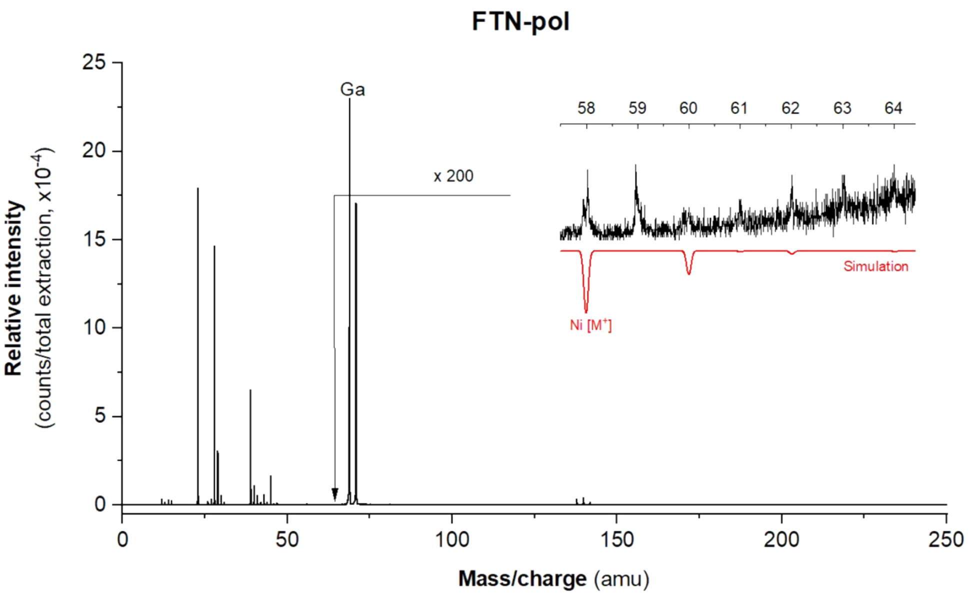

Figure S42: Tof-SIMS of **FTN-Pol** and simulated Tof-SIMS of elemental nickel.

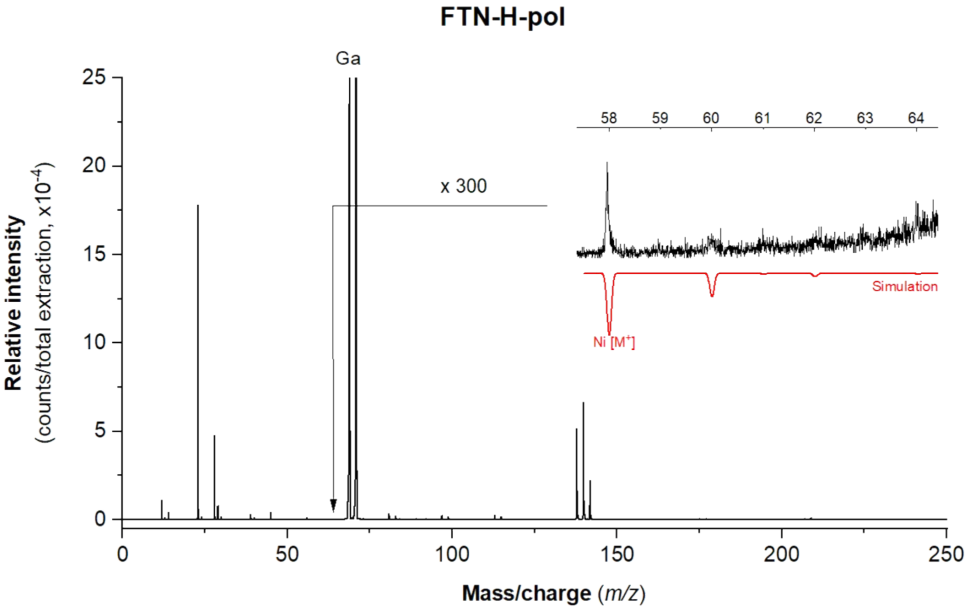

Figure S43: Tof-SIMS of **FTN-H-Pol** and simulated Tof-SIMS of elemental nickel.

12. Gas Sorption Analysis

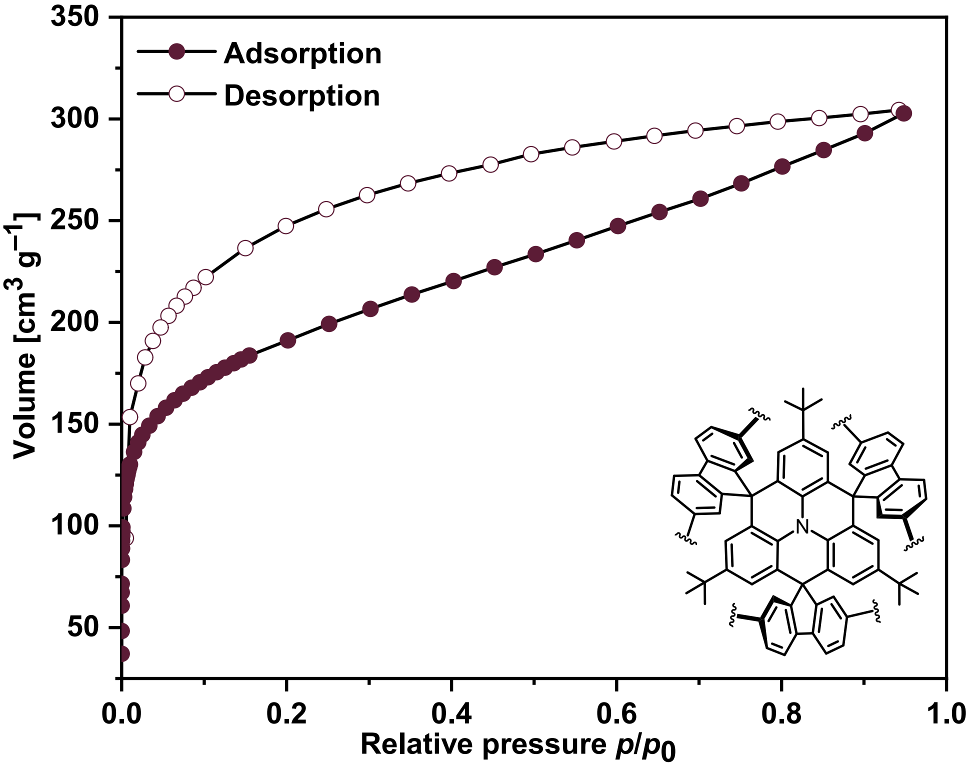

Figure S44: Nitrogen adsorption/desorption isotherm of **FTN-Pol** at 77 K.

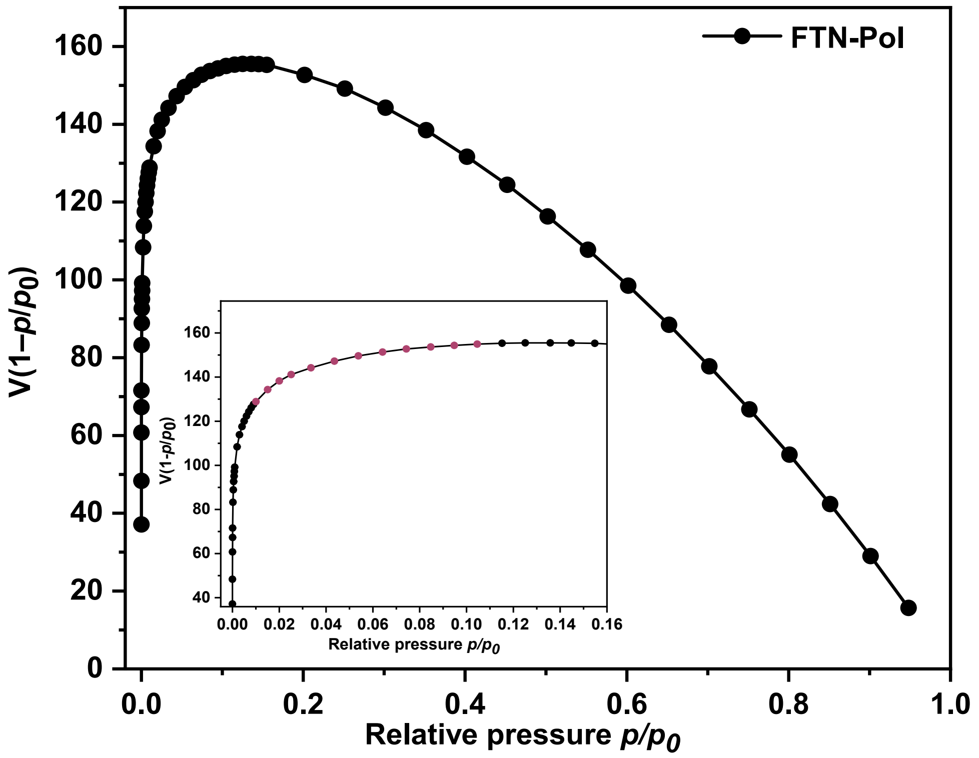

Figure S45: Rouquerol plot of **FTN-Pol** at 77 K. The BET area is highlighted in violet.

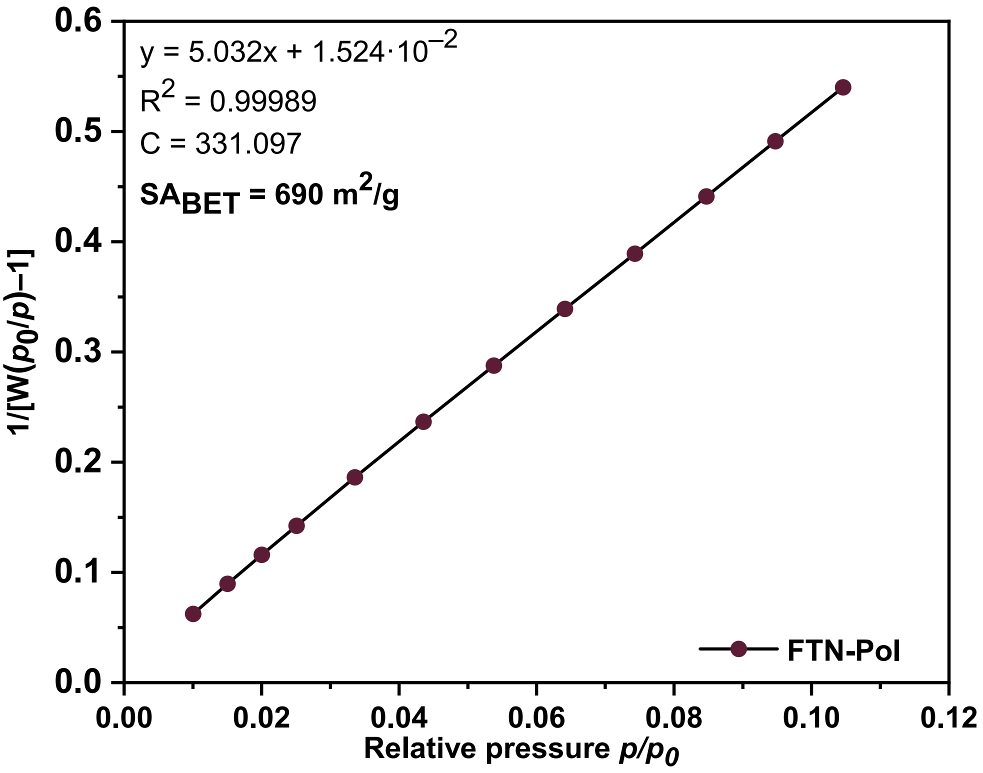

Figure S46: BET plot of **FTN-Pol** at 77 K.

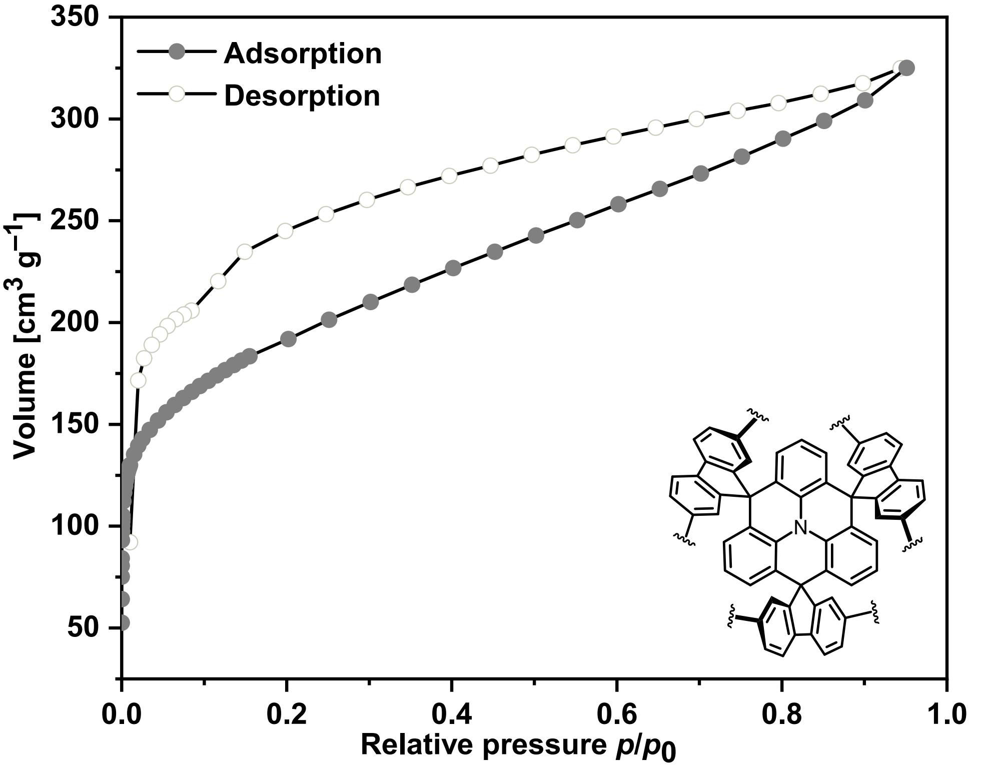

Figure S47: Nitrogen adsorption/desorption isotherm of **FTN-H-Pol** at 77 K.

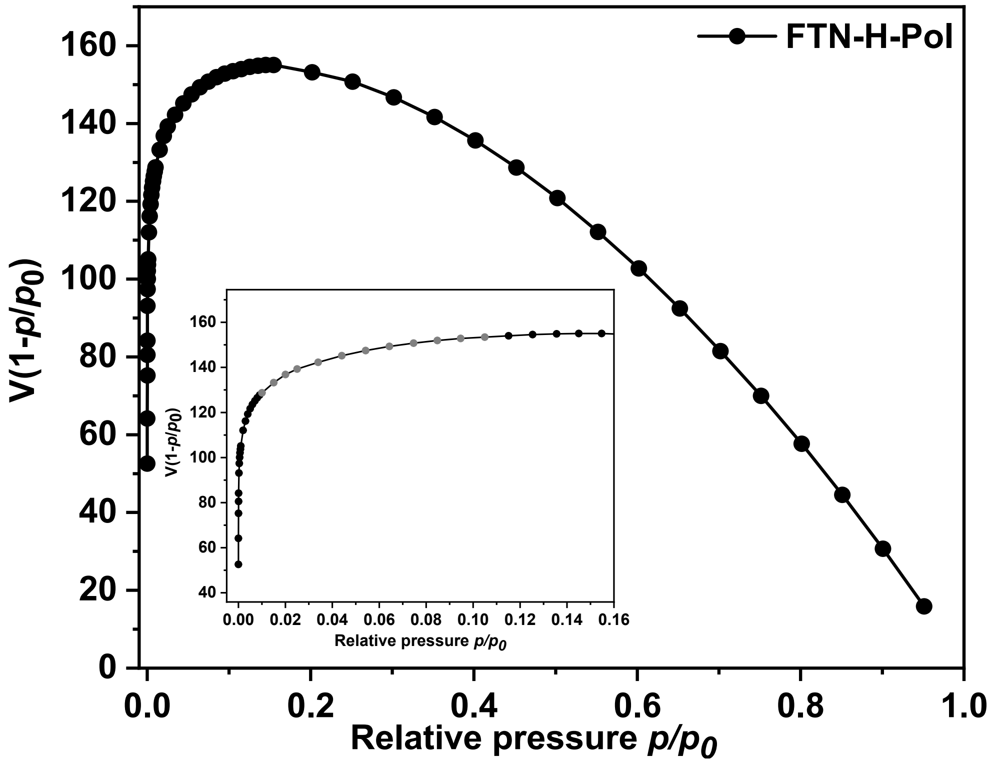

Figure S48: Rouquerol plot of **FTN-H-Pol** at 77 K. The BET area is highlighted in grey.

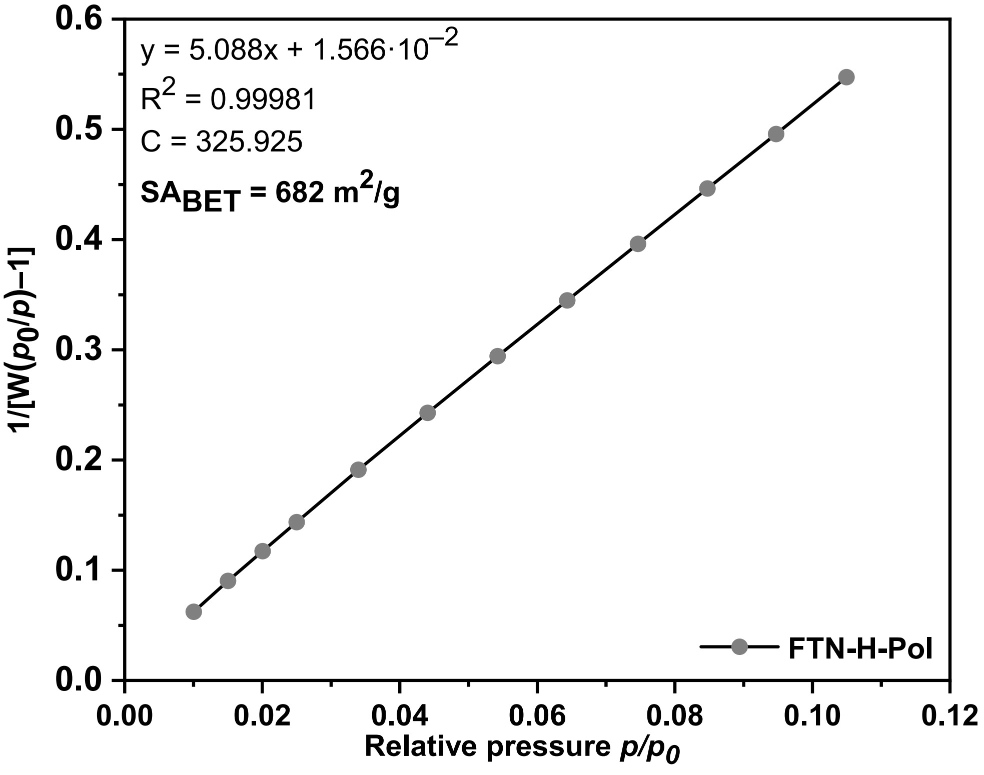

Figure S49: BET plot of **FTN-H-Pol** at 77 K.

13. Electrochemistry Measurements

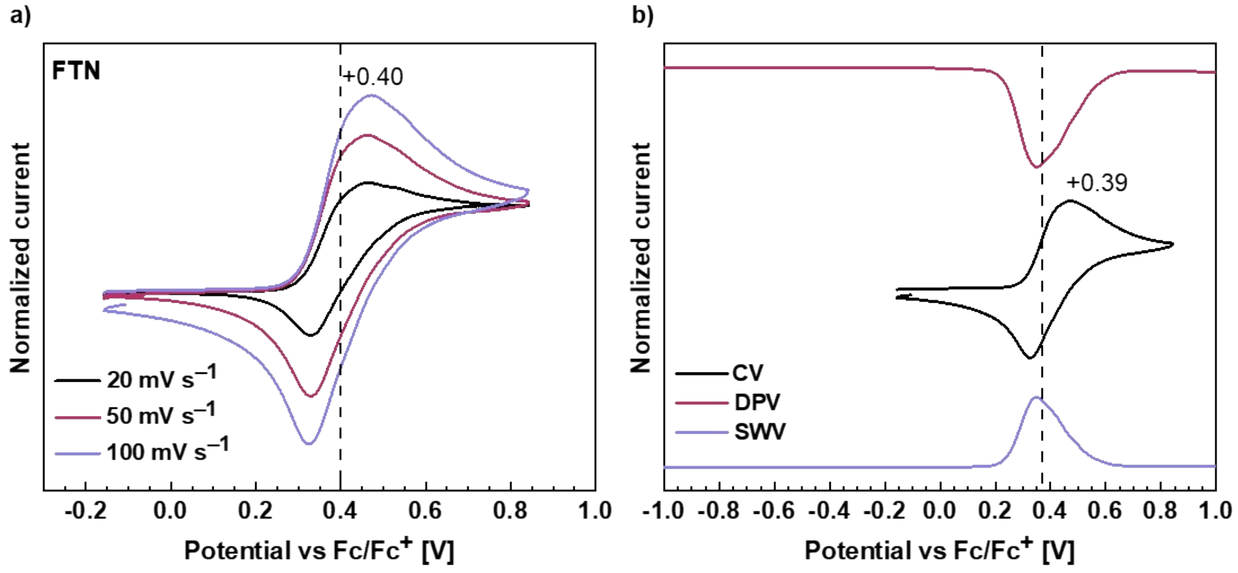

Figure S50: a) CV data of **FTN** measured with different scan rates (20, 50, 100 mV s^{-1}). b) CV, DPV, and SWV spectra of **FTN** measured in dry CH_2Cl_2 with 0.1M *n*-Bu_4NPF_6 as supporting electrolyte referenced to Fc/Fc^+ as internal standard.

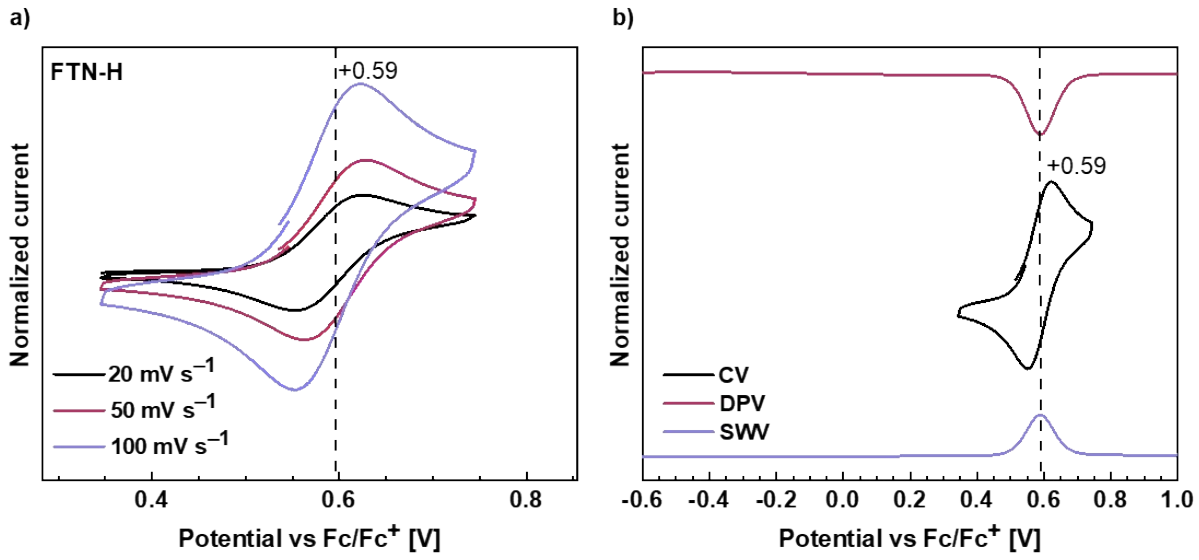

Figure S51: a) CV data of **FTN-H** measured with different scan rates (20, 50, 100 mV s^{-1}). b) CV, DPV, and SWV spectra of **FTN-H** measured in dry CH_2Cl_2 with 0.1M *n*-Bu_4NPF_6 as supporting electrolyte referenced to Fc/Fc^+ as internal standard.

14. Electrode Data

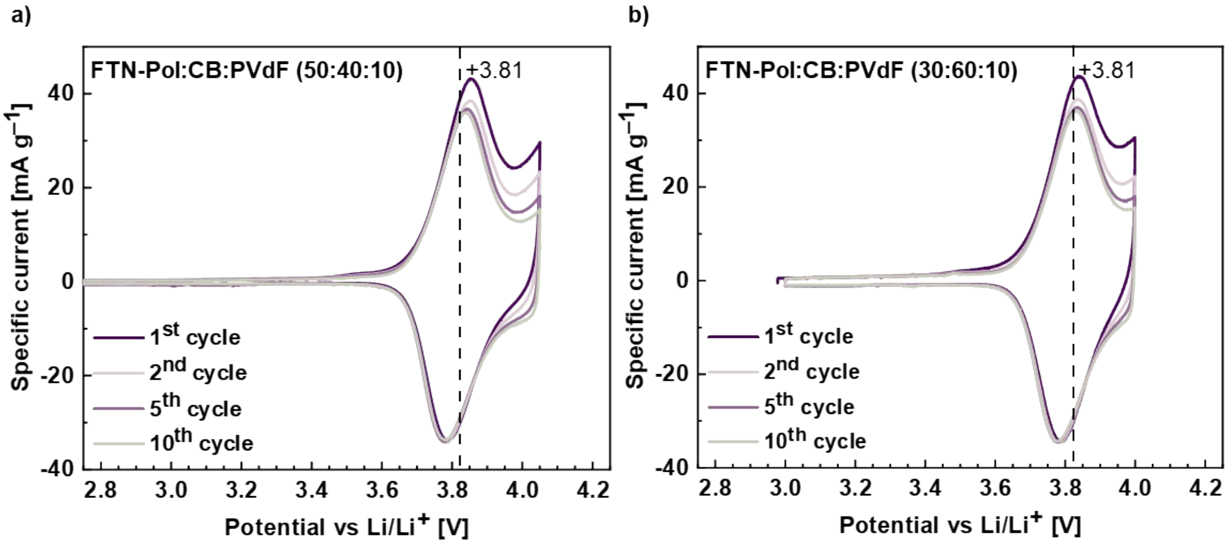

Figure S52: CV data of composite electrodes containing a) **FTN-Pol**:CB:PVdF (50:40:10) or b) **FTN-Pol**:CB:PVdF (30:60:10) measured with a scan rate of 0.1 mV s^{-1} in NMP with 0.1 M $LiPF_6$ as supporting electrolyte and referenced to Li/Li^+ as internal standard. CB = carbon black; PVdF = polyvinylidene fluoride; NMP = *N*-methyl-2-pyrrolidone.

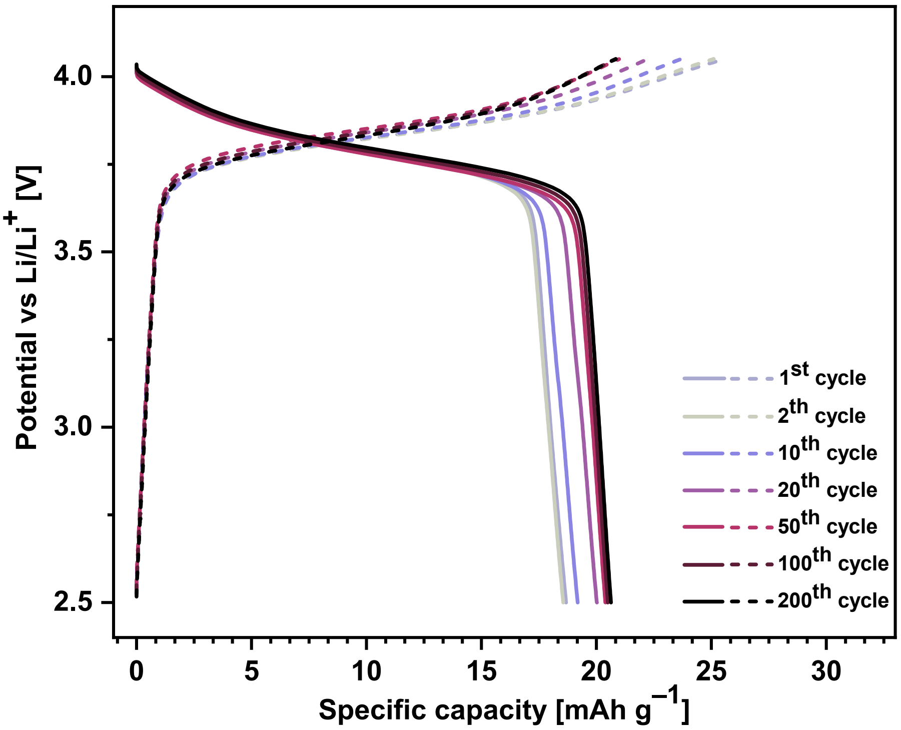

Figure S53: Charge (solid lines) and discharge (dashed lines) curves of **FTN-Pol**-based composite electrode (**FTN-Pol**:CB:PVdF; 50:40:10) measured in the range between 2.5–4.05 V at 100 mA g^{-1}.

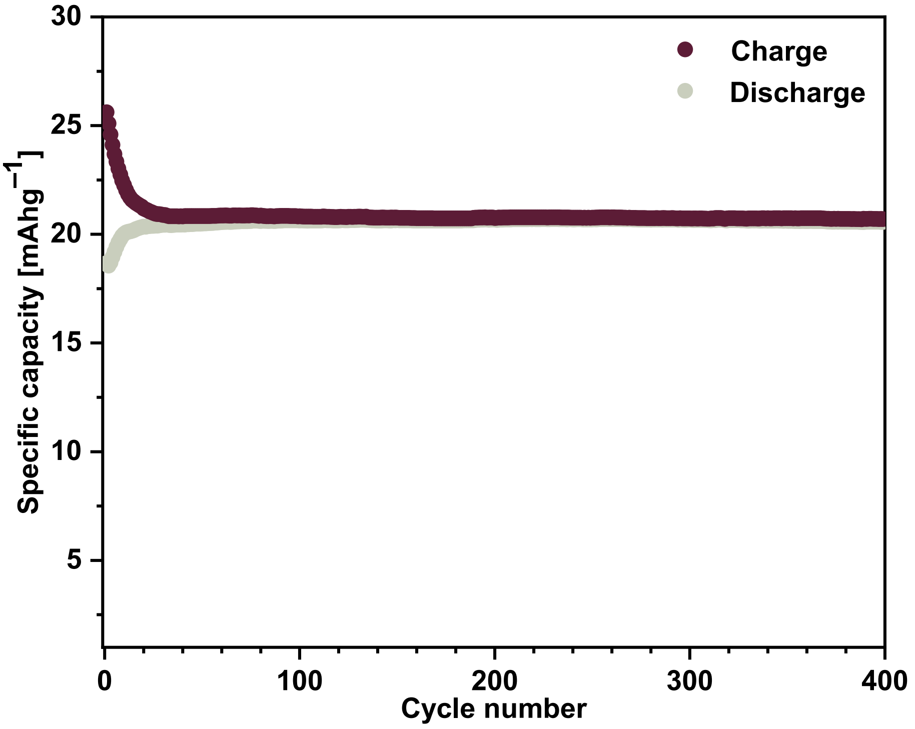

Figure S54: Constant current cycling measurements of **FTN-Pol**-based composite electrode (**FTN-Pol**:CB:PVdF; 50:40:10) measured in the range between 2.5–4.05 V at 100 mA g^{-1}.

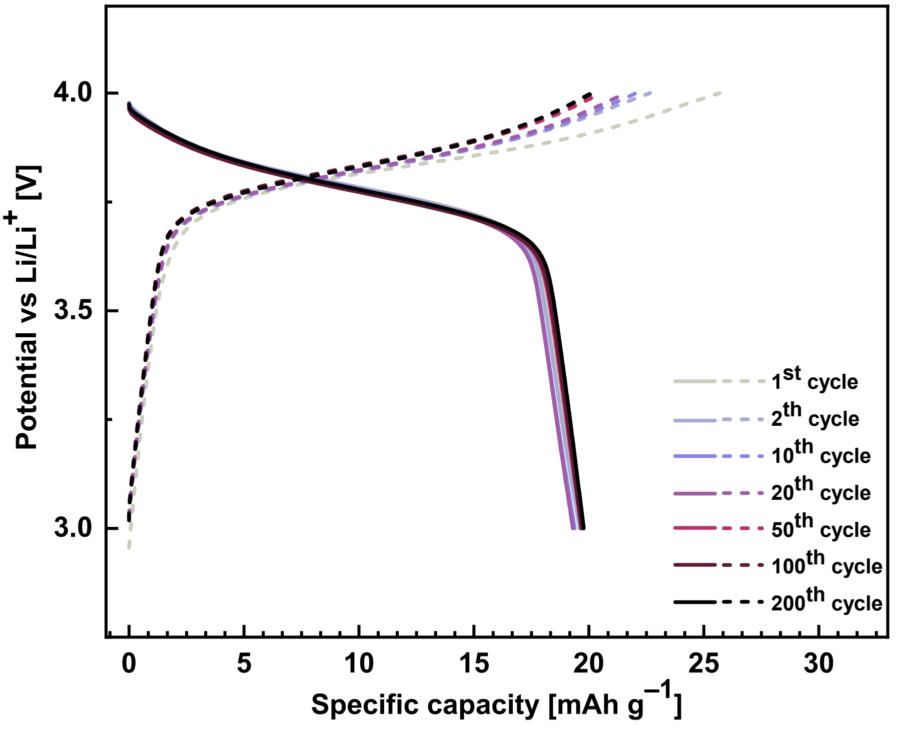

Figure S55: Charge (solid lines) and discharge (dashed lines) curves of **FTN-Pol**-based composite electrode (**FTN-Pol**:CB:PVdF; 30:60:10) measured in the range between 2.5–4.0 V at 100 mA g^{-1}.

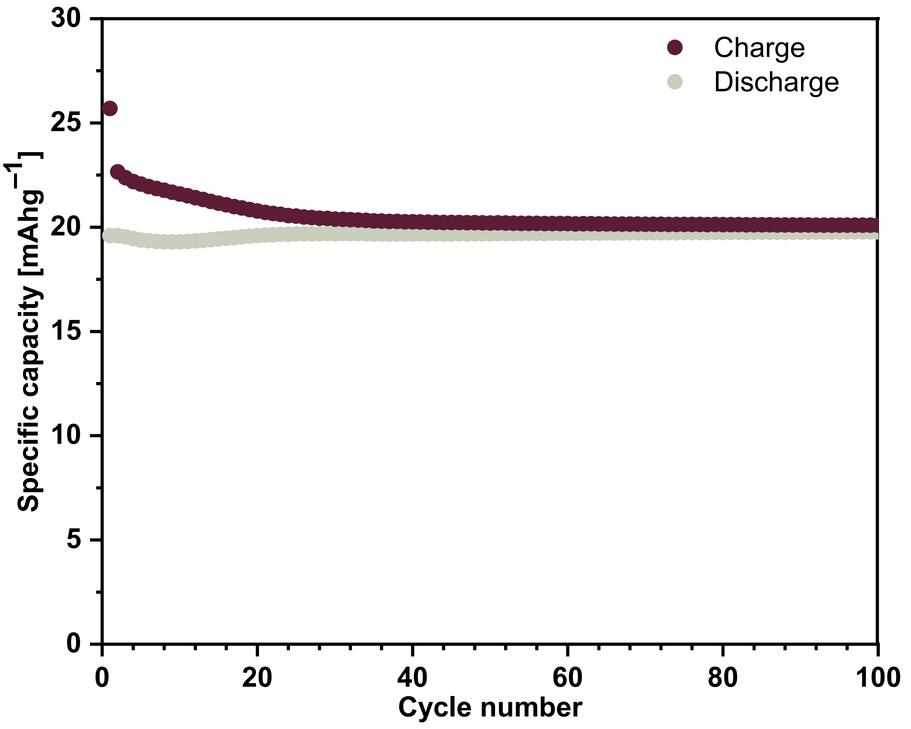

Figure S56: Constant current cycling measurements of **FTN-Pol**-based composite electrode (**FTN-Pol**:CB:PVdF; 30:60:10) measured in the range between 2.5–4.05 V at 100 mA g^{-1}.

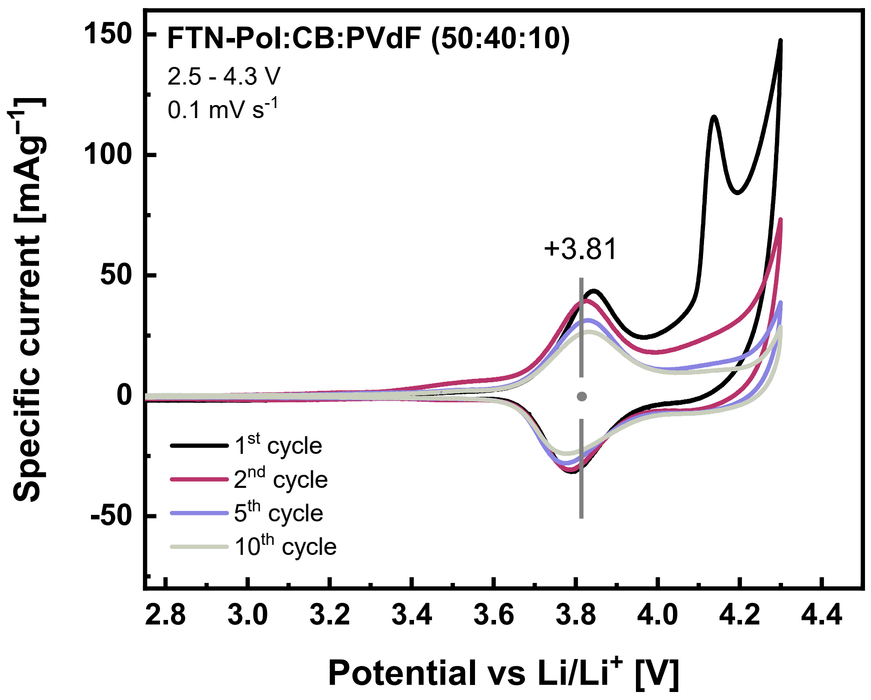

Figure S57: CVs of FTN-Pol based composite electrodes (50 wt%) with 1 M $LiPF_6$, extended voltage range of 2.5–4.3 V, a scan rate of 0.1 mV s^{-1}

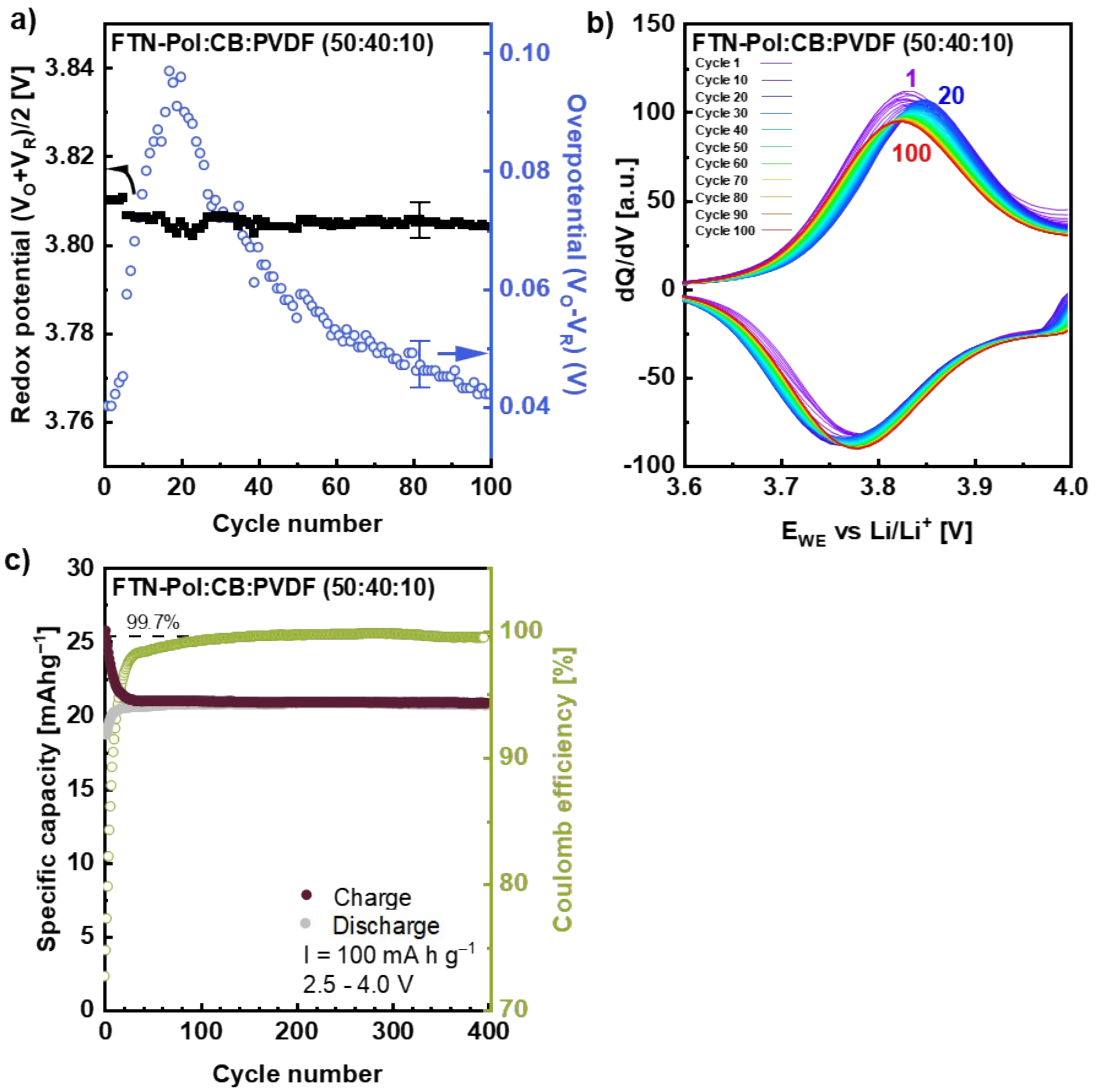

Figure S58: Cycling studies conducted at a current density of 100 mA g^{-1}. a) Redox potential (V_O+V_R)/2 and overpotential (V_O – V_R) of long-time galvanostatic charge/discharge measurement with a current density of 100 mA g^{-1} (ca. 3.3 C) for a voltage range of 2.5 to 4.0 V. b) Derivatives of galvanostatic charge/discharge profiles with a current density of 100 mA g^{-1} for 100 cycles. c) Specific capacity obtained at 100 mA g^{-1} for 400 cycles and normalized to the amount of active material **FTN-Pol**.

Table S4: Summary of the battery properties of **FTN-Pol** and literature-known TPA-based polymers applied as cathode materials.

	C_{theo} [mA h g^{-1}]	*E*ox[a] [V]	$C_{@10}$[b] [mA h g^{-1}]	Coulomb efficiency[c] [%] (C-rate)	Porosity [m^2/g]	Ref.
**TPA-Pol**	111	3.80	91	99.4 (20C)	n.a.	[14]
**SP-TPA**	109	3.60	93	99.0 (1C)	66	[15]
**YP-TPA**	109	3.60	105	99.0 (1C)	544	[15]
**OP-TPA**	109	3.60	73	99.0 (1C)	1557	[15]
**PBDAPA**	154	3.73	133	(n.a.)	130	[16]
**PTDAPTz**	109	3.80	75	(n.a)	930	[17]
**FTN-Pol**	30	3.81	22	99.6 (3C)	690	This work

[a] vs Li/Li$^+$; [b]after the first 10 cycles; [c]after the first 100 cycles.

15. References

1 T. A. Schaub, T. Mekelburg, P. O. Dral, M. Miehlich, F. Hampel, K. Meyer, and M. Kivala, *Chem. Eur. J.*, 2020, **26**, 3264–3269.

2 A. Jocic, D. Galindo, A. Weidlich, J. Zerhoch, F. Rominger, T. Buckup, F. Deschler, A. Dreuw, and M. Kivala, *Adv. Opt. Mater.*, 2024, **12**, 2401656.

3 G. R. Fulmer, A. J. M. Miller, N. H. Sherden, H. E. Gottlieb, A. Nudelman, B. M. Stoltz, J. E. Bercaw and K. I. Goldberg, *Organometallics*, 2010, **29**, 2176–2179.

4 a) G. M. Sheldrick, *Acta Crystallogr. A*, 2015, **71**, 3–8; b) G. M. Sheldrick, *Acta Crystallogr. C*, 2015, **71**, 3–8.

5 C. Lastoskie, K. E. Gubbins and N. Quirke, *J. Phys. Chem.*, 1993, **97**, 4786–4796.

6 M. Krug, M. Wagner, T. A. Schaub, W.-S. Zhang, C. M. Schüßlbauer, J. D. R. Ascherl, P. W. Münich, R. R. Schröder, F. Gröhn, P. O. Dral, M. Barbatti, D. M. Guldi and M. Kivala, *Angew. Chem. Int. Ed.*, 2020, **59**, 16233–16240.

7 N. K. Allampally, A. Florian, M. J. Mayoral, C. Rest, V. Stepanenko and G. Fernández, *Chem. Eur. J.*, 2014, **20**, 10669–10678.

8 T. B. Raju, P. Gopikrishna, J. V. Vaghasiya, S. S. Soni and P. K. Iyer, *J. Photochem. Photobiol. A: Chem.*, 2019, **376**, 12–21.

9 a) A. Iagatti, B. Patrizi, A. Basagni, A. Marcelli, A. Alessi, S. Zanardi, R. Fusco, M. Salvalaggio, L. Bussotti and P. Foggi, *Phys. Chem. Chem. Phys.*, 2017, **19**, 13604–13613; b) C. Kato, S. Nishihara, R. Tsunashima, Y. Tatewaki, S. Okada, X.-M. Ren, K. Inoue, D.-L. Long and L. Cronin, *Dalton Trans.*, 2013, **42**, 11363–11366; c) U. Mayer, V. Gutmann, W. Gerger, *Monatsh. Chem.*, 1975, **106**, 1235–1257; d) A. Jezuita, P. A. Wieczorkiewicz, H. Szatylowicz and T. M. Krygowski, *ACS Omega*, 2021, **6**, 18890–18903; e) J. Tomasi, B. Mennucci and R. Cammi, *Chem. Rev.*, 2005, **105**, 2999–3094.

10 a) R. Steyrleuthner, M. Schubert, I. Howard, B. Klaumünzer, K. Schilling, Z. Chen, P. Saalfrank, F. Laquai, A. Facchetti and D. Neher, *J. Am. Chem. Soc.*, 2012, **134**, 18303–18317; b) M. Belletête, J. Bouchard, M. Leclerc and G. Durocher, *Macromolecules*, 2005, **38**, 880–887.

11 J. I. Paredes, S. Villar-Rodil, A. Martínez-Alonso and J. M. D. Tascón, *Langmuir*, 2008, **24**, 10560–10564.

12 J. M. Hughes, Y. Hernandez, D. Aherne, L. Doessel, K. Müllen, B. Moreton, T. W. White, C. Partridge, G. Costantini, A. Shmeliov, M. Shannon, V. Nicolosi and J. N. Coleman, *J. Am. Chem. Soc.*, 2012, **134**, 12168–12179.

13 a) S. Ji, J. Yang, Q. Yang, S. Liu, M. Chen and J. Zhao, *J. Org. Chem.*, 2009, **74**, 4855–4865; b) Z.-Q. Liang, X.-M. Wang, G.-L. Dai, C.-Q. Ye, Y.-Y. Zhou and X.-T. Tao, *New J. Chem.*, 2015, **39**, 8874–8880.

14 J. K. Feng, Y. L. Cao, X. P. Ai, and H. X. Yang, *J. Power Sources*, *2008*, **177**, 199–204.

15 C. Zhang, X. Yang, W. Ren, Y. Wang, F. Su, and J.-X. Jiang, *J. Power Sources*, *2016*, **317**, 49–56.

16 C. Su, B. Han, J. Ma, and L. Xu, *Chem. Electro Chem.*, 2020, **7**, 4101–4107.

17 Z. Chen, C. Su, X. Zhu, R. Xu, L. Xu, and C. Zhang, J. Polym. Sci., Part A: Polym. Chem., *2018*, **56**, 2574–2583.
